# Loss of oxytocin receptor function disrupts neural signatures of pair bonding and fidelity in the nucleus accumbens

**DOI:** 10.1038/s41467-026-76876-8

**Published:** 2026-09-12

**Authors:** Kimberly L. P. Long, Nerissa E. G. Hoglen, Alex J. Keip, Robert M. Klinkel, DéJenaé L. See, Joseph Maa, Jenna C. Wong, Michael Sherman, Devanand S. Manoli

**Affiliations:** 1 Department of Psychiatry and Behavioral Sciences, University of California, San Francisco, San Francisco, CA USA; 2 Center for Integrative Neuroscience, University of California, San Francisco, San Francisco, CA USA; 3 Weill Institute for Neurosciences, University of California, San Francisco, San Francisco, CA USA; 4 Kavli Institute for Fundamental Neuroscience, University of California, San Francisco, San Francisco, CA USA; 5 Neurosciences Graduate Program, University of California, San Francisco, San Francisco, CA USA; 6 Department of Molecular and Cell Biology, University of California, Berkeley, Berkeley, CA USA

**Keywords:** Social behaviour, Neural circuits

## Abstract

Enduring relationships dramatically shape behaviors by promoting affiliation between familiar individuals; however, the neural mechanisms driving attachments remain unclear. Oxytocin receptor (Oxtr) signaling in the nucleus accumbens (NAc) reinforces pair bond formation in socially monogamous prairie voles. How Oxtr influences NAc activity during affiliative behaviors with partners and rejection of strangers is unknown. Using in vivo fiber photometry, we demonstrate that genetic loss of Oxtr sex-specifically disrupts attachment behaviors and associated NAc activity. In females, loss of Oxtr reduces prosocial behaviors and NAc activity during initial chemosensory investigation of novel males; however, partner-directed affiliative behavior and associated activity increase independently of Oxtr. In males, loss of Oxtr increases prosocial investigation of novel females, and initial behavioral and neural activity patterns predict future partner preference in Oxtr-null males. These findings reveal sex-specific roles for Oxtr in early pair bond development through modulation of prosociality and sensory processing.

## Abstract (web summary)

Oxytocin receptor signaling in the nucleus accumbens supports pair bonding, but its role in attachment-related neural activity is unclear. Here, the authors show that Oxtr loss sex-specifically alters pair bonding and nucleus accumbens activity in prairie voles; in males, early activity predicts partner preference.

## Introduction

Long-term attachments between individuals are one of the most intriguing forms of social behavior and are central to human interactions, from parent-child bonds to enduring relationships between mates^1–3^. Despite the importance of attachment for the organization of complex social structures across species^4,5^, the neural mechanisms mediating these behaviors remain poorly understood. Seminal work in socially monogamous prairie voles revealed that oxytocin and the oxytocin receptor (Oxtr) are key modulators of pair bonding, *i.e*., the formation of selective and enduring attachments between mates. Pair-bonded animals demonstrate both a preference for a bonded partner (partner preference) and active rejection of novel potential mates (strangers)^6^. Exogenous oxytocin in the brain facilitates the formation of partner preference in both male and female prairie voles^7,8^, while pharmacological inhibition of Oxtr disrupts the display of partner preference after mating^9^. Defining the neural circuits that govern pair bonding and determining how they are regulated by Oxtr are key to understanding social attachment behaviors. We recently demonstrated that, strikingly, prairie voles lacking Oxtr display partner preference^10^. However, Oxtr mutants display delayed development of partner preference and increased prosocial behavior towards strangers, suggesting that Oxtr influences the patterns of social interactions that facilitate pair bonding and controls the rejection of strangers^11^.

A key site of oxytocin action in the brain is the nucleus accumbens (NAc). The NAc has long been implicated in the reinforcement of behaviors ranging from addiction to innate displays associated with social interactions, including mating, aggression, and reciprocal interactions that mediate enduring attachments between mates^12–18^. The NAc integrates input from regions including the prefrontal cortex, thalamus, and amygdala as well as dopaminergic input from the ventral tegmental area to regulate diverse functions associated with reward- and survival-related behaviors^12,19–22^. Neuromodulatory signals—including oxytocin from the paraventricular nucleus of the hypothalamus and serotonin from the dorsal raphe—are also integrated within the NAc to influence prosocial behaviors and social reward^23–28^. In parallel to the reinforcement of rewarding stimuli, subregions of the NAc also appear to mediate responses to aversive stimuli, suggesting that components of the mesocorticolimbic system may control prosocial as well as agonistic interactions between individuals^29,30^. Compared to closely related but promiscuous vole species, prairie voles exhibit dramatically enriched oxytocin binding in the NAc^31–33^, and knockdown of Oxtr expression specifically within the NAc disrupts pair bonding^34^. Neuronal activity in the NAc in prairie voles evolves as a pair bond develops, such that ensembles responding to partner approach expand in size over time^35^. However, it remains unclear how the NAc responds to complex social interactions in the context of attachment and how such activity is modulated by Oxtr signaling.

Here, we utilize in vivo, longitudinal fiber photometry to examine NAc calcium activity across pair bond development in male and female, wild-type (WT) and Oxtr null (*Oxtr*^*1−/−*^) prairie voles. We demonstrate that the NAc responds to various types of social interaction and that loss of Oxtr signaling alters both pair-bonding behaviors and behavior-related NAc activity in a sex-specific manner. Together, these results demonstrate that Oxtr is critical for the development of pair bonds by modulating prosociality and neural processing of sensory and social interactions with novel individuals.

## Results

### Loss of Oxtr sex-specifically alters NAc neural responses of naïve prairie voles to novel partners

**Fig. 1:**
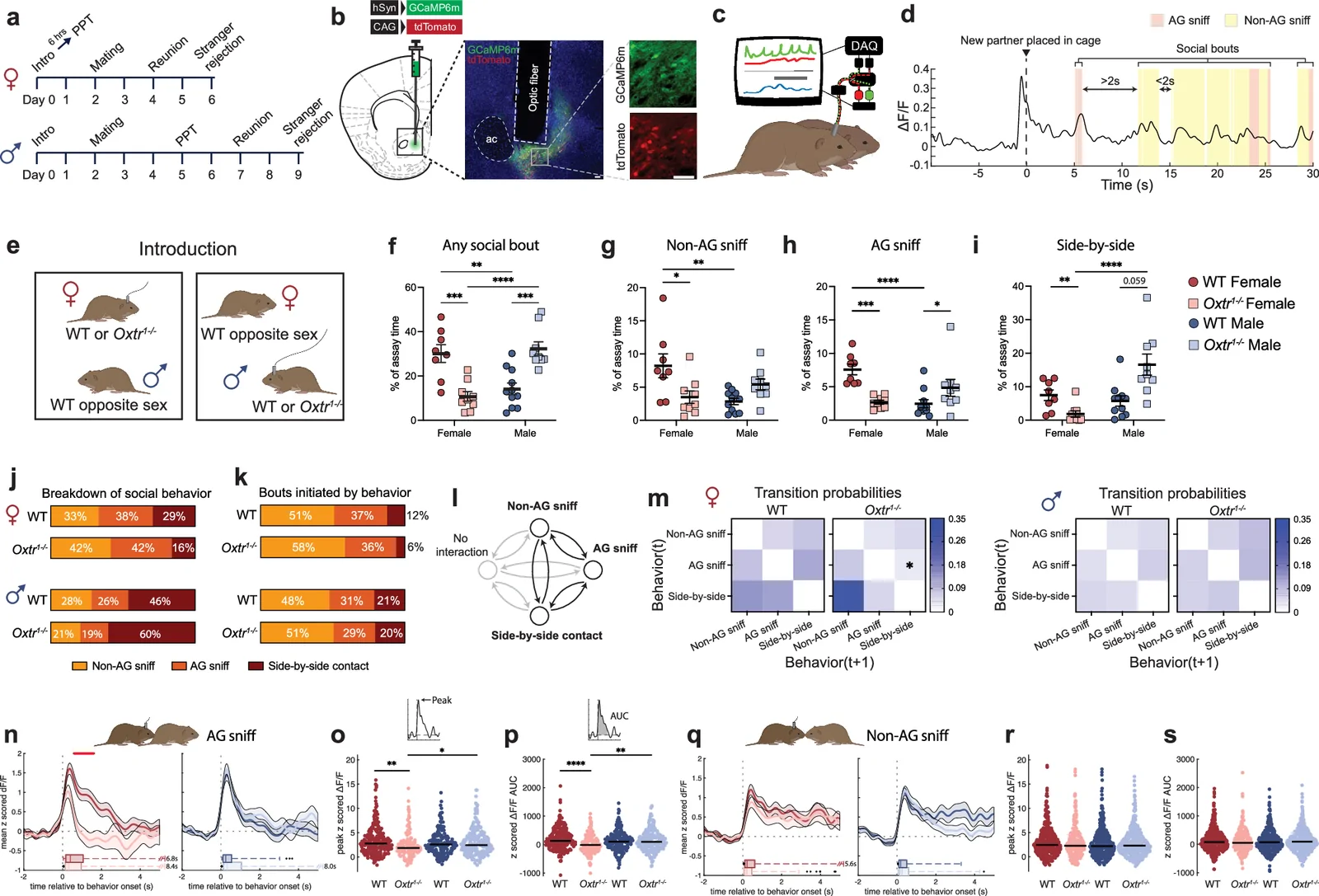
Loss of Oxtr in naive females disrupts NAc neural responses to novel males. **a** Timeline of assays. Intro: Introduction, PPT: Partner preference test. **b** Injection and fiber targeting in the medial NAc (scale bar, 50 um) (ac, anterior commissure). **c** Photometry setup. **d** Example GCaMP6m trace at the start of the introduction with behavior events overlaid (anogenital [AG] sniff, orange; non-AG sniff, yellow). Behavior events separated by less than 2 s were considered part of a single social bout. **e** Introduction procedure. A wild-type (WT) or *Oxtr*^*1−/−*^ prairie vole was placed into a clean cage, and a WT, opposite sex vole was then introduced. **f** Total percent of assay time engaged in social bouts exhibited by the focal vole (WT_Female_ *n* = 8 voles, *Oxtr*^*1−/−*^_Female_ *n* = 9, WT_Male_ *n* = 11, *Oxtr*^*1−/−*^_Male_ *n* = 9; two-sided two-way ANOVA, Sex by Genotype, F_1,33_ = 40.42, *p* < 0.0001, Sidak post hoc comparisons). **g–i** Percent of introduction assay time spent engaged in non-anogenital (AG) sniffing (**g**, two-way ANOVA, Sex by Genotype, F_1,33_ = 13.62, *p* = 0.0008, Sidak post hoc comparisons), AG sniffing (h, two-sided two-way ANOVA, Sex by Genotype, F_1,33_ = 28.05, *p* < 0.0001, Sidak post hoc comparisons), or side-by-side contact (i, two-sided two-way ANOVA, Sex by Genotype, F_1,33_ = 21.58, *p* < 0.0001, Sidak post hoc comparisons) (WT_Female_ *n* = 8, *Oxtr*^*1−/−*^_Female_ *n* = 9, WT_Male_ *n* = 11, *Oxtr*^*1−/−*^_Male_ *n* = 9). **j** Mean breakdown of social contact by the percentage of contact time engaged in AG sniffing, non-AG sniffing, and side-by-side contact (linear mixed effects model [LME], Sex by Genotype by Behavior, *p* = 0.0045). **k** Percentages of social bouts initiated with non-AG sniff, AG sniff, or side-by-side contact (LME, Sex by Genotype by Behavior, *P* = 0.65). **l** A schematic of Markov modeling of behavior, focusing on transitions from one social behavior to another. **m** Heat maps of female (left) and male (right) transition probabilities (two-sided permutation tests on F statistics with Sidak corrections, Sex by Genotype, F_1,33_ = 14.2, *p* = 0.011, Sidak-corrected post hoc t tests). **n** Mean peri-event time histogram (PETH) of z-scored GCaMP6m ΔF/F by sex and genotype aligned to the onset of AG sniffs (WT_Female_ *n* = 393 traces; *Oxtr*^*1−/−*^_Female_ *n* = 272; WT_Male_ *n* = 358; *Oxtr*^*1−/−*^_Male_ *n* = 435). Data are presented as mean values ± SEM. Box plot shows the median (center line) of durations of the initiating AG sniffs, interquartile range (box), and whiskers adjusted for skewed data (see “Methods”); outliers are plotted as individual points. **o** Swarm plot of peak z-scored ΔF/F values following AG sniffs (LME, Sex by Genotype, F_1,901_ = 7.14, *p* = 0.0077, Sidak-corrected post hoc LMEs). **p** Area under the curve (AUC) values from z-scored ΔF/F traces following AG sniffs (LME, Sex by Genotype, F_1,901_ = 7.14, *p *= 0.0077, Sidak-corrected post hoc LMEs). **q** Mean PETH by sex and genotype aligned to non-AG sniffs. The red line indicates time points at which mean z-scored ΔF/F differ between WT and *Oxtr*^*1−/−*^ females (WT_Female_ *n* = 269 traces; *Oxtr*^*1−/−*^_Female_ *n* = 160; WT_Male_ *n* = 219; *Oxtr*^*1−/−*^_Male_ *n* = 257). Data are presented as mean values ± SEM. Box plot shows the median (center line) of durations of the initiating non-AG sniffs, interquartile range (box), and whiskers adjusted for skewed data (see “Methods”); outliers are plotted as individual points. **r** Peak z-scored ΔF/F values (LME, Sex by Genotype, F_1,1454_ = 0.53, *p* = 0.47). **s** AUC values from z-scored ΔF/F traces (LME, Sex by Genotype, F_1,1454_ = 1.06, *p* = 0.30). Detailed statistics are presented in Extended Data File 1. *p < 0.05, **p < 0.01, ***p < 0.*p*01, ****p < 0.0001. Vole images were created in BioRender. Manoli, D. (2026) https://BioRender.com/00l1i4c. Source data are provided as a Source Data file.

Our recent findings open questions about the precise role of Oxtr function for the formation of a pair bond^10^. To determine how Oxtr function influences social and attachment behaviors and simultaneous neural activity in the NAc during and following pair bonding, we implemented fiber photometry in WT and *Oxtr*^*1−/−*^ voles of both sexes (Fig. 1a). Specifically, we examined the introduction to a sexually naïve, WT, opposite sex mate (partner); partner preference; mating behavior following estrus induction; acute separation from and reunification with the partner; and exposure to a novel, WT, sexually naïve, opposite sex animal (stranger). Importantly, this sequence allows us to compare dyadic social interactions with novel and familiar animals before and after bond formation^11^.

We virally expressed the fluorescent calcium indicator GCaMP6m under the synapsin promoter in the medial NAc core and shell and implanted an optic fiber over the site of injection (Fig. 1b, and Supplementary Figs. 1, 2). We examined calcium activity within the NAc as WT or *Oxtr*^*1−/−*^ voles of either sex freely interacted with a stimulus animal and engaged in specific social interactions, such as chemosensory investigation, affiliation, mating, aggression, and defensive behaviors (Fig. 1c and Table 1). We then extracted z-scored GCaMP6m fluorescence traces surrounding individual social bouts (social touch preceded by at least 2 s of no interaction, Fig. 1d and Supplementary Fig. 3) and generated peri-event time histograms.

**Table 1:** All behaviors scored during dyadic interactions and their definitions

Behavior	Description/Criteria
Non-anogenital (non-AG) sniff	Social touch in which the snout of the implanted animal makes contact with the face or flank, but not the anogenital zone (see below) of the stimulus animal.
Anogenital (AG) sniff	Social touch in which the snout of the implanted animal makes contact with the anogenital region (closest to the tail and the surrounding hindquarters, excluding the spine and back).
Side-by-side contact	Social touch in which more than 50% of the implanted animal’s flank is in contact with the stimulus animal. No time threshold was stipulated for side-by-side contact. This behavior, particularly longer bouts, is considered affiliative social behavior.
Social bout	A sequence of non-AG sniffing, AG sniffing, and/or side-by-side contact separated by less than 2 s of no interaction.
Mounting	A male vole approaches from behind and places its forequarters on the hindquarters of a female vole. The start of a mount was defined as the moment the male grasps the female’s flank with its forepaws. A mount was scored for a female vole when she received mounting from a male. (Female mounting was extremely rare; we observed one instance out of all females in this study.)
Intromission	Pelvic thrusting of a male while in the mounting position. Intromission was scored for a female when she received intromission.
Strike	A lunge, bite, or kick by the experimental vole directed towards the stimulus animal.
Defensive strike	A lunge or strike by the experimental vole following the receipt of aggression or social touch from a stimulus animal.
Tussle	A highly aggressive rolling fight in which both voles are biting and/or wrestling.
Aggression receipt	The receipt of a strike or defensive strike by the experimental vole from a stimulus vole.
Defensive rear	A defensive posture in which both forepaws are raised off the ground while oriented towards the stimulus vole.

In mice and other rodents, including prairie voles, Oxtr signaling modulates prosocial behavior^8,24,27,36^. Furthermore, Oxtr function differs between the sexes, and loss of Oxtr transcriptionally impacts females and males in different ways^11,37^. To interrogate how the role of Oxtr function may differ in the sexes during prairie voles’ initial interactions with a mate, we examined patterns of behavior and activity in the NAc during the introduction of a naïve vole to a naïve WT partner (introduction, Fig. 1e). This revealed a significant difference in how the loss of Oxtr affects the behavior of females versus males. Compared to WT females, *Oxtr*^*1−/−*^ females exhibited less investigative and affiliative (side-by-side) social interaction with males, reflected in both the total amount of time spent in social interactions and the number of social bouts initiated (Fig. 1f–i and Supplementary Fig. 4a–d; see Supplementary File 1 for statistics regarding this and subsequent analyses). In contrast, *Oxtr*^*1−/−*^ males displayed increased social investigation of female partners when compared to WT males and *Oxtr*^*1−/−*^ females. Moreover, *Oxtr*^*1−/−*^ males engaged in significantly less agonistic behavior (strikes) towards females than WT males, with no mutant males displaying strikes towards females (Supplementary Fig. 4e–k). Chemosensory investigation (*i.e*., sniffing), including anogenital sniffs and sniffs directed to other parts of the body, comprised a large proportion of social interactions and frequently preceded other behaviors (Fig. 1j, k). Markov chain modeling of behavior revealed that *Oxtr*^*1−/−*^ females were less likely than WT females to transition from anogenital investigation to side-by-side contact, a highly prosocial and affiliative behavior that increases with pair bonding (Fig. 1l, m and Supplementary Fig. 4l–o). Thus, loss of Oxtr decreases social and affiliative displays by females to a novel partner and, conversely, increases such displays by males.

We next tested whether neural activity within the NAc, a region that is dramatically enriched for oxytocin binding in monogamous versus promiscuous vole species, is disrupted upon loss of Oxtr. Activity during initial social interactions with novel partners was altered in *Oxtr*^*1−/−*^ voles in a sex- and behavior-specific pattern. *Oxtr*^*1−/−*^ females exhibited decreased peak fluorescence and area under the curve (AUC) at the onset of anogenital investigations of males, while males showed no differences in activity in the NAc associated with this behavior (Fig. 1n–p and Supplementary Fig. 4p). In contrast, activity associated with non-anogenital sniffs or social bouts initiated by side-by-side contact did not differ between either male or female WT and *Oxtr*^*1−/−*^ voles (Fig. 1q–s and Supplementary Fig. 4q–s). The specificity of this difference to chemosensory investigation suggests that the loss of Oxtr influences neural processing of male chemosensory cues in naïve female prairie voles^38–41^.

To test whether neural activity during anogenital investigation predicted subsequent patterns of social behavior, we conducted a mixed effects survival regression to determine the relationship between either normalized peak activity or AUC during a social bout initiated by sniffing (sniff bout) and the latency to subsequent bouts^17^. We found a significant sex by genotype by AUC interaction (see Supplementary Data 1 "Statistics"). *Post hoc* analyses revealed that increased AUC of NAc calcium activity significantly predicted longer latencies in WT, but not *Oxtr*^*1−/−*^, males. No relationship between activity and latency to next behavior was identified in females. Together, these findings suggest that Oxtr function, either during development or in the context of adult social interactions, sex-specifically influences prosocial behavior and neural activity in the NAc of prairie voles. In naïve females, Oxtr function promotes prosocial investigation of novel males and modulates chemosensory-related signaling in the NAc, while in naïve males, Oxtr function appears to reduce prosocial investigation of novel females, facilitate agonistic displays, and regulate the relationship between NAc neural activity associated with chemosensory investigation and subsequent social behaviors.

### Loss of Oxtr modulates neural and behavioral responses to novel males in a state-dependent manner in female prairie voles

Female prairie voles develop pair bonds faster than males^42^. We therefore tested distinct aspects of social behavior and pair bonding across timelines that are sensitized to each sex (Fig. 1a) and, hereafter, analyzed the results from females and males separately. First, after establishing a role for Oxtr in regulating naïve female responses to novel males, we examined the effects of Oxtr on the response of bonded females to familiar (partner) and novel (stranger) males. Pair bonding results in increased prosocial and affiliative (huddling) behavior with partners and a dramatic switch to agonistic (rejection) behavior directed towards strangers^5,11^. We tested how changes in bonding state affect behavioral and neural responses to a male partner. We compared female responses to the same male before and after pair bond formation to determine how bonding shapes partner-associated activity over time (Supplementary Fig. 5a, Day 0: Introduction versus Day 4: Reunion). We found that WT females exhibited reduced anogenital investigation of a partner during reunion compared to their first encounter (Supplementary Fig. 5b, c). *Oxtr*^*1−/−*^ females showed low levels of anogenital investigation regardless of bonding status, and WT and *Oxtr*^*1−/−*^ females did not differ in levels of investigation of a partner following bonding. Similarly, both WT and *Oxtr*^*1−/−*^ females spent more time in affiliative side-by-side contact with their familiar partner during reunion compared to the same male during the introduction (Supplementary Fig. 5d). We then examined activity in the NAc during interactions with the partner across time. Activity in the NAc of both WT and *Oxtr*^*1−/−*^ females was higher during non-anogenital investigations of familiar partners at the time of reunion than of novel partners during introduction (Supplementary Fig. 5e–g), suggesting that interactions with familiar partners elicit greater activity in the NAc after bonding. Furthermore, we found that, despite differences in anogenital sniff-related activity during the introduction, WT and *Oxtr*^*1−/−*^ females exhibited similar patterns of activity in the NAc following bonding, suggesting that females can, over time, develop WT-like responses towards their partners in the absence of Oxtr function (Supplementary Fig. 5h–q). This indicates that Oxtr is not required for the changes in activity in the NAc during the investigation of partners that result from pair bond formation.

To test how pair bonding shapes the response to a novel male, we compared females’ responses to a novel male before pair bond formation (future partner) to responses to a novel male (stranger) following pair bond formation (Day 0: Introduction versus Day 6: Stranger rejection). WT females exhibited reduced anogenital investigation of a novel male following bonding (stranger), compared to when they were first paired with a novel male (partner; Supplementary Fig. 5c). In contrast, *Oxtr*^*1−/−*^ females showed consistently low levels of chemosensory investigation over the course of pair bonding. Both WT and *Oxtr*^*1−/−*^ females did not differ in their neural responses to stranger males after bonding (Supplementary Fig. 5e, h, k). Thus, loss of Oxtr specifically affects activity in the NAc during females’ interactions with novel males early in bonding, and Oxtr is not required for bonding-associated changes in NAc activity during anogenital investigation of a partner or a novel stranger.

Mating accelerates pair bond formation in prairie voles^43,44^. To understand the effects of Oxtr on behavior and activity in the NAc during the early stages of bonding, we examined activity in females during the first 30 min of mating, which occurred 48 h after animals were first introduced to each other and after estrus was induced and synchronized across pairs. Loss of Oxtr did not affect females’ mating or social behaviors with the partner (Supplementary Fig. 6a–f). During mounting attempts, activity within the NAc gradually decreased below baseline, and this decrease was greater during successful mounting attempts in which the male partner was able to proceed to intromission compared to attempts after which intromission did not occur (Supplementary Fig. 6g–l). *Oxtr*^*1−/−*^ females showed higher levels of activity during side-by-side contact when compared to WT animals, while no such differences were observed during any other social interactions (Supplementary Fig. 6m–x). These results suggest that loss of Oxtr exerts more significant effects on activity in the NAc of female voles at the earliest stages of bonding and that its effects are highly dependent on the bonding state.

**Fig. 2:**
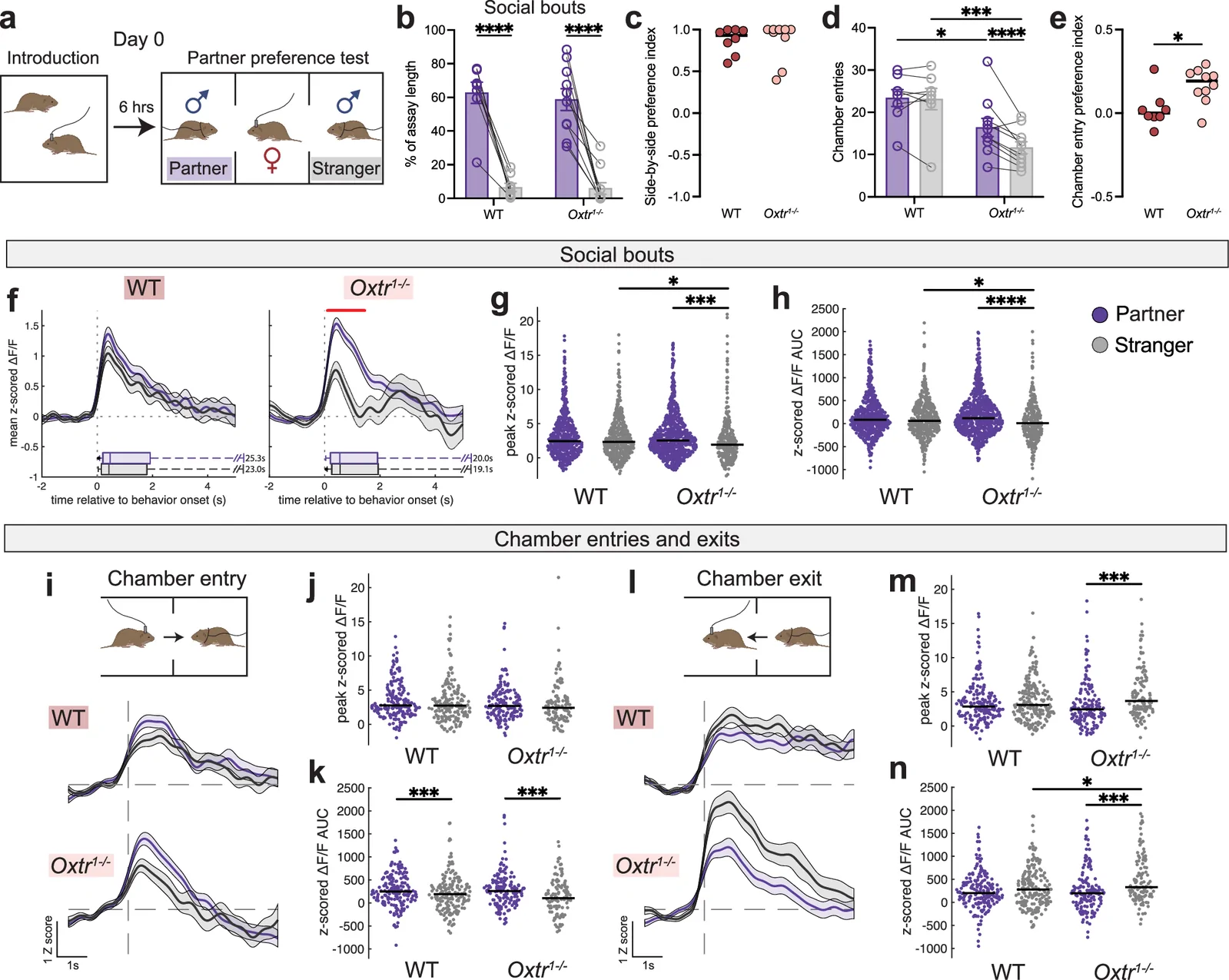
Loss of Oxtr disrupts female neural and behavioral responses to stranger males. **a** Timeline and schematic of the introduction and partner preference test (PPT) for females. **b** Percent of assay time spent engaged in social bouts with either the partner (purple) or the stranger (gray) (WT *n* = 8 voles, *Oxtr*^*1−/−*^ *n* = 10; two-sided 2-way repeated measures ANOVA, Main effect of Stimulus animal, F_1, 16_ = 109.4, *p* < 0.0001). **c** Side-by-side contact preference index scores. Preference index scores of 1 indicate exclusive side-by-side contact with the partner, and −1 with the stranger (WT *n* = 8 voles, *Oxtr*^*1−/−*^ *n* = 10, two-sided permutation test on Earth-Movers Distance [EMD], EMD = 0.079, *p* = 0.52). **d** Number of entries to the partner or stranger chambers (WT *n* = 8 voles, *Oxtr*^*1−/−*^ *n* = 10, generalized linear model [GLM] with two-parameter Poisson distribution, Z_1,16_ = −2.675, *p* = 0.0074, Sidak-corrected post hoc GLMs). **e** Chamber entry preference index scores (WT n = 8 voles, *Oxtr*^*1−/−*^ *n* = 10, two-sided permutation test on EMD, EMD = 0.14, *p* = 0.01). **f** Mean PETH of z-scored GCaMP6m ΔF/F by stimulus animal aligned to the onset of social bouts (WT_Partner_ *n* = 539 traces; WT_Stranger_ *n* = 444; *Oxtr*^*1−/−*^_Partner_ *n* = 732; *Oxtr*^*1−/−*^_Stranger_ *n* = 304). Data are presented as mean values ± SEM. Box plot shows the median (center line) of durations of the initiating behavior, interquartile range (box), and whiskers adjusted for skewed data (see “Methods”); outliers are plotted as individual points. The red line indicates time points at which the mean z-scored ΔF/F differs between partner and stranger-related activity in *Oxtr*^*1−/−*^ females. **g** Swa{Updating}rm plot of peak z-scored ΔF/F values (WT_Partner_ *n* = 539 traces; WT_Stranger_ *n* = 444; *Oxtr*^*1−/−*^_Partner_ *n* = 732; *Oxtr*^*1−/−*^_Stranger_ *n* = 304; LME, Genotype by Stimulus animal, F_1,2015_ = 5.53, *p* = 0.019, Sidak-corrected post hoc LMEs). **h** AUC values from z-scored ΔF/F traces (WT_Partner_ *n* = 539 traces; WT_Stranger_ *n* = 444; *Oxtr*^*1−/−*^_Partner_ *n* = 732; *Oxtr*^*1−/−*^_Stranger_ *n* = 304; LME, Genotype by Stimulus animal, F_1,2015_ = 7.22, *p* = 0.0073, Sidak-corrected post hoc LMEs). **i** Left, mean ΔF/F PETH aligned to entries to either the partner chamber or stranger chamber (WT_Partner_ n = 184 traces; WT_Stranger_ *n* = 182; *Oxtr*^*1−/−*^_Partner_ *n* = 160; *Oxtr*^*1−/−*^_Stranger_ *n* = 115). Data are presented as mean values ± SEM. **j** Peak z-scored ΔF/F values (WT_Partner_ *n* = 184 traces; WT_Stranger_ *n* = 182; *Oxtr*^*1−/−*^_Partner_ *n* = 160; *Oxtr*^*1−/−*^_Stranger_ *n* = 115; LME, Genotype by Stimulus animal, F_1,637_ = 0.094, *p* = 0.76). **k** AUC values from z-scored ΔF/F traces (WT_Partner_ *n* = 184 traces; WT_Stranger_ *n* = 182; *Oxtr*^*1−/−*^_Partner_ *n* = 160; *Oxtr*^*1−/−*^_Stranger_ *n* = 115; LME, main effect of Stimulus animal, F_1,637_ = 12.13, *p* = 0.00053). **l** Left, mean ΔF/F PETH aligned to exits from either the partner chamber or stranger chamber (WT_Partner_ *n* = 187 traces; WT_Stranger_ *n* = 195; *Oxtr*^*1−/−*^_Partner_ *n* = 150; *Oxtr*^*1−/−*^_Stranger_ *n* = 121). Data are presented as mean values ± SEM. **m** Peak z-scored ΔF/F values (WT_Partner_ *n* = 187 traces; WT_Stranger_ *n* = 195; *Oxtr*^*1−/−*^_Partner_ *n* = 150; *Oxtr*^*1−/−*^_Stranger_ *n* = 121; LME, Genotype by Stimulus animal, F_1,649_ = 7.87, *p* = 0.0052, Sidak-corrected post hoc LMEs). **n** AUC values from z-scored ΔF/F traces (WT_Partner_ *n* = 187 traces; WT_Stranger_ *n* = 195; *Oxtr*^*1−/−*^_Partner_ *n* = 150; *Oxtr*^*1−/−*^_Stranger_ *n* = 121; LME, Genotype by Stimulus animal, F_1,649_ = 4.80, *p* = 0.029, Sidak-corrected post hoc LMEs). Detailed statistics are presented in Extended Data File 1. *p < 0.05, **p < 0.01, ***p < 0.001, ****p < 0.0001. Vole images were created in BioRender. Manoli, D. (2026) https://BioRender.com/00l1i4c. Source data are provided as a Source Data file.

The selective preference for a partner over a stranger is a critical component of pair bonding^42,43^. Six hours of cohabitation with a novel male are sufficient for female prairie voles to form a pair bond. This is reflected in the display of partner preference, *i.e*., a preference to engage in social interaction and side-by-side contact with their partner rather than a novel, potential mate^11,42^. We therefore tested whether the loss of Oxtr impacts partner preference at the earliest point after which WT females display such behavior. We placed females in a 3-chamber arena six hours after initial introductions and allowed them to choose between interacting with their partner or a male stranger (Fig. 2a). We found that loss of Oxtr did not disrupt female displays of partner preference. All females, regardless of genotype, interacted significantly more with their partner than with the stranger (Fig. 2b and Supplementary Fig. 7a, b) and displayed a clear preference to engage in affiliative side-by-side contact with their partner (Fig. 2c). We then examined NAc neural responses during interactions with the partner. Both peak and AUC of activity were similar between WT and *Oxtr*^*1−/−*^ females (Fig. 2f–h, partner-related activity in purple). This contrasts with the differences observed during females’ first encounter with the same partner male, again suggesting that Oxtr function is not required for bonding-associated changes in neural responses to partners.

While we did not observe a difference in females’ interactions with their partner between WT and *Oxtr*^*1−/−*^ animals during tests of partner preference, we observed differences in both behavioral and neural responses to stranger males in *Oxtr*^*1−/−*^ females. In contrast to WT females, all of which showed at least two bouts of side-by-side contact with strangers, *Oxtr*^*1−/−*^ females avoided stranger males (Supplementary Fig. 7c). Furthermore, *Oxtr*^*1−/−*^ females were less likely to enter the stranger male’s chamber (Fig. 2d, e). Neural responses to stranger males correlated with these differences in behavior in *Oxtr*^*1−/−*^ females. Both peak and AUC of calcium activity in the NAc during interactions with the stranger male were significantly decreased in *Oxtr*^*1−/−*^ females when compared to WT females, as well as *Oxtr*^*1−/−*^ females’ interactions with partners (Fig. 2f–h and Supplementary Fig. 7d–l). Entry into the partner chamber elicited greater total activity (AUC) than entry into the stranger chamber, regardless of genotype (Fig. 2i); however, *Oxtr*^*1−/−*^ females exhibited greater NAc activity upon leaving the stranger chamber than WT females (Fig. 2j). Our findings suggest that loss of Oxtr influences prosocial behavior and associated neural activity in the NAc in a state-dependent (pre- versus post-bonding) and context-dependent (partner versus stranger) manner in females. At the earliest stages of pair bonding, loss of Oxtr function reduces prosocial behavior towards novel males and decreases associated neural activity in the NAc. However, increases in prosocial behavior, huddling behavior, and activity in the NAc during interactions with partners following pair bonding occur independently of Oxtr function. Importantly, however, after bonding, Oxtr function does promote interactions with strangers and associated activity in the NAc.

### Loss of Oxtr disrupts NAc neural signatures of partner preference in male prairie voles

**Fig. 3:**
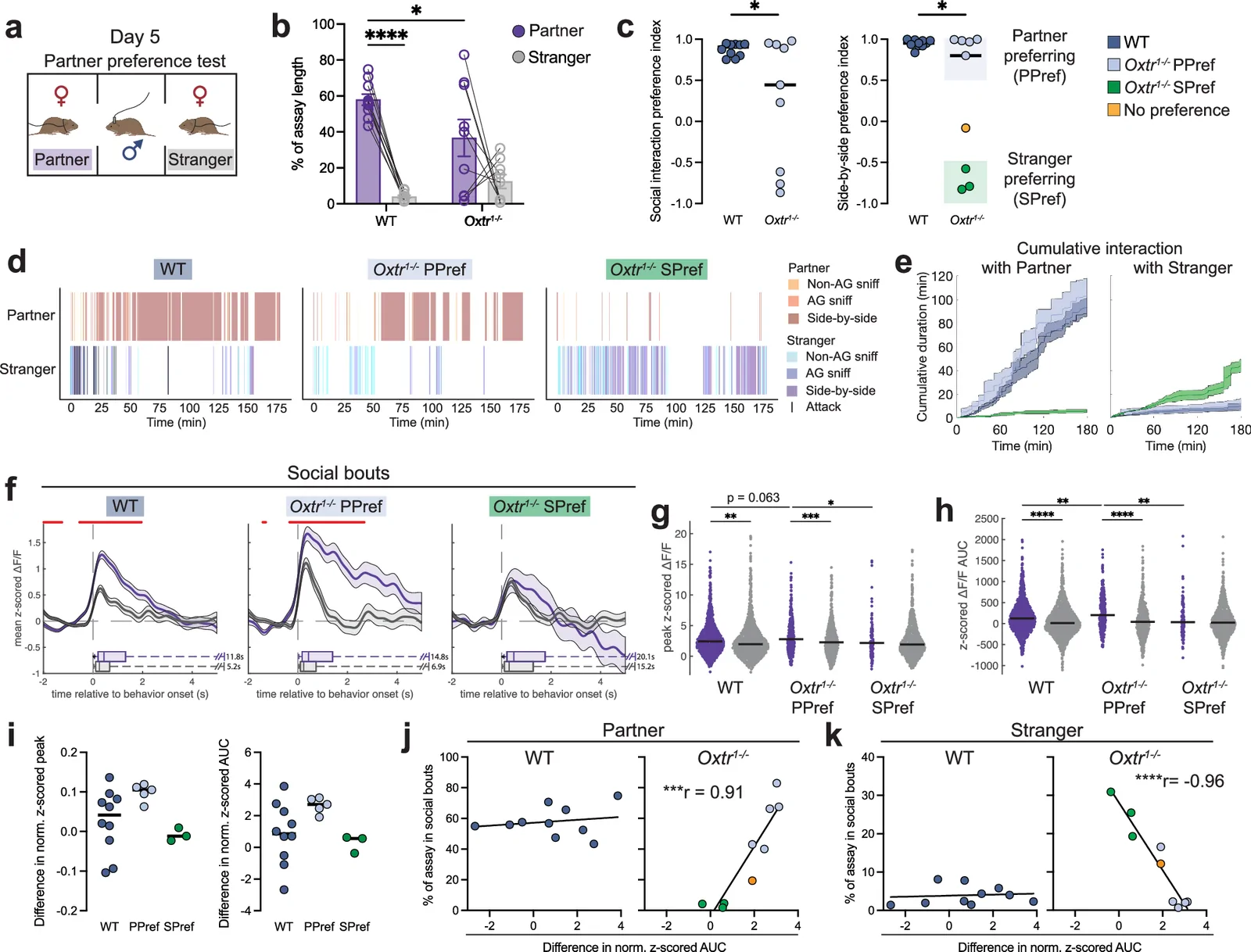
Loss of Oxtr disrupts NAc neural signatures of partner preference in male prairie voles. **a** Schematic of the PPT, conducted on day 5 in males. **b** Percent of assay time spent engaged in social bouts with either the partner (purple) or stranger (gray) (WT *n* = 10 voles, *Oxtr*^*1−/−*^ *n* = 9; two-sided 2-way repeated measures ANOVA, Genotype by Stimulus animal, F_1,17_ = 4.926, *p* = 0.040, Sidak-corrected post hoc comparisons). **c** Left, social interaction preference index scores (two-sided permutation test on Earth-Movers Distance [EMD], EMD = 0.64, *p* = 0.014). Right, side-by-side contact preference index scores (two-sided permutation test on EMD, EMD = 0.068, *p* = 0.019). Preference for partner or stranger was determined by whether side-by-side preference index scores were greater than 0.5 (partner-preferring, PPref) or less than −0.5 (stranger-preferring, SPref) (WT *n* = 10, *Oxtr*^*1−/−*^_PPref_ *n* = 5, *Oxtr*^*1−/−*^_SPref_ *n* = 3 voles). **d** Example behavior rasters from a WT, *Oxtr*^*1−/−*^_PPref_, and *Oxtr*^*1−/−*^_SPref_ male. **e** Mean (+/- s.e.m.) cumulative duration plots of social interaction with either the partner or stranger across the 3-h assay. **f** Mean ΔF/F PETH aligned to social bouts with either the partner or a stranger. The red line indicates time points at which mean z-scored ΔF/F differ between partner- and stranger-related activity (WT_Partner_ *n* = 932 traces and WT_Stranger_ *n* = 1193; *Oxtr*^*1−/−*^ PPref_Partner_ *n* = 296 and PPref_Stranger_ *n* = 500; *Oxtr*^*1−/−*^ SPref_Partner_ *n* = 111 and SPref_Stranger_ *n* = 821). Data are presented as mean values ± SEM. Box plots show the median (center line) of initiating behavior durations, interquartile range (box), and whiskers adjusted for skewed data (see “Methods”); outliers are plotted as individual points. **g** Peak ΔF/F values (WT_Partner_ *n* = 932 traces and WT_Stranger_ *n* = 1193; *Oxtr*^*1−/−*^ PPref_Partner_ *n* = 296 and PPref_Stranger_ *n* = 500; *Oxtr*^*1−/−*^ SPref_Partner_ *n* = 111 and SPref_Stranger_ *n* = 821; LME, Group by Stimulus animal, F_2,3847_ = 3.24, *p* = 0.039, Sidak-corrected post hoc LMEs). **h** AUC values (WT_Partner_ *n* = 932 traces and WT_Stranger_ *n* = 1193; *Oxtr*^*1−/−*^ PPref_Partner_ *n* = 296 and PPref_Stranger_ *n* = 500; *Oxtr*^*1−/−*^ SPref_Partner_ *n* = 111 and SPref_Stranger_ *n* = 821; LME, Group by Stimulus animal, F_2,3847_ = 4.18, *p* = 0.015, Sidak-corrected post hoc LMEs). **i** Per animal difference (partner - stranger) between partner-elicited and stranger-elicited peak ΔF/F (left, two-sided permutation test on F statistic, *p* = 0.064) and AUC (right) (WT *n* = 10 voles, *Oxtr*^*1−/−*^_PPref_ *n* = 5, *Oxtr*^*1−/−*^_SPref_ *n* = 3; two-sided permutation test on F statistic, *p* = 0.077). **j** Correlations of partner-stranger AUC difference and percent of time spent in social interactions with the partner (WT *r* = 0.19, *p* = 0.61; *Oxtr*^*1−/−*^ *r* = 0.91, *p* = 0.0007). **k** Correlations of partner-stranger AUC difference and percent of time spent in social interactions with the stranger (WT *r* = 0.10, *p* = 0.78; *Oxtr*^*1−/−*^ *r* = −0.96, *p* < 0.0001). Detailed statistics are present in Extended Data File 1. *p < 0.05, **p < 0.01, ***p < 0.001, ****p < 0.0001. Vole images were created in BioRender. Manoli, D. (2026) https://BioRender.com/00l1i4c. Source data are provided as a Source Data file.

We examined the effects of Oxtr loss on neural activity in the NAc associated with pair bond formation in males. During mating, WT and *Oxtr*^*1−/−*^ males did not differ in their mating or prosocial behavior, related NAc activity, or relationship of neural activity to subsequent behavior (Supplementary Fig. 8a–m). Compared to females, male prairie voles require longer periods of cohabitation and mating before they display robust preference for partners^11,42^. We therefore examined partner preference in males 5 days after introduction to WT females, a period of cohabitation that is sufficient for partner preference formation in WT males^11^ (Fig. 3a). In contrast to our observations in females, loss of Oxtr disrupted the display of partner preference in males even after 5 days of cohabitation (Fig. 3b, c). The difference in preference between populations of WT versus *Oxtr*^*1−/−*^ males was due to a subgroup of mutant males that strongly preferred interacting with a stranger female over their partner (Fig. 3c–e and Supplementary Fig. 9a, b). Fifty-six percent of *Oxtr*^*1−/−*^ males preferred to engage in side-by-side contact with the partner (index score >0.5, “partner-preferring”), while 33% spent little time with the partner in favor of the stranger (index score <−0.5, “stranger-preferring,” Fig. 3c). In contrast, all WT males exhibited robust preference for partners.

We then analyzed activity in the NAc to determine whether these behaviorally defined subpopulations of *Oxtr*^*1−/−*^ males also differed in their neural responses in the NAc during social interactions. All males showed increased activity in the NAc upon entering the partner’s chamber versus that of the stranger and, inversely, increased activity when exiting strangers’ versus partners’ chambers (Supplementary Fig. 9c–f). Thus, an approach towards partners (or departure from strangers) increases NAc activity in male prairie voles independent of Oxtr function^35^. In contrast to activity associated with such transitions, activity during direct social interactions differed between subgroups. In both WT and partner-preferring *Oxtr*^*1−/−*^ males, activity was significantly greater during social interactions with partners compared to social interactions with strangers (Fig. 3f–h, Supplementary Figs. 9g–p and 10a). Moreover, partner-preferring *Oxtr*^*1−/−*^ males showed even higher levels of activity in the NAc during social interactions with partners compared to WT males (Fig. 3h).

In contrast, stranger-preferring *Oxtr*^*1−/−*^ males showed minimal differences in activity in the NAc when comparing interactions with partners or strangers (Fig. 3f–i and Supplementary Fig. 10b). Levels of activity during interactions with partners were lower in stranger-preferring *Oxtr*^*1−/−*^ males when compared to partner-preferring *Oxtr*^*1−/−*^ males (Fig. 3f–h, partner in purple). Individual stranger-preferring *Oxtr*^*1−/−*^ males tended to have a smaller difference in mean partner- versus mean stranger-related activity in the NAc when compared to partner-preferring *Oxtr*^*1−/−*^ males (*p* = 0.064 for peak and *p* = 0.077 for AUC, Fig. 3i). For *Oxtr*^*1−/−*^ males, the difference in partner- versus stranger-related activity in the NAc strongly predicted the amount of social interaction displayed towards either female. *Oxtr*^*1−/−*^ males with less neural difference between partner and stranger interactions demonstrated less interaction with the partner and greater interaction with the stranger female (Fig. 3j, k). These relationships were not observed in WT males, which displayed robust preference for partners over strangers despite a large variation in the difference between partner- and stranger-associated activity in the NAc (Fig. 3i–k).

Thus, the formation of partner preference in males and the accompanying increase in activity in the NAc during interactions with partners versus strangers can occur independent of Oxtr function. However, loss of Oxtr unmasks a population of males that fail to display partner preference and show no difference in NAc activity during interactions with partners versus strangers. These observations suggest that Oxtr function is not necessary for the demonstration of partner preference or increased partner-related neural activity in the NAc in males but may facilitate the display of a partner preference and associated activity. Oxtr may therefore function during pair bond formation in males to reinforce both prosocial behaviors and increased activity in the NAc with partners or, alternatively, to suppress prosocial behavior during interactions with strangers.

### Loss of Oxtr regulates behavioral and neural trajectories of pair bonding in male prairie voles

Loss of Oxtr reveals distinct populations of males that strongly prefer either a partner or a stranger. We therefore analyzed the behavior of these populations at different stages of pair bonding to determine if the trajectories of social behaviors or patterns of activity in the NAc differed between these groups or between WT and *Oxtr*^*1−/−*^ males. We compared chemosensory investigation (anogenital and non-anogenital) between the first encounter with female partners (introduction) and either reunion with these partners or encounters with novel strangers during stranger rejection and found no differences between WT and *Oxtr*^*1−/−*^ males (Supplementary Fig. 11a–d). All males displayed increased side-by-side contact with the familiar partner upon reunion compared to the naïve partner during the introduction. Notably, stranger-preferring *Oxtr*^*1−/−*^ males did not display higher levels of agonistic behavior towards their partners (Supplementary Fig. 11e–h). Regardless of Oxtr function, activity in the NAc was greater during non-anogenital sniffs of female partners upon reunion compared to the introduction (Supplementary Fig. 11i–n). This suggests that, as with females, cohabitation enhances specific partner-associated activity in the NAc in males independent of Oxtr function.

**Fig. 4:**
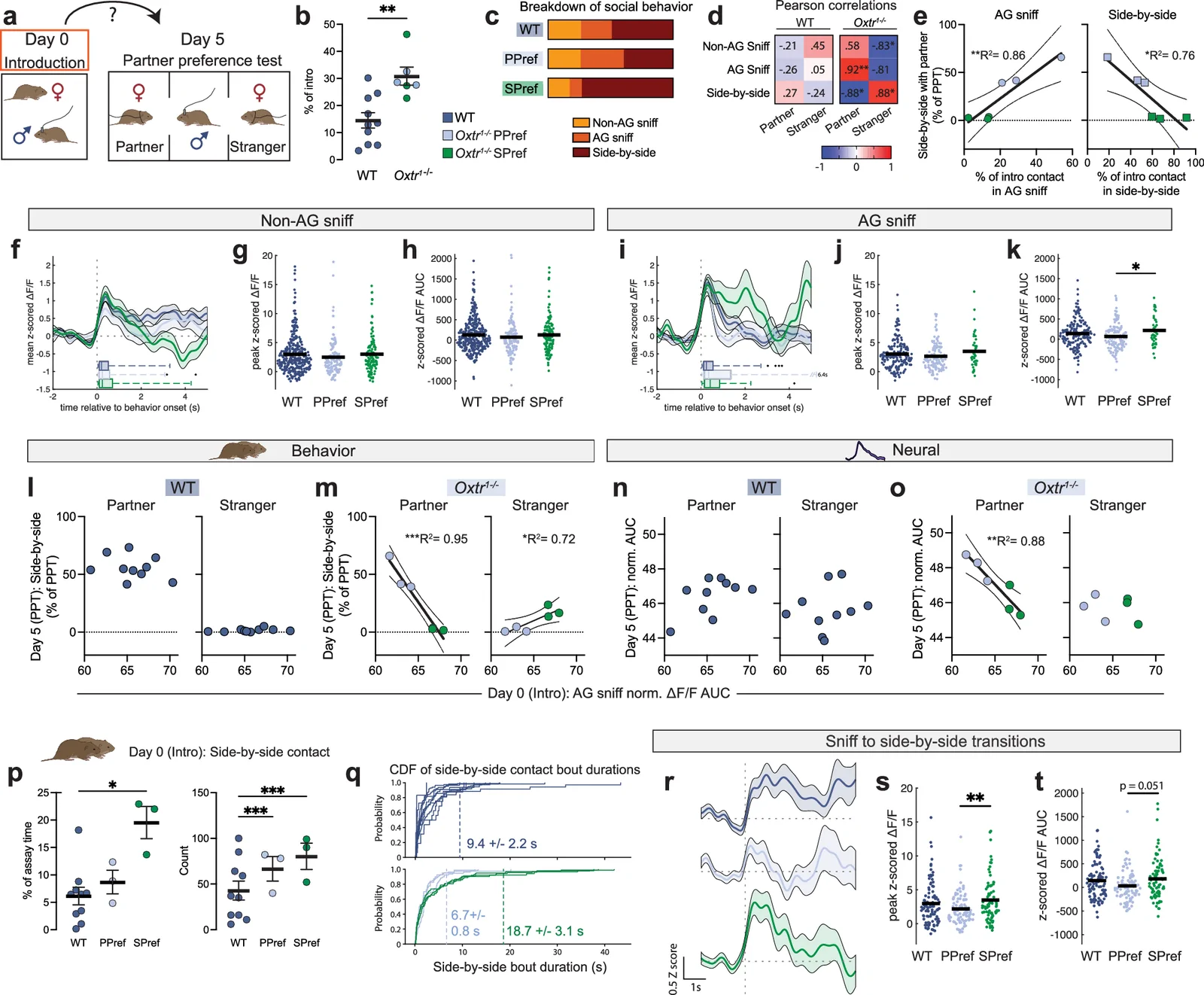
Loss of Oxtr regulates behavioral and neural trajectories of pair bonding in male prairie voles. **a** Examination of behavior and neural activity from Day 0 (introduction) in relation to metrics from Day 5 (PPT). **b** Percent of time engaged in social interaction with a newly partnered female during the introduction, plotting only animals for which data were successfully collected during both introduction and PPT. Individual animals are colored according to PPT behavior profile (WT *n* = 10 voles, *Oxtr*^*1−/−*^_PPref_ *n* = 3, *Oxtr*^*1−/−*^_SPref_ *n* = 3; two-sided t test, t(15) = 3.62, *p* = 0.0028). **c** Mean breakdown of social interaction during the introduction by type of social touch (two-sided 2-way repeated measures ANOVA with Geisser-Greenhouse correction, main effect of Behavior, F_1.167,15.16_ = 10.37, *p* = 0.0043). **d** Heat maps of Pearson correlations between introduction behavior (% of contact time) and PPT partner or stranger side-by-side contact (% of assay time). **e** Linear regression of introduction behavior to PPT behavior. X-axis: Percent of social touch during the introduction spent AG sniffing (left, *R*^2^ = 0.86; F_1,5_ = 29.55, *p* = 0.0029) or in side-by-side contact (right, *R*^2^ = 0.76; F_1,4_ = 12.93, *p* = 0.023). Y-axis: Percent of PPT time spent in side-by-side contact with the partner. **f** Mean NAc ΔF/F PETH aligned to non-AG sniffs during the introduction (WT *n* = 338 traces; *Oxtr*^*1−/−*^_PPref_ *n* = 138; *Oxtr*^*1−/−*^_SPref_ *n* = 123). Data are presented as mean values ± EM. Box plots show the median (center line) of initiating behavior durations, interquartile range (box), and whiskers adjusted for skewed data (see “Methods”); outliers are plotted as individual points. **g** Peak z-scored ΔF/F values (WT *n* = 338 traces; *Oxtr*^*1−/−*^_PPref_ *n* = 138; *Oxtr*^*1−/−*^_SPref_ *n* = 123; LME, F_2,596_ = 1.16, *p* = 0.31). **h** AUC values from z-scored ΔF/F traces (WT *n* = 338 traces; *Oxtr*^*1−/−*^_PPref_ *n* = 138; *Oxtr*^*1−/−*^_SPref_ *n* = 123; LME, F_2,596_ = 1.35, *p* = 0.26). **i** Mean ΔF/F PETH aligned to AG sniffs during the introduction (WT *n* = 187 traces; *Oxtr*^*1−/−*^_PPref_ *n* = 129; *Oxtr*^*1−/−*^_SPref_ *n* = 42). Data are presented as mean values ± SEM. Box plots show the median (center line) of initiating behavior durations, interquartile range (box), and whiskers adjusted for skewed data (see “Methods”); outliers are plotted as individual points. **j** Peak z-scored ΔF/F values (WT *n* = 187 traces; *Oxtr*^*1−/−*^_PPref_ *n* = 129; *Oxtr*^*1−/−*^_SPref_ *n* = 42; LME, F_2,355_ = 2.03, *p* = 0.13). **k** AUC values from z-scored ΔF/F traces (WT *n* = 187 traces; *Oxtr*^*1−/−*^_PPref_ *n* = 129; *Oxtr*^*1−/−*^_SPref_ *n* = 42; LME, F_2,355_ = 3.40, *p* = 0.035, Sidak-corrected post hoc LMEs). **l–o** Linear regressions comparing AG sniff-related NAc activity and PPT behavior or neural data. X-axis: normalized (norm.) AUC at the onset of AG sniff bouts during the introduction, averaged by animal. Y-axis: PPT side-by-side contact (l-m; WT partner, *R*^2^ = 0.074; F_1,8_ = 0.64, *p* = 0.45; WT stranger, *R*^2^ = 0.16; F_1,8_ = 1.49, *p* = 0.26; *Oxtr*^*1−/−*^ partner, *R*^2^ = 0.95; F_1,4_ = 83.04, *p* = 0.0008; *Oxtr*^*1−/−*^ stranger, *R*^2^ = 0.72; F_1,4_ = 10.07, *p* = 0.034) or PPT normalized AUC surrounding social bouts, averaged by animal (n-o; WT partner, *R*^2^ = 0.32; F_1,8_ = 3.73, *p* = 0.090; WT stranger, *R*^2^ = 0.040; F_1,8_ = 0.33, *p* = 0.58; *Oxtr*^*1−/−*^ partner, *R*^2^ = 0.88; F_1,4_ = 28.39, *p* = 0.006; *Oxtr*^*1−/−*^ stranger, *R*^2^ = 0.09; F_1,4_ = 0.40, *p* = 0.56). **p** Left, percent of introduction time spent in side-by-side contact (two-sided permutation test on F statistic, *p* = 0.0062, Sidak-corrected post hoc permutation tests on t statistic). Right, number of side-by-side contact events during the introduction WT *n* = 10 voles, *Oxtr*^*1−/−*^_PPref_ *n* = 3, *Oxtr*^*1−/−*^_SPref_ *n* = 3; GLM with 2-parameter Poisson distribution, F_2,13_ = 6.99, *p* = 0.030, Sidak-corrected post hoc GLMs). **q** Cumulative distribution functions (CDF) for side-by-side contact bout durations per animal. Dotted lines show the mean (+/- s.e.m.) of the 95th percentile values from each group. **r** Mean PETH aligned to transitions from sniffing to side-by-side contact, centered at the onset of the sniff (no filtering for behavior in the 2 s prior to sniff onset; WT *n* = 97 traces; *Oxtr*^*1−/−*^_PPref_ *n* = 98, *Oxtr*^*1−/−*^_SPref_ *n* = 82). Data are presented as mean values ± SEM. **s** Peak z-scored ΔF/F values (WT *n* = 97 traces; *Oxtr*^*1−/−*^_PPref_ *n* = 98, *Oxtr*^*1−/−*^_SPref_ *n* = 82; LME, F_2,274_ = 4.34, *p* = 0.014, Sidak-corrected post hoc LMEs). **t** AUC values from z-scored ΔF/F traces (WT *n* = 97 traces; *Oxtr*^*1−/−*^_PPref_ *n* = 98, *Oxtr*^*1−/−*^_SPref_ *n* = 82; LME, F_2,274_ = 3.15, *p* = 0.045, Sidak-corrected post hoc LMEs). Detailed statistics are presented in Extended Data File 1. *p < 0.05, **p < 0.01, ***p < 0.001, ****p < 0.0001. Vole images were created in BioRender. Manoli, D. (2026) https://BioRender.com/00l1i4c. Source data are provided as a Source Data file.

Given the emergence of distinct populations of *Oxtr*^*1−/−*^ males that strongly prefer partners or strangers, we tested whether these populations might be distinguishable at the initial stages of pair bonding. We examined male behavior and neural activity during the first interactions with a partner to determine if patterns of behavior or activity in the NAc could predict the future preference for partners or strangers (Fig. 4a). Naïve *Oxtr*^*1−/−*^ males engage in more prosocial interactions when first introduced to a female (Fig. 4b). We found no difference in the total amount of social behaviors when we compared partner- versus stranger-preferring *Oxtr*^*1−/−*^ males (Fig. 4b and Supplementary Fig. 11o–q). However, examining the specific patterns of behavior during social interactions revealed significant differences between these populations. Stranger-preferring *Oxtr*^*1−/−*^ males tended to spend more of their interaction time engaged in highly affiliative side-by-side contact compared to partner-preferring *Oxtr*^*1−/−*^ males, who spent more time engaged in anogenital investigation (Fig. 4c and Supplementary Fig. 11p). Moreover, the amount of time *Oxtr*^*1−/−*^ males engaged in anogenital investigation and side-by-side contact during initial interactions with a partner significantly correlated, in opposing directions, with levels of partner- and stranger-directed side-by-side contact during the partner preference test (Fig. 4d and Supplementary Fig. 11r, s). Thus, behavior exhibited by *Oxtr*^*1−/−*^ males during the first 30 min of interaction with a female can predict patterns of partner preference 5 days later (Fig. 4e and Supplementary Fig. 11t–x), indicating that the two subpopulations are immediately identifiable based on their behavior.

Stranger-preferring *Oxtr*^*1−/−*^ males differ from partner-preferring *Oxtr*^*1−/−*^ and WT males in both their amount of anogenital investigation and side-by-side contact (Fig. 4c). We examined whether these behavioral differences were associated with differences in activity in the NAc during social interactions. During the earliest interactions with a partner, activity in the NAc associated with anogenital investigation was greater in *Oxtr*^*1−/−*^ males that went on to prefer strangers compared to those that went on to prefer partners (Fig. 4i–k). In contrast, activity in the NAc associated with non-anogenital investigation was similar across all males. Consistent with our behavioral observations, the mean neural activity following an anogenital sniff during initial interactions with a female strongly correlated with affiliative side-by-side behavior displayed towards partners *and* strangers 5 days later in individual *Oxtr*^*1−/−*^, but not WT, males. (Fig. 4l, m). Neural activity in the NAc of *Oxtr*^*1−/−*^ males was strongly inversely correlated between early and late interactions with the same female. Specifically, partner-preferring *Oxtr*^*1−/−*^ males showed lower levels of activity following anogenital investigations during the introduction and higher levels of activity during social interactions with partners 5 days later. In contrast, stranger-preferring *Oxtr*^*1−/−*^ males displayed the opposite pattern of activity (Fig. 4n). We observed no correlation between early and future partner-related activity in WT males (Fig. 4n, o). Thus, in the absence of the Oxtr function, early anogenital chemosensory investigation of novel females and associated neural activity in the NAc may rapidly influence how males bond during cohabitation and may even predict future preference.

In rodents, social interactions are typically initiated by chemosensory investigations that inform animals of each other’s identity (*e.g*., species, sex, health, reproductive status, etc.)^45,46^. We analyzed the dynamics of and the relationship between prosocial side-by-side contact, activity within the NAc, and future preference for partners or strangers. The majority (73.8%) of side-by-side events during introductions were preceded by sniff investigations. We observed that stranger-preferring *Oxtr*^*1−/−*^ males spent significantly more time in side-by-side contact and in longer bouts of this contact with partner females during initial interactions than both WT males and partner-preferring *Oxtr*^*1−/−*^ males (Fig. 4p, q). We examined NAc calcium activity at the transition from sniffs to bouts of side-by-side contact. As with anogenital investigations alone, the transition from such sniffs to side-by-side contact during initial interactions was associated with higher peak activity in the NAc in future stranger-preferring *Oxtr*^*1−/−*^ males (Fig. 4r–t). Similarly, mean NAc neural responses at the onset of such transitions were strongly correlated with patterns of preference for partners or strangers 5 days later in *Oxtr*^*1−/−*^, but not WT, males (Supplementary Fig. 11y–bb).

The dynamics of chemoinvestigation and prosocial behaviors and the associated neural activity in the NAc during the first interactions of *Oxtr*^*1−/−*^ males with a novel female can therefore predict future preference for and neural responses to partners and strangers. In the absence of Oxtr, higher levels of activity in the NAc following anogenital investigations correlate with increased side-by-side contact during males’ initial interactions with a partner female, but a preference for a stranger female in the future. In contrast, lower levels of NAc activity upon introduction correlate with increased chemoinvestigation and less side-by-side contact, but a robust later preference for these females. Thus, loss of Oxtr modulates neural responses to chemosensory information from the first interaction between a male vole and his female partner, suggesting that one role of Oxtr is to influence behavior trajectories towards a common pair-bonded outcome. Loss of Oxtr unmasks a population of males that show a robust stranger preference, even after prolonged cohabitation with a partner. These findings suggest that Oxtr function works to stabilize a range of sensory responses and social behaviors towards common and less-varied patterns of interactions to promote social monogamy in male prairie voles.

## Discussion

Enduring attachments are comprised of nuanced and finely tuned behaviors under complex neural and hormonal control. To examine neural activity associated with the formation of long-term social attachments, we implemented fiber photometry in the NAc, a node in the circuitry that mediates pair bonding in socially monogamous prairie voles that is enriched for oxytocin binding in monogamous versus promiscuous vole species^31^. We examined activity in this region across pair bond formation and related attachment behaviors in male and female wild-type animals and those lacking Oxtr. Activity in the NAc during interactions with partners diverges from interactions with strangers over the course of pair bonding. Loss of Oxtr has opposing effects on specific pair-bond-related social behaviors and associated NAc activity between males and females. Strikingly, in *Oxtr*^*1−/−*^ males, behavior and activity in the NAc during the first interactions with potential mates strongly predict future preference for partners or strangers 5 days later. Critically, the interrogations of this study would not have been possible without the integration of in vivo calcium recordings, molecular genetics, and ethological approaches^47,48^.

**Fig. 5:**
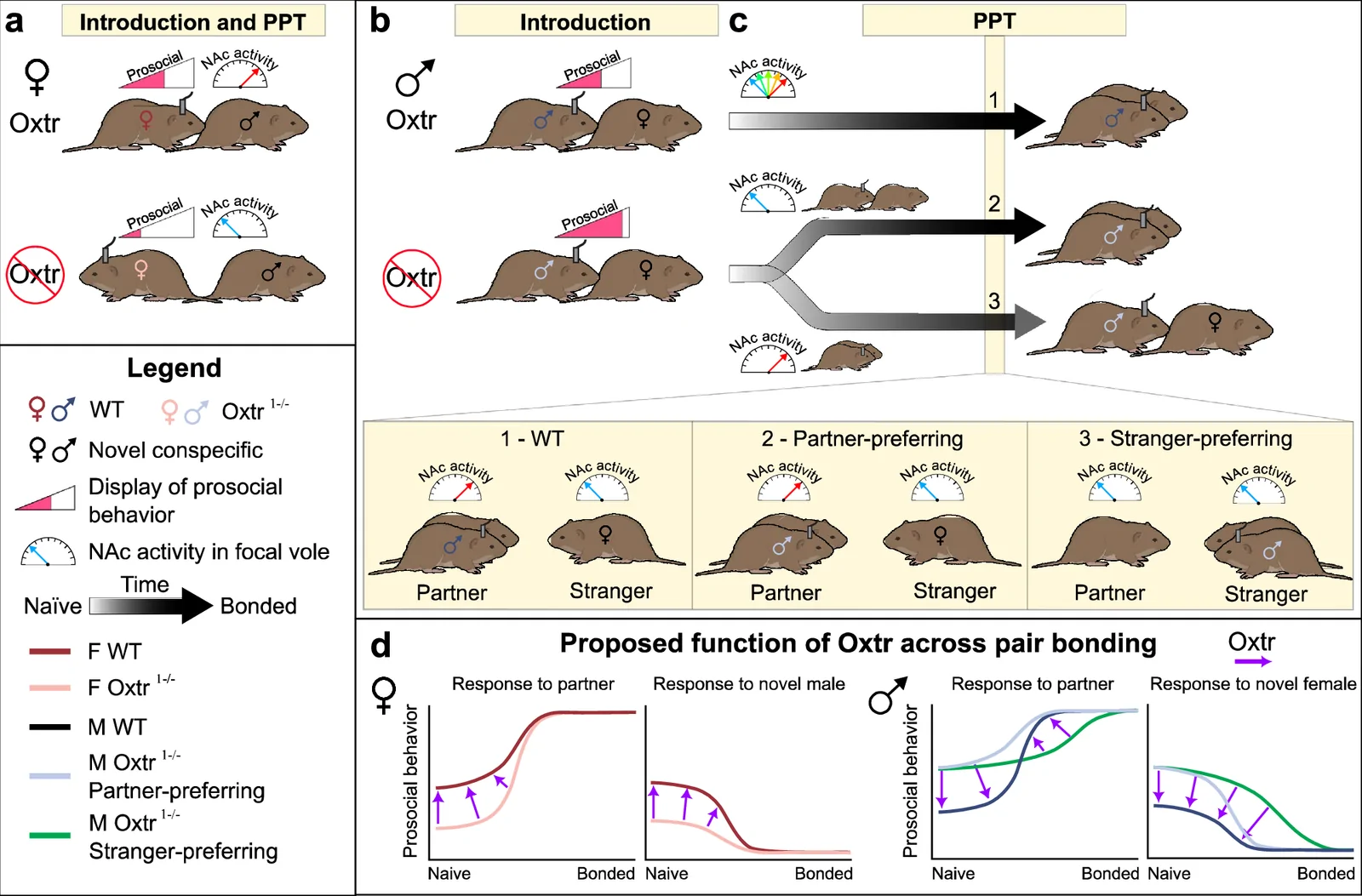
Model for proposed role of Oxtr function in pair bonding and associated activity in the NAc. **a** In female prairie voles, loss of Oxtr reduces prosocial interaction directed towards novel males, which is accompanied by reduced neural activity in the nucleus accumbens (NAc). **b** In male prairie voles, loss of Oxtr increases prosocial interaction directed towards novel females during early interactions. **c** In males, lower neural activity levels in the NAc during early interactions are associated with typical partner preference formation, while higher activity levels are associated with decreased chemoinvestigation upon first encounter and a preference for strangers when given a choice 5 days later. **d** Proposed role of Oxtr function in female and male prairie voles. Vole images were created in BioRender. Manoli, D. (2026) https://BioRender.com/00l1i4c.

Loss of Oxtr has different, and even opposing, effects on prosocial behaviors and activity in the NAc between males and females. During initial encounters with a novel male, naïve Oxtr-null females show lower levels of all types of investigative and affiliative social interaction, which are accompanied by reduced neural activity during chemosensory investigation of males, suggesting that Oxtr function promotes prosocial behaviors with novel, potential mates^49^ (Fig. 5a). Social responses to novel males in this sexually naïve state may represent a distinct behavioral module from partner preference that influences the initial acquisition of a mate^5,11,50–52^.

Following cohabitation sufficient for partner preference formation, females huddle with partners significantly more than strangers. Consistent with our prior work, we find that this display of partner preference, a component of pair bonding, does not require Oxtr function^10,11^. However, loss of Oxtr continues to impact female behavior towards novel males early in pair bonding: despite a distinct preference to huddle with partners, wild-type females enter partners’ and strangers’ chambers equally. In contrast, *Oxtr*^*1−/−*^ females enter strangers’ chambers significantly less often than partners’, and few venture close enough for side-by-side contact. Moreover, NAc activity during interactions with strangers, but not partners, is altered in these mutants. Together, these findings are consistent with a role for Oxtr in promoting females’ interactions with novel males, but not a preference for or response to partners following bonding.

The effects of Oxtr loss in males differ from those in females. Loss of Oxtr function decreases aggression and *promotes* males’ prosocial interactions with novel females during initial encounters, suggesting that Oxtr function dampens prosocial drive in naïve male prairie voles (Fig. 5b). Following days of cohabitation with partners, mutant males display two distinct patterns of partner preference—preference for familiar “partners” and preference for novel “strangers.” Thus, in males, Oxtr function reduces the variability of early prosocial behavior towards partners and neural activity in the NAc of prairie voles to promote pair bond formation (Fig. 5c).

Notably, whether an *Oxtr*^*1−/−*^ male will prefer the partner female can be predicted by chemoinvestigation and related activity in the NAc during his first interactions with that female “partner.” This correlation merits cautious interpretation, given limited group sizes; larger cohorts will allow a more comprehensive evaluation of these patterns. The predictive relationship between initial social interactions and the fidelity of future bonds in the absence of Oxtr suggests that, in males, Oxtr signaling functions to reinforce attachment between mates or even to override other pathways that drive social behaviors associated with a lower propensity for pair bond formation^53–55^.

Loss of Oxtr has sexually dimorphic effects on behavior and neural activity in the NAc, pointing to differences in how Oxtr has evolved to control social behaviors in male and female prairie voles (Fig. 5d). In females, Oxtr may facilitate attachment by promoting prosocial interactions leading to initial mate acceptance and pair bonding. In males, Oxtr may reduce promiscuity by decorrelating variation during initial chemoinvestigation and social interactions between partners from the neural pathways driving pair bonding. This suggests that oxytocin acts on ancestral, sexually dimorphic neural circuits to reinforce behaviors that facilitate enduring attachments between mates^4,56,57^. Oxytocin neuromodulation in prairie voles may influence these circuits to generate more monomorphic neural function and, thus, more synchronized reciprocal patterns of behavior to promote long-term attachment between mates^11,53–55,58–63^.

How the loss of oxytocin neuromodulation impacts neural activity in the NAc to drive the differences we observed between wild-type and Oxtr-null animals remains to be determined. Such differences in neural activity may be driven by specific neuronal populations within this region^16,29,64^. Here, we recorded population-level activity from the NAc, and future experiments with cell-type-specific targeting are needed to determine how subpopulations contribute to the different patterns of activity we observed. Differences in activity within the NAc may also arise from intrinsic properties of neurons within this region^65–80^. We recently demonstrated that loss of Oxtr unmasks sex differences in gene expression in the NAc that are not found in wild-type animals^11^. Such transcriptional differences may influence structure and/or neuronal function to contribute to the differential processing of social chemosensory cues^65–71^. Alternatively, or in parallel, such sex differences in the NAc may arise from Oxtr-regulated inputs to this region that differ between males and females^23,24,72–75,81–83^, such that loss of Oxtr alters upstream afferent activity or integrative processing within the NAc. Consistent with this model, circuits that differ between the sexes influence the function of less dimorphic brain regions to generate sex-typical neural activity and behavior across species^84–86^.

Constitutive loss of Oxtr function may also have broader ramifications for the circuits and physiology that shape behavior. For example, the behavioral changes we observed in *Oxtr*^*1−/−*^ prairie voles may be driven by changes to neural activity outside of the NAc that, for example, alter sensory processing or reduce social motivation or salience^38–41,49,53,59,73,81–83^. Given the differences we observed in chemosensory-related neural activity in the NAc, evaluating changes to sensory processing in *Oxtr*^*1−/−*^ mutants may be a fruitful avenue of research. Whether or not loss of Oxtr signaling induces compensatory mechanisms via alternative neuromodulatory pathways or circuit mechanisms is also unknown. No evidence suggests that vasopressin signaling via V1aR is upregulated in Oxtr mutants, though loss of Oxtr may alter other neuromodulatory pathways in a compensatory manner in the NAc and other brain regions^10,11^. Constitutive loss of Oxtr function may also influence the development of neural circuits that regulate social and attachment behaviors. For example, levels of oxytocin and vasopressin expression are significantly reduced in regions of the paraventricular nucleus of the hypothalamus in prairie voles lacking Oxtr^11^. Further work is necessary to elucidate the mechanisms underlying changes in activity in the NAc of naive *Oxtr*^*1−/−*^ prairie voles and to compare these mechanisms in both males and females to address whether such changes are controlled by shared or distinct neural mechanisms in the sexes. Regardless, our work demonstrates that Oxtr function is genetically not required for the development of a pair bond in females or males; however, its loss regulates neural responses to chemosensory-related investigation and global prosocial behavior during the early stages of pair bond formation that may critically influence future fidelity and promiscuity.

The relationship between chemosensory-related activity in the NAc and prosocial behavior differs between the sexes. *Oxtr*^*1−/−*^ females exhibited decreased prosocial behavior and neural activity specifically related to chemoinvestigation. In *Oxtr*^*1−/−*^ males, increases in such activity were negatively correlated with the future display of partner preference. Thus, it is possible that chemosensory-associated activity in the NAc directly regulates prosocial behavior that promotes the development of partner preference. However, no differences were observed between wild-type and *Oxtr*^*1−/−*^ males’ neural responses to any social interaction during initial encounters, despite elevated amounts of prosocial behavior, suggesting that any potential causal relationship between population-level or cell-type specific neural activity in the NAc and specific social or bonding-related behaviors may be nuanced. Resolving this question will require real-time and cell-type-specific manipulations and circuit-level analyses.

Pharmacological studies in the prairie vole suggest that oxytocin signaling is critical for the formation of pair bonds^7–9,18^; however, our recent studies and our findings here indicate that pair bonds can develop in the absence of Oxtr function^10^. This suggests that pair bonding is a robust behavior that is not solely driven by oxytocin signaling. Rather, our work points to a role for oxytocin signaling in promoting the emergence of partner preference early in pair bond formation. This model reconciles pharmacological and Oxtr knockout experiments: while Oxtr is not strictly necessary for the formation of partner preference, signaling via this pathway may accelerate its development. By promoting acceptance of a mate in females and reducing promiscuity in males, oxytocin may critically facilitate social attachment in the ethological context of the prairie vole, in which mates become scarce during periods of low population density^50,63,87,88^. Moreover, our findings provide evidence that the loss of Oxtr shapes NAc neural responses to social interactions during early, but not late, time points in pair bonding, again indicating that Oxtr signaling is not required for the neural plasticity associated with bond formation. Loss of Oxtr reveals relationships between neural activity and behavior, paving the way for future work exploring what pair-bond-related behaviors are directly regulated by activity in the NAc and how chemosensory-related signaling facilitates pair bond formation.

By observing a wide range of social interactions across pair bonding, we investigated how modulatory mechanisms that have evolved to facilitate pair bonding influence both the behavior and neural activity that underlie long-term social attachments^10,11,86^. The effects of Oxtr signaling vary across time and sex, supporting a model in which other neuromodulatory pathways intersect with Oxtr function during bonding and attachment to influence prosocial and agonistic behaviors in a state-dependent manner. Extending these circuit and behavioral investigations to other brain regions will enable us to determine how Oxtr and other signaling pathways influence the activity of specific neural populations to control distinct modules of attachment, shedding light on the evolutionary processes driving social monogamy and the complex patterns of reciprocal interactions that constitute enduring relationships.

## Methods

### Animals

All animal care and procedures were approved by the University of California, San Francisco, Institutional Animal Care and Use Committee. A total of 76 adult (55–78 days at the start of behavior assays), sexually naive prairie voles (*Microtus ochrogaster*) were used in this study. Of these, 31 animals were rejected for experiments or analyses due to death (*n* = 4), poor signal quality (*n* = 9), head cap loss (*n* = 1), ataxia (*n* = 1), excessive aggression towards the partner (*n* = 1), mistargeting of the fiber (*n* = 9), or incorrect genotyping (*n* = 6). We used both male and female voles, with sex determined by the presence or absence of testes at weaning. Voles were bred in our laboratory from a population that originated from systematic outbreeding of a wild-caught stock captured near Champaign, IL, and housed at our facilities at the University of California, San Francisco. *Oxtr*^*1−/−*^ voles were derived from a line that we previously generated^10^, and wild-type (WT) voles were obtained from the same outcrossed background line. Breeding voles were maintained in large, plastic cages (10½” W x 19” L x 8” H, Ancare, R20 Rat/Guinea Pig caging) on Paperchip bedding (Shepherd Specialty Papers). Weaned voles were maintained at our breeding facility in clear plastic cages (45 × 25 × 15 cm, Innovive, Innocage IVC Rat Caging) on Paperchip bedding until they were transferred to our lab housing facility, at which point they were transferred to Sani-Chips woodchip bedding (P.J. Murphy—Forest Products Corp.). Voles were weaned at 21–25 days into group-housed cages, with 2–6 total same-sex siblings or similarly aged weanlings in a cage. Group-housed cages were given 2 cotton nestlets and a large PVC elbow tube. Voles had *ad libitum* access to food and water. When animals were paired, voles were housed in 30.80 × 30.80 × 18.72 cm cages (Thoren, Maxi-Miser Model #4) on Sani-Chips bedding and provided with 2 cotton nestlets and 2 small plastic tubes. All animals were kept on a 14:10 light-dark cycle.

### Genotyping

At the time of weaning, we collected a small tail sample from each animal and digested the tissue in lysis buffer with proteinase K (Sigma-Aldrich, St. Louis, MO). We conducted a polymerase chain reaction for the Oxtr gene using the following primers: Forward ACTGGAGCTTCGAGTTGGAC; Reverse ATGCCCACCACTTGCAAGTA. The resulting product was digested using XcmI enzyme (New England Biolabs, Ipswich, MA) and run on a 2% agarose gel. A second tail sample was collected after an animal concluded all assays, and genotyping was repeated for confirmation. Animals whose post-experiment samples did not match the original genotyping were excluded from all analyses.

### Surgery

Voles aged P29-55 were anesthetized with isoflurane and administered bupivacaine subcutaneously at the incision site. Using a stereotaxic frame (Kopf Instruments, Tujunga, CA), a craniotomy was made +1.7 mm anterior and +1.2 mm lateral relative to bregma, and a 33-gauge cannula was lowered to −5.1 mm relative to bregma. We injected a 5:1 mixture of AAV8-Syn-GCaMP6m-WPRE (1 × 10^13^ GC/mL, Vigene Biosciences, Inc., Rockville, MD) and AAV8-CAG-tdTomato (5 × 10^12^ GC/mL, Dr. Ed Boyden Lab, UNC Vector Core). We infused 0.7–1 µL of virus at a rate of 0.1 µL/min via an automated injection system (Genie Touch, Kent Scientific Corporation, Torrington, CT). The cannula was left in place for ten minutes to allow for viral diffusion, then was slowly removed to minimize viral infection along the needle tract. Following viral injection, 2 microscrews were implanted into the skull to provide additional stability for the dental cement. A fiber optic cannula (400 μm-diameter silica core, 0.48 NA, 6.5 mm length with flat tip) with a metal ferrule (1.25 mm base dimension, Doric Lenses, Inc., Québec, Canada) was implanted 0.05 mm above the injection site and secured to the skull with Metabond acrylic cement. Additionally, we created a well with which to hold a silicone elastomer that would provide extra stability for the fiber optic patch cable. A small hole was drilled into the cap of a microcentrifuge tube. The cap was placed upside down over the implant with the fiber ferrule protruding through the hole. The cap was secured to the Metabond with Ortho-Jet acrylic resin. Animals received postoperative buprenorphine and recovered for two to three weeks prior to testing. After three weeks, we checked each animal’s signal quality; animals with fluctuations in the 488 nm channel <5% of baseline (*i.e*., the signal output while the animals were still) and/or insufficient signal output in the 581 nm channel (< 10% of background fluorescence) were excluded from further study.

### Fiber photometry

Photometry experiments were conducted with an RZ5P Base Processor and Synapse software (Tucker-Davis Technologies, Alachua, FL). The RZ5P modulated a two-channel LED driver (Doric Lenses), controlling two connectorized LEDs (465 nm and 560 nm, Doric Lenses). The LEDs were coupled to a two-color filter cube system (5-port Fluorescence Mini Cube, Doric Lenses), allowing transmission of 465–480 nm light and 555-570 nm light for excitation of GCaMP and tdTomato, respectively, and capture of 500–540 nm and 580–680 nm light for monitoring brain fluorescence. Light was transmitted to a 1 × 1 fiber-optic rotary joint (Doric Lenses), which coupled to a patch cable (400 μm-diameter silica core, 0.48 NA, with 1.1 mm hytrel protective jacket, Doric Lenses) connected to the animal’s fiber optic cannula with a bronze or zirconia mating sleeve. Emission was filtered by the mini cube and captured by two 2151 Femtowatt Photoreceivers (Newport Corporation, Irvine, CA). The RZ5P recorded raw broadband photoreceiver traces. A USB camera was mounted above the cage and recorded behavior at 20 frames per second. Both photometry and camera recordings were controlled by the Tucker-Davis Technologies Synapse software, with camera frames and photometry samplings time-stamped for later synchronization.

### Behavior

Photometry procedures and habituation: All procedures were carried out during the light cycle. Introductions occurred between 08H00 and 12H00, and all other assays occurred between 12H00 and 20H00. To habituate animals to fiber tethering prior to testing, implanted voles were connected to a patch cable, placed in a clean cage, and given 30 min to habituate to fiber tethering at least 24 h before social behavior tests began. On the day of an assay, voles were transferred from the housing room to the procedure room at least half an hour prior to testing. Voles were briefly restrained by the experimenter, and a patch cable was connected to the fiber optic implant. The cable was additionally secured to the cannula via a silicone elastomer (Ecoflex, Smooth-On, Macungie, PA). During all assays, all tubes, food hoppers, and water bottles were removed from the cage. An experimenter remained in the room away from the cage to monitor signal quality, fiber tangling or damage, and aggression. If tangling of the fiber occurred, or if voles chewed the fiber, the experimenter intervened to untangle the fiber or discourage the vole with a loud snap of the fingers. We terminated an assay a) if excessive chewing continued, b) if the fiber was irreparably damaged, or c) if 3 bouts of highly aggressive tussling occurred.

#### Introductions

After attaching the patch cable, the vole was placed in a clean cage and given at least 5 min to habituate to fiber placement. An age-matched, opposite-sex prairie vole was then introduced to the cage, and behavior was recorded for 30 min. Only 3 animals displayed sexual behavior during the introduction (mounting without intromission in 1 WT male [1 mount], 1 *Oxtr*^*1−/−*^ male [1 mount], and 1 WT female [2 mount receipts]); thus, we do not report on mating behavior from the introduction.

#### Mating

24 h after the introduction, a clear, plastic barrier with 1 cm-diameter holes was placed into the cage to separate the male and female. Each side was given one plastic tube and half of the nest. The following day, the implanted animal was connected to a patch cable and given at least 5 min to habituate to fiber placement. The barrier was then removed, and behavior was recorded for 30 min. We never observed aggressive behavior from the male during the mating assay; thus, aggression is not reported in male mating results.

#### Partner preference test (PPT)

A PPT was conducted 6 h after pairing for females, or 3 days after mating for males. For females, animals were disconnected from the photometry system after the conclusion of the 30-min introduction, and the cage was assembled and placed in the housing room until the 6 h had elapsed. The PPT consists of a 3-chamber arena with an open top and 10 × 32in walls. Dividers extended 2 in into the arena on either side to separate the apparatus into 3 equal-sized chambers. The partner and an age-matched, opposite-sex stranger were tethered on either end of the arena, and the subject was given free access to all chambers for 3 h. To begin the assay, the implanted animal was connected to a patch cable, placed in the blocked-off center of the apparatus, and given at least 5 min to habituate to fiber tethering. The central barriers were then removed, and recordings lasted 3 h. The placement of the partner and stranger relative to the orientation of the room was varied randomly by vole. Only 1 animal (an *Oxtr*^*1−/−*^ male) displayed sexual behavior during the PPT (mounting with intromission with the stranger: 70 instances, 4.49% of total assay time); thus, we do not report on mating behavior from the PPT.

#### Separation and reunification

The implanted vole was connected to the patch cable and given at least 5 min to habituate to fiber placement. The partner vole was then removed from the home cage and placed in a separate, clean cage out of sight of the experimental vole. After one hour of separation, the partner was returned to the home cage, and behavior was recorded for 30 min. We never observed aggressive behavior from any animal during the reunion assay; thus, aggression is not reported in reunion results. Sexual behavior was rare during the reunion assay (2 out of 8 WT males, 0 out of 12 *Oxtr*^*1−/−*^ males, 2 out of 9 WT females, and 2 out of 11 *Oxtr*^*1−/−*^ females exhibited mounting without intromission, and 1 WT female exhibited mounting receipt with intromission); thus, mating is not reported in reunion results.

#### Stranger rejection

The implanted vole was connected to a patch cable and given at least 5 min to habituate to fiber placement. The partner vole was then removed from the home cage and placed in a separate, clean cage out of sight of the experimental vole. After one hour of separation, a novel, age-matched, opposite-sex vole was placed in the home cage, and behavior was recorded for 20 min. Sexual behavior was rare during the stranger rejection assay (2 out of 10 WT males, 0 out of 9 *Oxtr*^*1−/−*^ males, and 0 females exhibited mounting without intromission); thus, mating is not reported in stranger rejection results.

### Behavioral data analysis

Behavior scoring: Behavior was hand-scored frame by frame with the open-source software Boris (v. 7.11.1 - 2021-09-09)^89^. Behaviors were scored, and their criteria are detailed in Table 1. The TDT photometry software recorded time stamps of each acquired behavior video frame as well as time stamps of neural data, allowing for synchronization of the two data streams. Scoring was conducted by independent observers blind to genotype and partner location.

For each animal, we quantified the number, total duration as a percentage of the assay duration, and mean or median bout duration in seconds of each behavior and of social bouts. As described in Table 1, a social bout was defined as a sequence of behaviors initiated by non-AG sniffing, AG sniffing, or side-by-side contact, in which each behavior within the sequence was separated by less than 2 s of no interaction. For the PPT, chamber entries and exits were scored when the animal placed more than half of its body into an adjacent chamber. The start of an assay was defined as the moment when the stimulus animal was placed in the cage (having all 4 paws on the floor of the cage) or when the barriers dividing animals were fully removed from the cage (reaching the top edge of the cage or arena).

### Photometry Analysis

We illuminated each channel at sinusoidally varying intensity using different modulation frequencies to improve the signal-to-noise ratio with a lock-in amplifier system offline^17^. This system, coded in custom MATLAB software (version R2022b), involved performing a fast Fourier transform on the data from each channel to analyze the frequency domain between 100 and 500 Hz. The magnitudes (complex modulus) of the frequency-domain data streams were averaged across a band around the modulation frequency corresponding to each channel. We performed this calculation at each time point; this method identified deviations from the modulation frequency that reflect the biological signal. We then low-pass IIR filtered this transformed data at 100 Hz to eliminate fluctuations due to technical noise.

The fluorescence data from both streams were trimmed to eliminate technical noise at the beginning of the recording. To estimate fluctuations in the GCaMP channel that are also present in the control channel and therefore due to noise across the system, we estimated a robust linear regression fitting the data from our control channel (tdTomato) to the GCaMP channel and found the value at each time point. We then calculated the difference between the GCaMP data and the fitted control fluorescence at each time point and normalized this difference by the fitted control fluorescence to result in our final ΔF/F value at each time point.

We extracted a photometry ΔF/F trace surrounding each behavioral timestamp of interest. For social bouts or individual behavior timestamps, we extracted photometry data from −2 to 5 s relative to each timestamp. Timestamps of behaviors occurring during periods of experimenter intervention were excluded from analyses. For each ΔF/F trace, we Z-scored the trace to the mean and standard deviation of the ΔF/F values immediately prior to the timestamp (−2 to 0 s for social bouts). This method was chosen, in part, because we observed that NAc activity decreased below baseline during prolonged periods of quiet restfulness (Supplementary Fig. 12a–e). In addition, across all assays, we observed a notable increase in calcium signal that often preceded the introduction of the stimulus animal or removal of barriers, which may reflect a novelty, arousal, or fear response^90,91^ (Supplementary Fig. 12f–m). This signal decayed to pre-assay levels within roughly 100 s (Supplementary Fig. 12g). This prolonged increase in signal upon initiation of assays of social behavior was not observed when a vole was water restricted for 3 h and given access to a 10% sucrose solution (Supplementary Fig. 12n; see “Sucrose reward and social assay” methods below). For social touch behaviors, we excluded bouts in which social behavior (such as a strike or sniffing) occurred within the 2 s prior to the timestamp of interest. For strikes and chamber transitions, we excluded timestamps in which a strike or chamber transition occurred within the 2 s baseline period.

To quantify calcium activity traces, we calculated peak ΔF/F and area under the curve (AUC) of each trace from 0 to 2 s after each timestamp. AUC was calculated using Matlab’s “trapz” function. To confirm that the fluctuations and differences we observed in our ΔF/F traces were not due to motion artifacts, we used the same trace extraction and z-scoring method on the tdTomato control fluorescence. This demonstrated there was little effect of motion at the onset of behaviors such as sniffs or even behaviors that entail a great deal of movement, such as attacks (Supplementary Fig. 12o–r).

### Sucrose reward and social assay

To determine the calcium activity response of the NAc to reward, we expressed GCaMP6m and tdTomato in the medial NAc core of a sexually naive, group-housed male vole and implanted an optic fiber as described above. The vole was deprived of water for 3 h prior to testing and given 5 min to acclimate to fiber placement in a clean cage. We then presented a water spout offering access to sucrose water (10% by weight) for ten trials. The vole was given 15 s of access to the spout upon initiation of licking during each trial, and the inter-trial interval was 60 s. Five minutes after the sucrose assay, a sexually naive, group-housed female vole was introduced to the cage, and the animals were allowed to interact for 5 min. Licking and social behaviors were hand-scored frame by frame in Boris.

### Perfusions, histology, and verification of fiber placement

Animals were deeply anesthetized with ketamine/xylazine and transcardially perfused with 1X phosphate-buffered saline (PBS) followed by cold 4% paraformaldehyde (PFA) in PBS. Brains were extracted, post-fixed in PFA overnight at 4 °C, and incubated in 30% sucrose solution at 4 °C until sucrose diffused completely through the brain. Brains were then embedded in Tissue-Tek Optimal Cutting Temperature Compound (Sakura Finetek USA, Torrance, CA), frozen solid at −80 °C, cryosectioned at 50 uM coronally, and treated with 300 nM DAPI solution (Life Technologies, Carlsbad, CA) in PBS for 10 min to visualize nuclei. Sections were mounted on glass slides and coverslipped with Aqua-Mount Mounting Medium (Thermo Scientific).

Brain sections were imaged with a 4X objective on a Nikon Eclipse 90i motorized upright epifluorescent microscope and digital camera (Nikon, Minato City, Tokyo, Japan) as well as a 10X objective on a Zeiss LSM 700 confocal microscope with Zen 2010 software (Zeiss Microscopy). Fiber tip locations were assessed by comparing the anatomical location of the fiber tract to the Paxinos and Franklin mouse brain atlas (4th edition)^92^. Animals were excluded from behavioral and photometry analyses if the fiber tip was outside of the nucleus accumbens or outside the range of 1.10–1.78 mm relative to Bregma (of the mouse atlas).

### Statistics

Details of all statistical tests used and their results are reported in Supplementary File 1. Sample sizes were determined by our previous work investigating pair-bonding behaviors in *Oxtr*^*1−/−*^ prairie voles^11^ and increased by ~20% to account for animals lost to surgery failure or photometry exclusion. Alpha was set to 0.05 for all comparisons. Trends were considered when p < 0.07. All statistical tests were two-sided. Recording experiments were not independently replicated.

#### Behavior data

For behavior data, statistics and plotting were performed in Prism (version 9.4 for MacOS). In each assay, the metric used to determine outlier status was total social interaction. Animals that were 3 scaled median absolute deviations (MAD) away from the median were deemed outliers and were removed before proceeding. Details on outliers are provided in Supplementary File 1. We assessed normality of residuals by the D’Agostino-Pearson omnibus (K2) test. Homogeneity of variance was evaluated via the F test. For continuous and normally distributed measures, we conducted a Student’s t-test, ANOVA, two-way ANOVA, or two-way repeated measures ANOVA with Sidak-corrected *post hoc* comparisons. A Welch’s corrected t-test was used when distributions failed an F-test for equality of variances. For distributions that were not normally distributed, we first applied a log transform and retested for normality. Parametric tests were then run on the normalized data. For distributions that could not be log transformed, we used the non-parametric Mann–Whitney test for comparisons between 2 groups or a two-way ANOVA on the ranks of the data for more than 2 groups. We plotted the data in raw form when feasible. For count data, we used a generalized linear model with a generalized Poisson distribution and a Sidak correction for multiple comparisons to compare between groups. Transformations conducted on each data set prior to statistical testing can be found in Supplementary File 1. For proportion data, we used a binomial test to compare genotypes or groups. To characterize the relationship between behavior at 2 time points (e.g., the introduction and the PPT), we used Pearson correlation or linear regression. To compare behavior across male preference categories (i.e., wild-type, partner preferring *Oxtr*^*1−/−*^, and stranger-preferring *Oxtr*^*1−/−*^), we used a permutation test on the F statistic. For the permutation test, group labels were randomly shuffled, and the statistic was calculated. This was repeated 10,000 times to construct a null distribution, and the *p*-value was calculated as the percentage of the null distribution that was equal to or more extreme than the observed statistic. *Post hoc* pairwise comparisons were conducted with additional permutation tests with a Sidak correction for multiple comparisons.

For the PPT, preference indices were calculated for the total percent of PPT time spent in either social interaction (“Social preference index”) or in side-by-side contact (“Side-by-side preference index”). Preference indices were calculated by taking the percent time spent with the partner, subtracting the time spent with the stranger, and normalizing by total time spent with either partner or stranger. Preference index values were compared across genotypes by performing a permutation test, as described above, on the Earth Mover’s Distance between the two distributions.

#### Markov chains

We performed discrete-time Markov chain analyses on behavioral sequences from the introduction assay. We included only “no interaction,” “non-AG sniff,” “AG sniff,” and “side-by-side contact” due to the fact that all other behavioral events were rare (transition probabilities less than 0.005). Markov chain transition probability matrices were calculated for each animal in Matlab, and probabilities for each transition were averaged across sex and genotype. For each transition, we compared transition probabilities across genotypes or groups by conducting permutation tests, as described above, on the Student’s t statistic or F statistic and applying a Sidak correction for multiple comparisons.

#### Adjusted boxplots

When plotting neural data surrounding behavioral events with duration (*e.g*., AG sniff events), we included boxplots of those initiating behavior durations. Because duration distributions were heavily right-skewed, we used an adjusted boxplot for skewed distributions^93^. Briefly, the medcouple (MC, a measure of skewness) is calculated for each distribution of duration values. The MC is then incorporated in the determination of the whisker boundaries, such that when MC > = 0, whisker boundaries are defined as [*Q*_1_ − 1.5e^−4 MC^ IQR; *Q*_3_ + 1.5e^3,MC^ IQR] and when MC < 0, boundaries are [*Q*_1_ − 1.5e^−3 MC^ IQR; *Q*_3_ + 1.5e^4,MC^ IQR], where *Q*_1_ is the first quartile, *Q*_3_ is the third quartile, and IQR is the interquartile range (*Q*_3_ − *Q*_1_). Outlier values are marked with a plus symbol. The adjusted boxplot was implemented with an adapted version of the function “adjusted_boxplot” written by Brian C. Coe (2024, MATLAB Central File Exchange, https://www.mathworks.com/matlabcentral/fileexchange/72110-adjusted_boxplot).

#### Probability PETHs

To examine the probability of the occurrence of different behaviors surrounding timestamps of interest, we constructed peri-event time histograms (PETHs) of probability (e.g., Supplementary Fig. 4g). For each timestamp and each time point along the PETH, a given behavior was marked as occurring (1) or not occurring (0). We then averaged by group across each time point.

#### Photometry data

All calculations and statistics for photometry data were performed in MATLAB. The majority of peak ΔF/F and AUC distributions were non-normally distributed as determined by the Jarque-Bera test; thus, for all comparisons, we pooled data from all groups and applied a Box-Cox transformation prior to testing. We then removed outlier traces. The criterion for removal was when both peak and AUC values for a given trace were 3 scaled MAD away from the median of the pooled data from all comparison groups. We tested for equality of variance across groups via Levene’s test. We tested for main effects and interactions between independent variables (*e.g*., genotype, stimulus animal, etc.) via a linear mixed effects (LME) model in which vole ID was included as a random effect to account for clustering within animals. The resulting model was run through an ANOVA, and we report the F-statistics and *p*-values. Multiple comparisons were conducted by pairwise LME models with a Sidak correction applied to the resulting *p*-values. In addition, we compared z-scored ΔF/F values at each time point along the PETH. At each point, we Box-Cox transformed the pooled z-scored ΔF/F values. We then conducted an LME with vole ID included as a random effect, as described above. This was repeated for every time point and every pairwise comparison of, for example, genotype by stimulus animal. We then applied a Benjamini-Yekutieli correction on the resulting *p*-values for *all* pairwise comparisons between groups.

To obtain mean neural responses by individual animal, we calculated the mean of the Box-Cox transformed values of the neural data. We used the transformed values to relate neural data to behavior or other neural metrics by individual animal via Pearson correlation or linear regression.

To test whether NAc calcium activity changed as an animal engaged in prolonged rest (Supplementary Fig. 12a–e), we calculated median raw ΔF/F and AUC for each trace from −30 s to 0 s (pre) and 0 s to 30 s (post). We Box-Cox transformed all values and conducted an LME with time (pre versus post) as the independent variable and both vole ID and trace number as random effects. We next calculated the change in ΔF/F (post – pre) for each trace and averaged by individual animal. We then used a two-tailed, one-sample t-test to determine whether the change in ΔF/F was significantly different from 0 ΔF/F. To test how activity changed at the start of an assay, we constructed PETHs for every animal and every assay from −10 s to 180 s surrounding assay start (Supplementary Fig. 12f, g). We calculated the mean raw ΔF/F value from −10 s to 0 s to be 0.0237. At each time point along the PETH, we Box-Cox transformed all values, including the constant of 0.0237 and conducted an LME with vole ID as a random effect to compare whether ΔF/F was significantly different from the transformed constant. We applied a Benjamini-Yekutieli correction to the resulting set of *p* values.

#### Survival regressions

Survival analyses were conducted in R. We modeled the relationship between NAc calcium activity during a social bout and the latency to engage in a subsequent social bout. Specifically, we used a mixed effects Cox regression to predict latency as a function of sex, genotype, and z-scored peak or AUC of calcium activity values (Box-Cox transformed, excluding outlier values as described above), with vole identity included as a random effect. When a significant sex by genotype by neural data interaction was found, we conducted a stratified analysis in which latency was modeled as a function of genotype, neural data, and vole identity for each sex separately. When a significant genotype by neural data interaction was found, we conducted an additional stratified analysis in which latency was modeled as a function of neural data and vole identity for each genotype separately. Resulting *p* values from *post hoc* analyses were Sidak corrected.

#### Plotting

Bar plots show mean ± s.e.m. with individual animals overlaid. Neural data is plotted as mean ± s.e.m. Swarm plots of behavior data show individual animals with a median overlaid. Swarm plots of neural data show individual PETH values (peak or AUC) with median overlaid. Dotted lines on plots of linear regressions show the 95% confidence interval.

### Reporting summary

Further information on research design is available in the Nature Portfolio Reporting Summary linked to this article.

## Supplementary information

Supplementary Information

Peer Review file

Description of Additional Supplementary Files

Supplementary Data 1

Reporting Summary

## Source data

Source Data 1

Source Data 2

## Data Availability

All behavior data associated with this study are included as a supplemental file “Long_NatComms_BehaviorData.xlsx”. Neural data, videos, and associated metadata are publicly available on the DANDI Archive (RRID:SCR_017571) at the following URL: https://dandiarchive.org/dandiset/001700. Source data are provided with this paper.

## Code availability

Custom Matlab code is available on the Manoli Lab GitHub at https://github.com/manolilab/Long_et_al_NatComms2026.
