# Supplementary material for: Loss of oxytocin receptor function disrupts neural signatures of pair bonding and fidelity in the nucleus accumbens
Source: Nat Commun. 2026 Sep 12;17:10755. doi: 10.1038/s41467-026-76876-8 (PMC13661945; doi:10.1038/s41467-026-76876-8)
Supplement: Supplementary file 1 [file 41467_2026_76876_MOESM1_ESM.pdf]

Loss of oxytocin receptor function disrupts neural signatures of pair bonding and fidelity in the nucleus accumbens

Supplementary Information

Kimberly L. P. Long[1,2,3,4], Nerissa E. G. Hoglen[1,2,3,4,5], Alex J. Keip[1,2,3,4,5], Robert M. Klinkel[1,2,3,4], DéJenaé L. See[1,2,3,4], Joseph Maa[6], Jenna C. Wong[6], Michael Sherman[1,2,3,4], and Devanand S. Manoli[1,2,3,4]*

[1]Department of Psychiatry and Behavioral Sciences, University of California, San Francisco; San Francisco, CA 95158, USA.

[2]Center for Integrative Neuroscience, University of California, San Francisco; San Francisco, CA 95158, USA.

[3]Weill Institute for Neurosciences, University of California, San Francisco; San Francisco, CA 95158, USA.

[4]Kavli Institute for Fundamental Neuroscience, University of California, San Francisco; San Francisco, CA 95158, USA.

[5]Neurosciences Graduate Program, University of California, San Francisco; San Francisco, CA 95158, USA.

[6]Department of Molecular and Cell Biology, University of California, Berkeley; Berkeley CA 94720, USA.

***To whom correspondence should be addressed:** devanand.manoli@ucsf.edu

Extended Data Figures

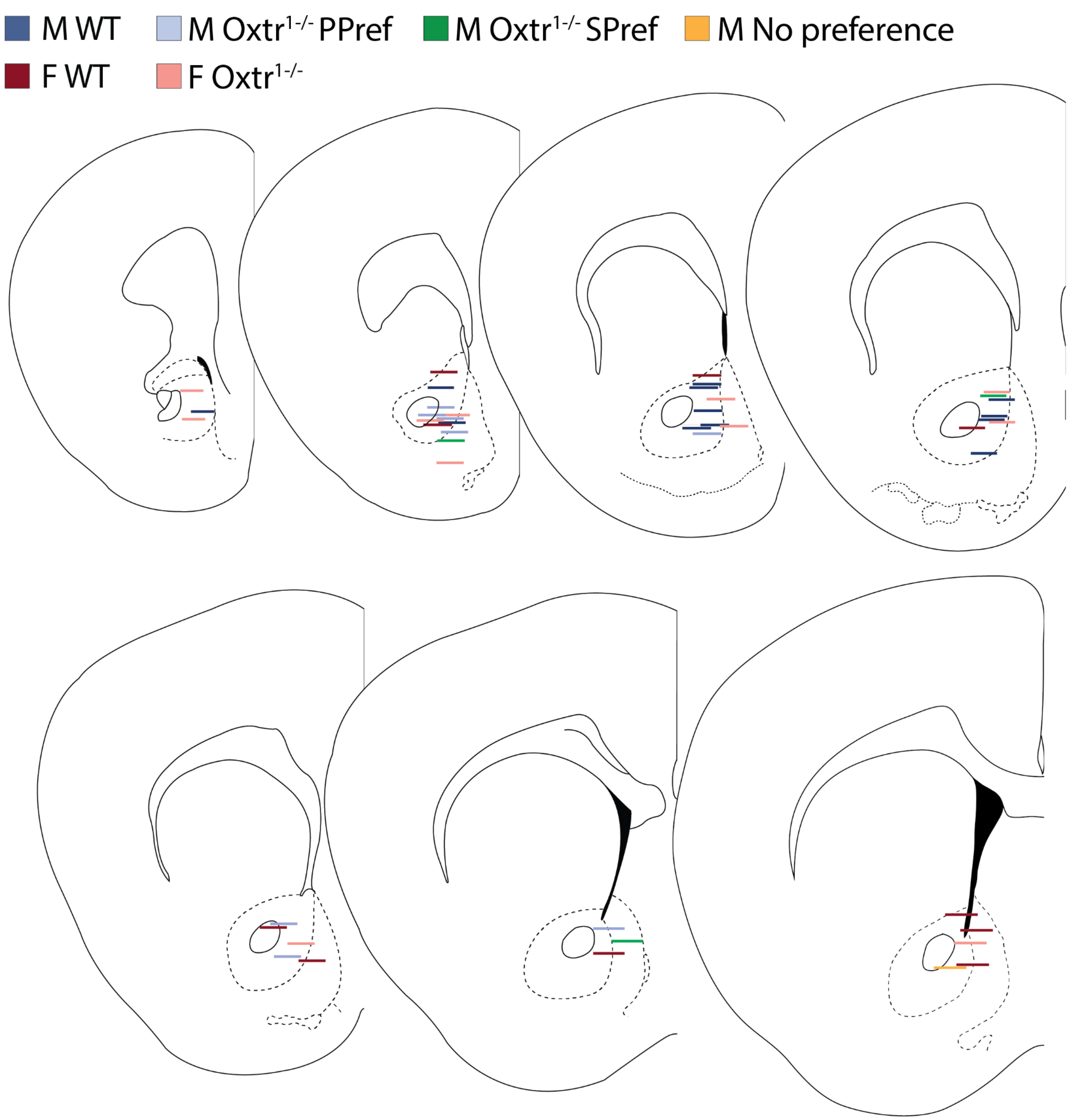

Supplementary Figure 1: Fiber locations in the prairie vole nucleus accumbens. M, male; F, female; PPref, partner-preferring; SPref, stranger-preferring.

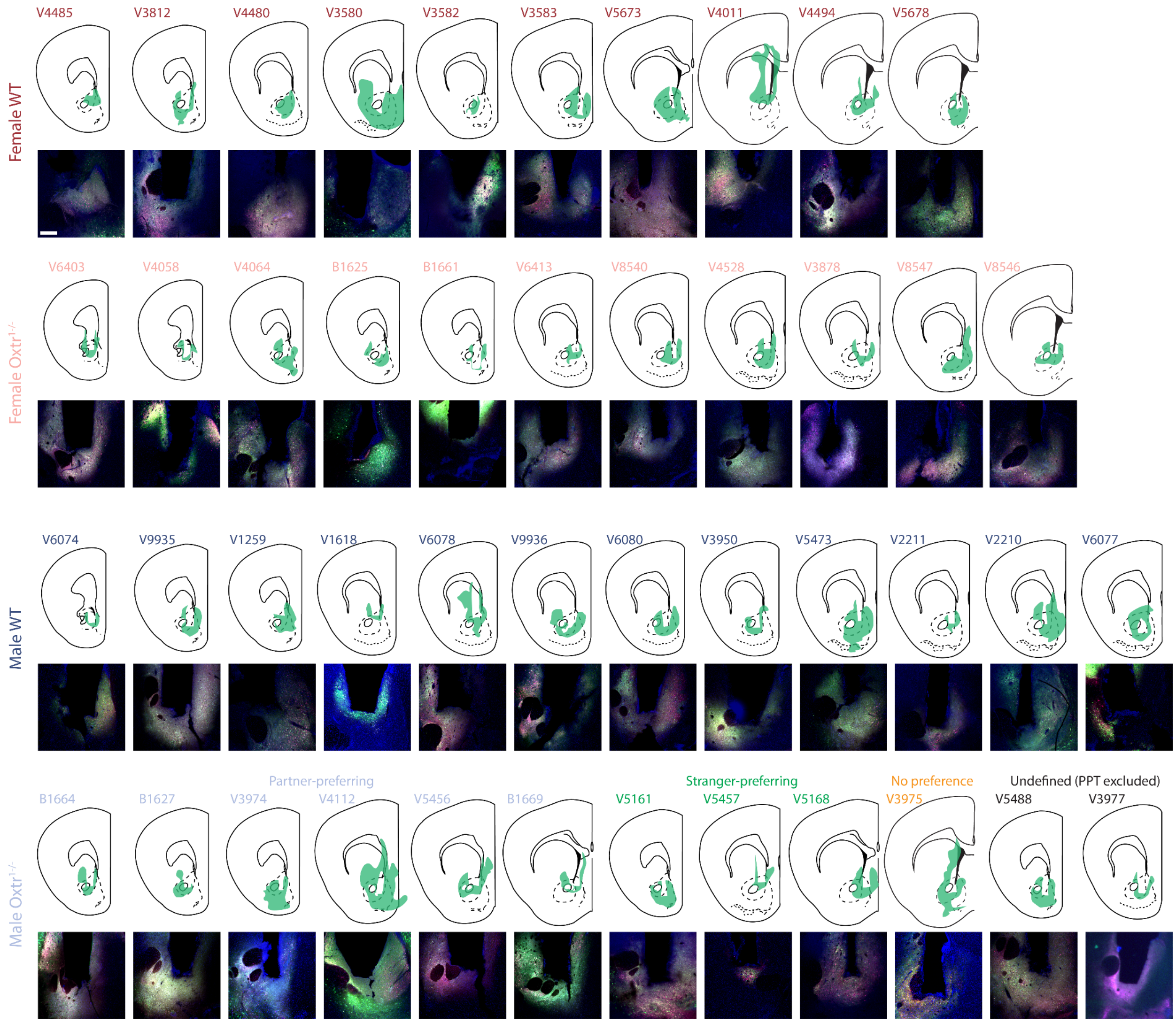

Supplementary Figure 2: Fiber locations and viral expression per animal. Templates were adapted from the Paxinos and Franklin mouse atlas. Green outlines indicate the extent of hSyn-GCaMP6m expression. Below each template is an image of the fiber tip (blue - DAPI; green - GCaMP6m; magenta - tdTomato). Scale bar on top left image: 250 µm.

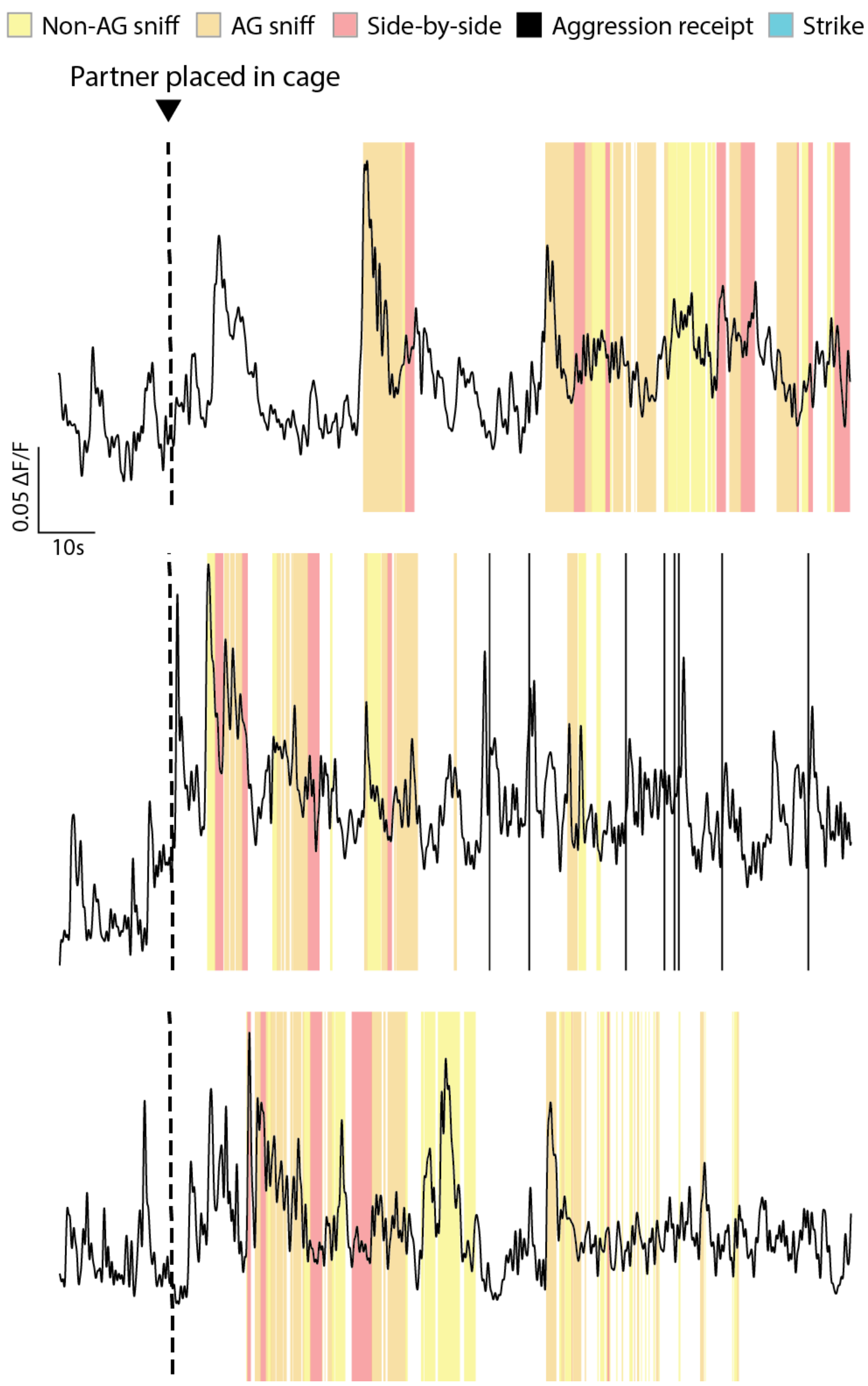

Supplementary Figure 3: Additional examples of raw ΔF/F traces. Additional examples of ΔF/F GCaMP6m activity at the onset of the introduction.

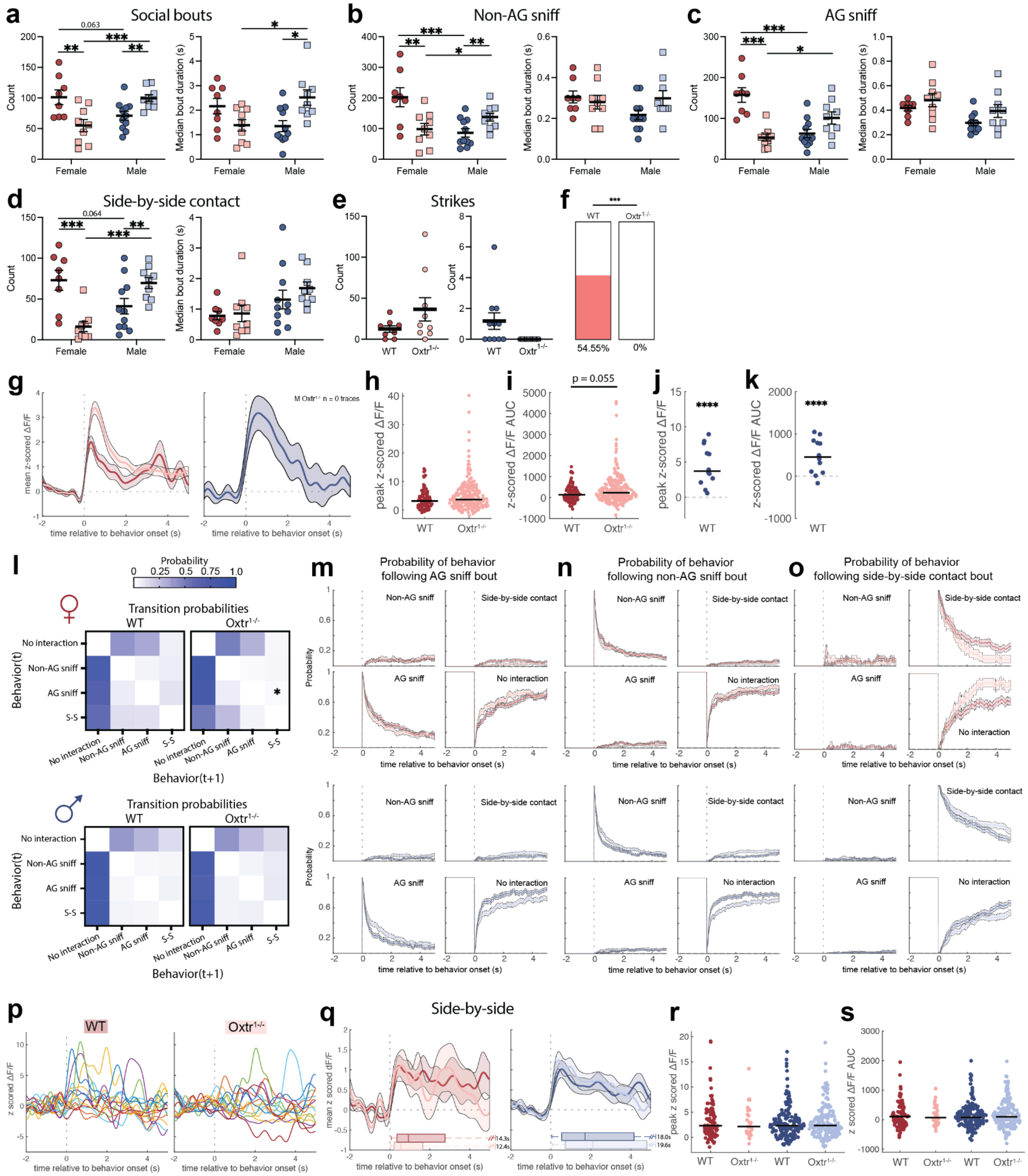

Supplementary Figure 4: Additional data from introductions. a, Number (left) and median duration (right) of social bouts (for all plots, female WT n=8 voles, female Oxtr$^{1-/-}$ n=9 voles, male WT n=11, male Oxtr$^{1-/-}$ n=9). **b**, Number and median duration of all non-anogenital (non-AG) sniffs. **c**, Number and median duration of all anogenital (AG) sniffs. **d**, Number and median duration of

all side-by-side contacts. **e**, Number of strikes directed at the novel partner. Females and males are plotted separately due to the difference in scale. **f**, Proportion of males displaying any offensive strikes during the introduction. **g**, Mean z-scored ΔF/F traces (+/- s.e.m.) at the onset of strikes (female WT n=93 traces/7 voles, $Oxtr^{1-/-}$ n=281 traces/8 voles). The sexes were compared separately due to the absence of strikes exhibited by $Oxtr^{1-/-}$ males. **h**, Peak z-scored ΔF/F values of female strikes. **i**, AUC values of z-scored ΔF/F traces of female strikes. **j**, Peak z-scored ΔF/F values of WT male strikes. **k**, AUC values of z-scored ΔF/F traces of WT male strikes. **l**, Heat maps of transition probabilities from Markov modeling of behavior from females (top) and males (bottom). **m**, Probability of social touch behaviors following the onset of an AG sniff bout. Data are related to that shown in Fig. 1n. **n**, Probability of social touch behaviors following the onset of a non-AG sniff bout. Data are related to that shown in Fig. 1q. **o**, Probability of social touch behaviors following the onset of a side-by-side contact bout. **p**, Fifteen randomly sampled examples of z scored ΔF/F at the onset of anogenital sniffing of the partner in wild-type (WT, left) and $Oxtr^{1-/-}$ (right) female prairie voles. **q**, Mean z-scored ΔF/F traces at the onset of social bouts initiated by side-by-side contact. At the base of the plot is an adjusted boxplot of the durations of the initiating behavior (female WT n=94 traces, female $Oxtr^{1-/-}$ n=33 traces, male WT n=172 traces, male $Oxtr^{1-/-}$ n=172 traces). **r**, Peak z-scored ΔF/F values related to side-by-side contact. **s**, AUC values of z-scored ΔF/F traces related to side-by-side contact. Detailed statistics are presented in Extended Data File 1. *p<0.05, **p<0.01, ***p<0.001, ****p<0.0001. AG, anogenital; WT, wild-type; S-S, side-by-side contact; AUC, area under the curve. Source data are provided as a Source Data file.

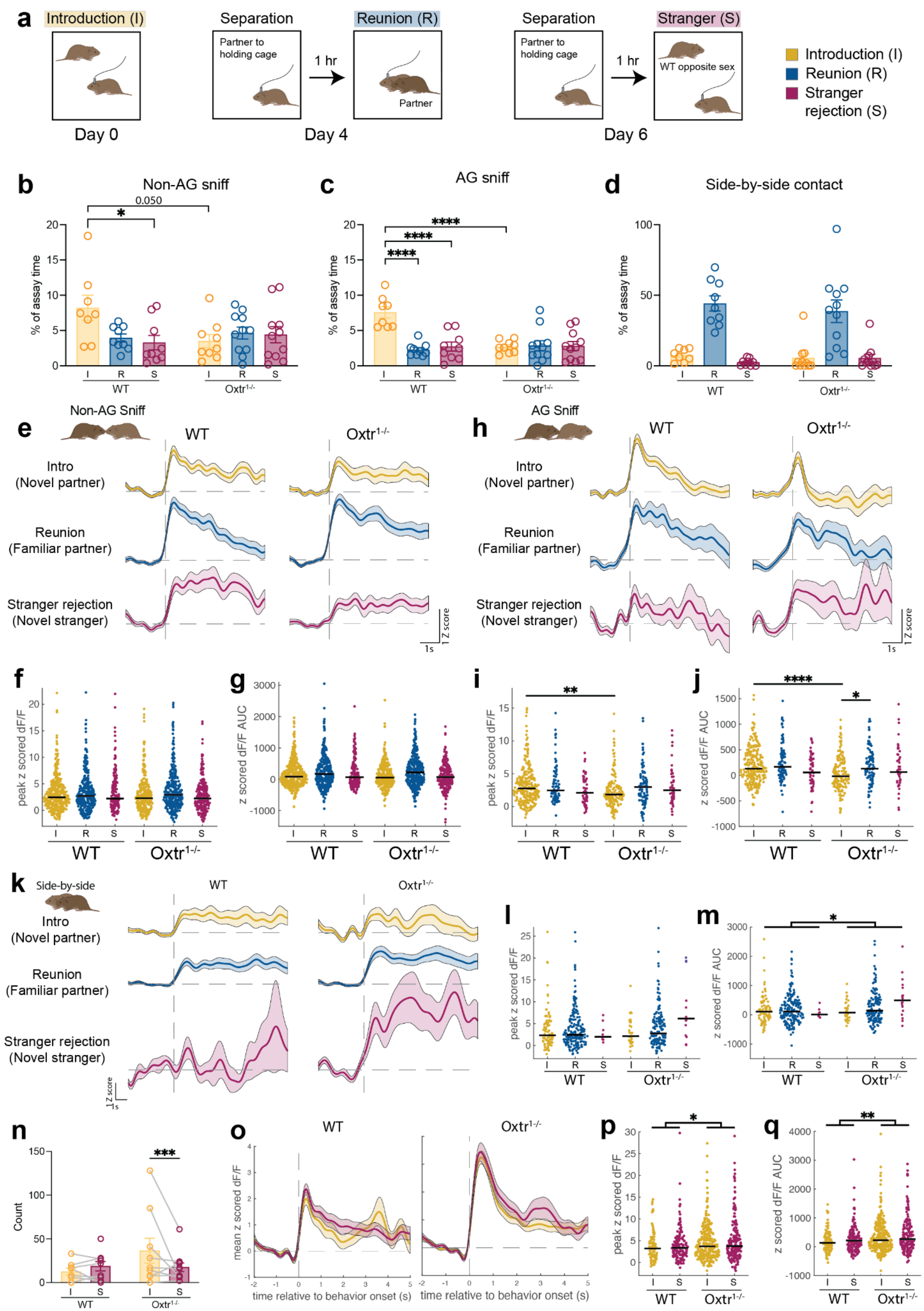
a
Introduction (I)
Separation
Reunion (R)
Separation
Stranger (S)
Partner to holding cage
1 hr
Partner
Partner to holding cage
1 hr
WT opposite sex
Day 0
Day 4
Day 6
Introduction (I)
Reunion (R)
Stranger rejection (S)
b
Non-AG sniff
0.050
% of assay time
WT
Oxtr1-/-
c
AG sniff
% of assay time
WT
Oxtr1-/-
d
Side-by-side contact
% of assay time
WT
Oxtr1-/-
e
Non-AG Sniff
WT
Oxtr1-/-
Intro (Novel partner)
Reunion (Familiar partner)
Stranger rejection (Novel stranger)
1 Z score
1s
h
AG Sniff
WT
Oxtr1-/-
Intro (Novel partner)
Reunion (Familiar partner)
Stranger rejection (Novel stranger)
1 Z score
1s
f
peak z scored dF/F
WT
Oxtr1-/-
g
z scored dF/F AUC
WT
Oxtr1-/-
i
peak z scored dF/F
WT
Oxtr1-/-
j
z scored dF/F AUC
WT
Oxtr1-/-
k
Side-by-side
WT
Oxtr1-/-
Intro (Novel partner)
Reunion (Familiar partner)
Stranger rejection (Novel stranger)
1 Z score
1s
l
peak z scored dF/F
WT
Oxtr1-/-
m
z scored dF/F AUC
WT
Oxtr1-/-
n
Count
WT
Oxtr1-/-
o
WT
Oxtr1-/-
mean z scored dF/F
time relative to behavior onset (s)
time relative to behavior onset (s)
p
peak z scored dF/F
WT
Oxtr1-/-
q
z scored dF/F AUC
WT
Oxtr1-/-

Supplementary Figure 5: Cross-assay analyses in females. a, Schematic of introduction (I), reunion (R), and stranger rejection (S) paradigms. **b**, Percent of assay time spent non-AG sniffing the stimulus animal across assays. **c**, Percent of time spent AG sniffing the stimulus animal across assays. **d**, Percent of time spent in side-by-side contact with the stimulus animal across assays (main effect of assay). **e**, Mean ΔF/F PETH aligned to non-AG sniffs during the introduction, reunion, and stranger rejection assays. Yellow, intro (WT n=394 traces from 8 animals; $Oxtr^{1-/-}$ n=273 traces from 9 animals); blue, reunion (WT n=326 traces from 9 animals; $Oxtr^{1-/-}$ n=354 traces from 10 animals); purple, stranger rejection (WT n=164 traces from 8 animals; $Oxtr^{1-/-}$ n=237 traces from 10 animals). **f**, Peak z-scored ΔF/F values (main effect of assay). **g**, AUC values from z-scored ΔF/F traces (main effect of assay). **h**, Mean ΔF/F PETH aligned to AG sniffs during the intro, reunion, and stranger rejection assays. (Intro: WT n=268 traces from 8 animals, $Oxtr^{1-/-}$ n=161 traces from 9 animals; Reunion: WT n=86 traces from 9 animals, $Oxtr^{1-/-}$ n=101 traces from 10 animals; Stranger rejection: WT n=54 traces from 8 animals, $Oxtr^{1-/-}$ n=47 traces from 10 animals). **i**, Peak ΔF/F values. **j**, AUC values from z-scored ΔF/F traces. **k**, Mean ΔF/F traces surrounding social bouts initiated with side-by-side contact during the introduction, reunion, and stranger rejection assays (Intro: WT n=95 traces from 8 animals; $Oxtr^{1-/-}$ n=33 traces from 6 animals; Reunion: WT n=190 traces from 9 animals; $Oxtr^{1-/-}$ n=148 traces from 11 animals; Stranger rejection: WT n=8 traces from 3 animals; $Oxtr^{1-/-}$ n=18 traces from 6 animals). **l**, Peak ΔF/F values. **m**, AUC values from z-scored ΔF/F traces (significant main effect of genotype). **n**, Number of strikes in the introduction and stranger rejection assays. Note that reunion is not included because no strikes were observed by any animal during this assay. **o**, Mean ΔF/F traces surrounding strikes. (Intro: WT n=93 traces from 6 animals; $Oxtr^{1-/-}$ n=278 traces from 8 animals; Stranger rejection: WT n=152 traces from 8 animals; $Oxtr^{1-/-}$ n=182 traces from 11 animals). **p**, Peak ΔF/F values (significant main effect of genotype). **q**, AUC values from z-scored ΔF/F traces

(significant main effect of genotype). Detailed statistics are presented in Extended Data File 1. *p<0.05, **p<0.01, ***p<0.001, ****p<0.0001. WT, wild-type; AUC, area under the curve; AG, anogenital; PETH, peri-event time histogram. Vole images were created in BioRender. Manoli, D. (2026) https://BioRender.com/00l1i4c. Source data are provided as a Source Data file.

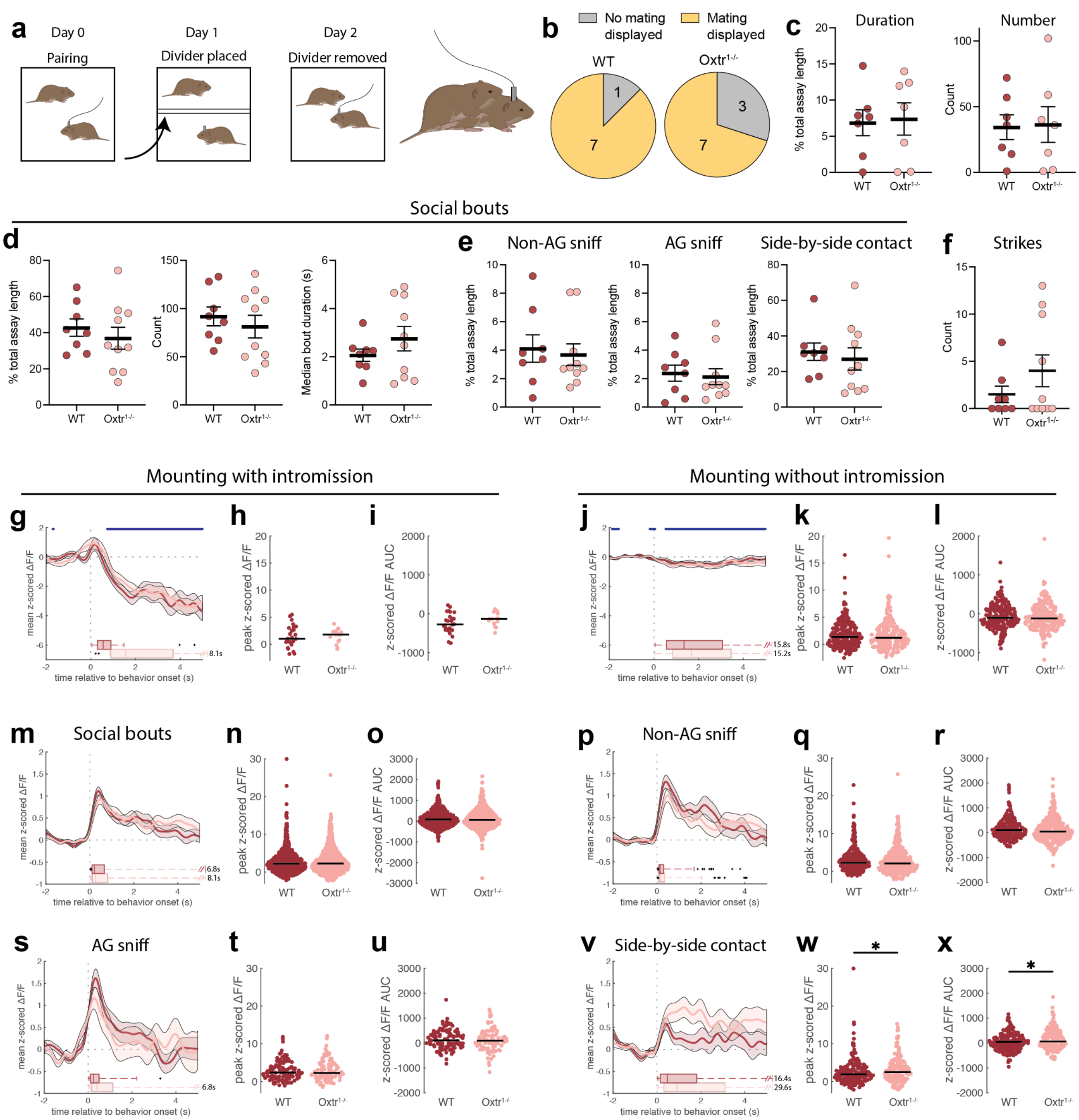

Supplementary Figure 6: Additional data from female timed mating assays. a, Timed mating procedure. Roughly 24 hours after introductions, we placed a clear plastic barrier with small holes in the cage to divide the animals into two compartments and to allow limited social contact. The following day, the focal animal was habituated to the patch cable, the barrier was removed, and behavior was recorded for 30 minutes. **b**, Mounting and intromission were scored when a female received mounting or intromission. Pie charts show the proportion of females that received mating behavior. **c**, Left, percent of assay time spent engaged in mating behavior, plotting only the females that received any mating attempts. Right, number of mating bouts, plotting only females that received any mating attempts (n=7 per group). **d**, Quantification of social bouts. Left, total amount of assay time spent engaged in social bouts. Middle, number of social bouts. Right, median duration of social bouts (for subsequent plots, WT n=8 voles, $Oxtr^{1-/-}$ n=10 voles). **e**, Percent of timed mating assay time spent engaged in non-anogenital (AG) sniffing, AG sniffing, or side-by-side contact. **f**, Number of strikes by females directed at the partner. **g**, Mean z-scored ΔF/F traces at the onset of mounts that transition to intromission. Blue bars at the top of the plot indicate time points at which the combined WT and $Oxtr^{1-/-}$ mean z-scored ΔF/F is significantly less than 0. At the base of the plot is an adjusted boxplot of the durations of the initiating behavior (WT n=26 traces/3 voles, $Oxtr^{1-/-}$ n=16 traces/3 voles). **h**, Peak z-scored ΔF/F values. **i**, AUC values of z-scored ΔF/F traces. **j**, Mean z-scored ΔF/F traces at the onset of mounts that do not transition to intromission (WT n=233 traces/7 voles, $Oxtr^{1-/-}$ n=244 traces/7 voles). **k**, Peak z-scored ΔF/F values. **l**, AUC values of z-scored ΔF/F traces. **m**, Mean z-scored ΔF/F traces at the onset of social bouts (WT n=621 traces, $Oxtr^{1-/-}$ n=663 traces). **n**, Peak z-scored ΔF/F values. **o**, AUC values of z-scored ΔF/F traces. **p**, Mean z-scored ΔF/F traces at the onset of social bouts initiated by non-AG sniffing (WT n=278 traces, $Oxtr^{1-/-}$ n=378 traces). **q**, Peak z-scored ΔF/F values. **r**, AUC values of z-scored ΔF/F traces. **s**, Mean z-scored ΔF/F traces at the onset of social

bouts initiated by AG sniffing (WT n=118 traces, Oxtr$^{1-/-}$ n=103 traces). **t**, Peak z-scored ΔF/F values. **u**, AUC values of z-scored ΔF/F traces. **v**, Mean z-scored ΔF/F traces at the onset of social bouts initiated by side-by-side contact (WT n=224 traces, Oxtr$^{1-/-}$ n=180 traces). **w**, Peak z-scored ΔF/F values. **x**, AUC values of z-scored ΔF/F traces. Detailed statistics are presented in Extended Data File 1. *p<0.05. WT, wild-type; AUC, area under the curve; AG, anogenital. Vole images were created in BioRender. Manoli, D. (2026) https://BioRender.com/00l1i4c. Source data are provided as a Source Data file.

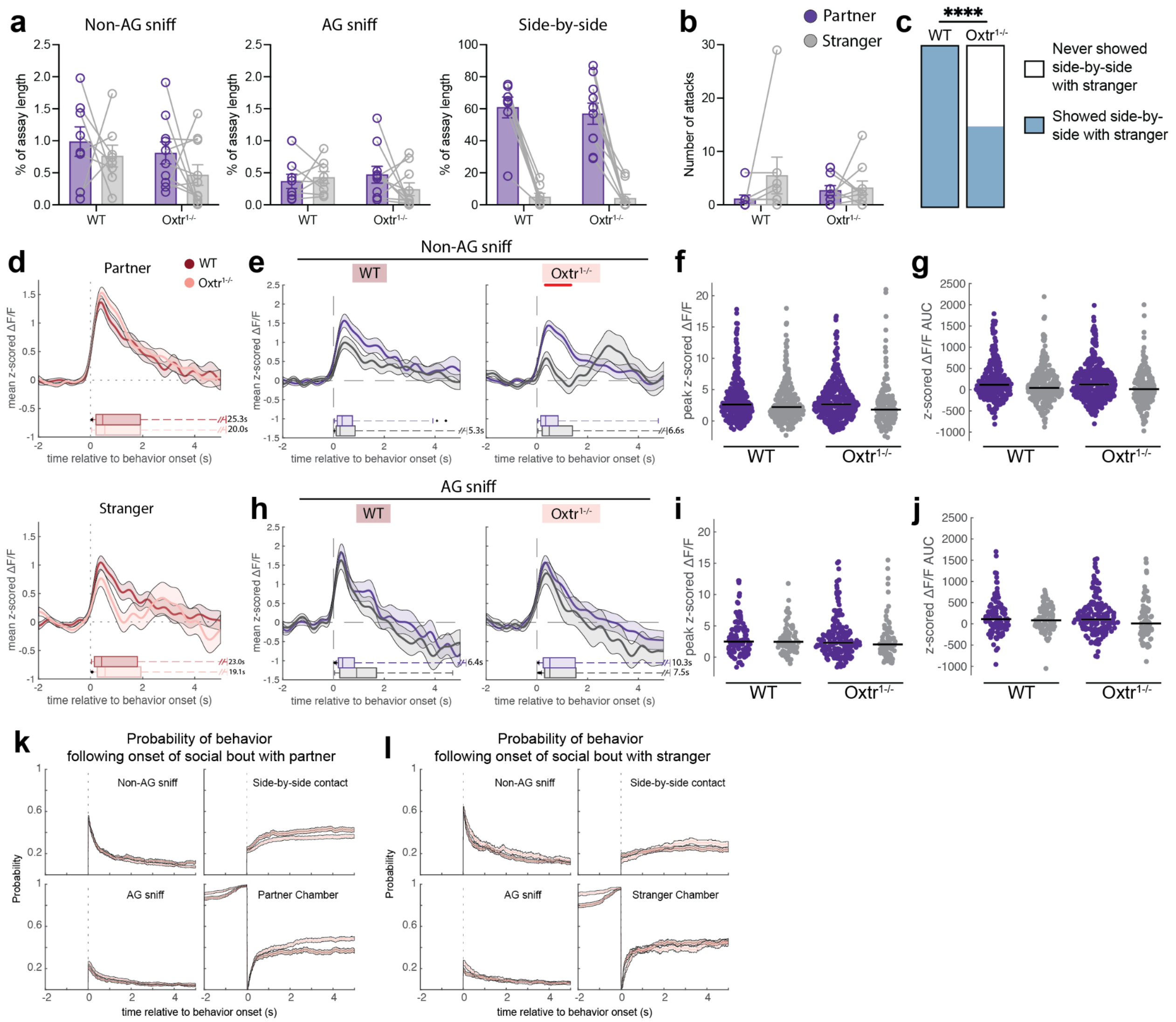

Supplementary Figure 7: Additional data from female partner preference tests. a, Percent of PPT assay time spent engaged in non-anogenital (AG) sniffing, AG sniffing, or side-by-side contact. Note the difference in the y-axis for side-by-side contact (for all plots, WT n=8 voles, Oxtr$^{1-/-}$ n=10 voles). **b**, Number of attacks against the partner or stranger male. **c**, Proportion of females that displayed at least one bout of side-by-side contact with the stranger male. **d**, Mean z-scored ΔF/F (+/- s.e.m.) traces at the onset of social bouts with either the partner (top) or stranger (bottom). Data are the same as those plotted Fig. 2f. At the base of the plot is an adjusted boxplot of the durations of the initiating behavior. **e**, Mean z-scored ΔF/F traces at the onset of

social bouts initiated by non-AG sniffing. The red line indicates time points at which mean z-scored ΔF/F differs between partner and and stranger ($WT_{Partner}$ n=291 traces, $WT_{Stranger}$ n=276 traces, $Oxtr^{1-/-}_{Partner}$ n=383 traces, $Oxtr^{1-/-}_{Stranger}$ n=188 traces). **f**, Peak z-scored ΔF/F values (significant main effects of stimulus animal and genotype). **g**, AUC values of z-scored ΔF/F traces (significant main effect of stimulus animal). **h**, Mean z-scored ΔF/F (+/- s.e.m.) traces at the onset of social bouts initiated by AG sniffing ($WT_{Partner}$ n=113 traces, $WT_{Stranger}$ n=83 traces, $Oxtr^{1-/-}_{Partner}$ n=185 traces, $Oxtr^{1-/-}_{Stranger}$ n=77 traces). **i**, Peak z-scored ΔF/F values. **j**, AUC values of z-scored ΔF/F traces. **k**, Probability of social touch behaviors following the onset of a social bout with a partner. Data are related to that shown in d (Partner). **l**, Probability of social touch behaviors following the onset of a social bout with a stranger. Data are related to that shown in d (Stranger). Detailed statistics are presented in Extended Data File 1. *p<0.05, **p<0.01, ***p<0.001, ****p<0.0001. AG, anogenital; WT, wild-type; AUC, area under the curve. Source data are provided as a Source Data file.

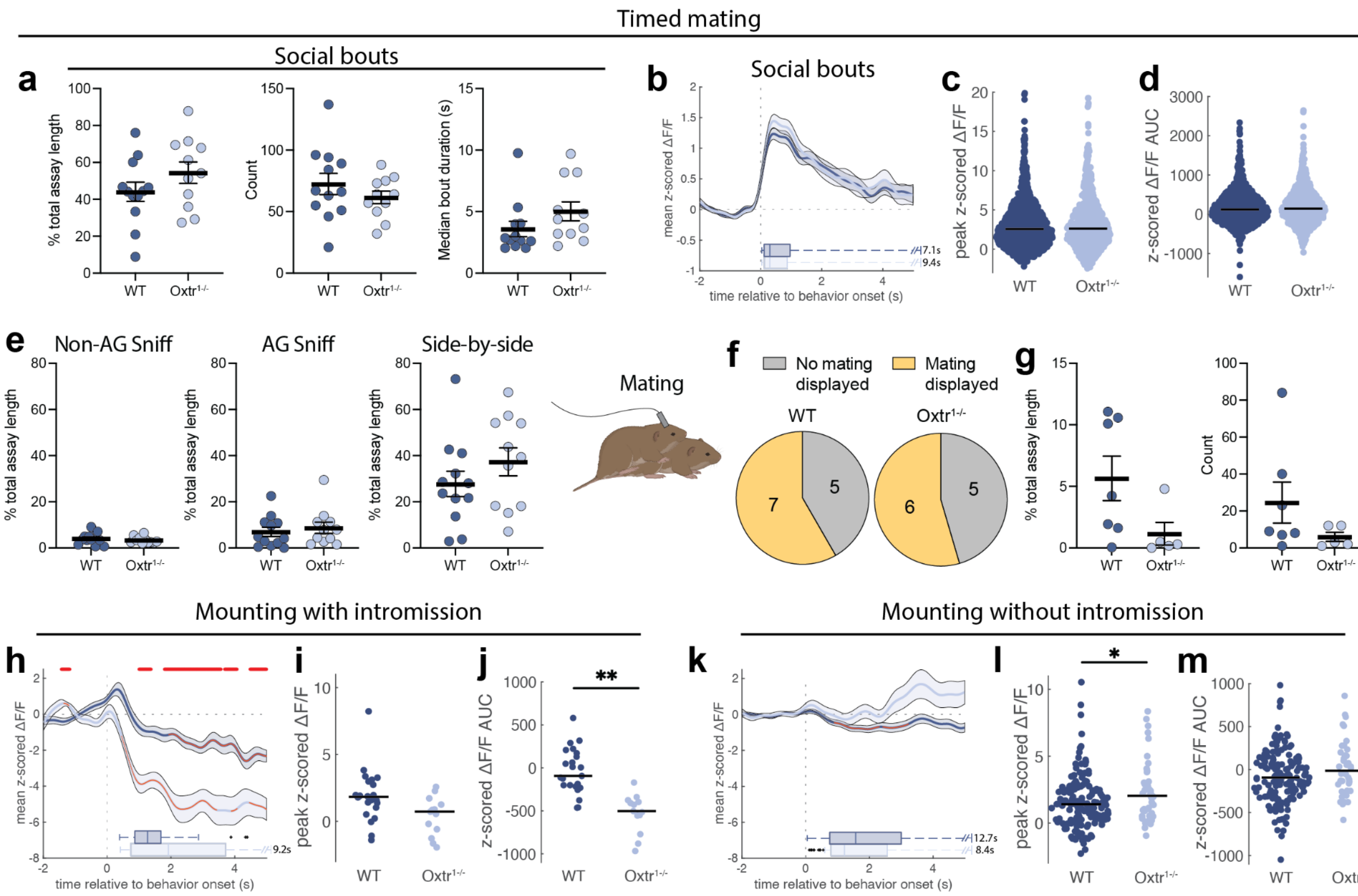

Supplementary Figure 8: Additional data from male timed mating assays. a, Quantification of social bouts during the timed mating assay. Left, total amount of assay time spent engaged in social bouts. Middle, number of social bouts. Right, median duration of social bouts (WT n=12, Oxtr$^{1-/-}$ n=11 voles). **b**, Mean z-scored ΔF/F traces at the onset of social bouts (WT n=782 traces, Oxtr$^{1-/-}$ n=640 traces). **c**, Peak z-scored ΔF/F values. **d**, AUC values of z-scored ΔF/F traces. **e**, Percent of timed mating assay time spent engaged in non-AG sniffing, AG sniffing, or side-by-side contact. **f**, Proportions of males that exhibited mating behavior. **g**, Left, percent of assay time spent engaged in mating behavior, plotting only the males that exhibited any mating. Right, number of mating bouts, plotting only males that exhibited any mating (WT n=7, Oxtr$^{1-/-}$ n=6 voles). **h**, Mean z-scored ΔF/F traces at the onset of mounts that transition to intromission. The red line indicates time points at which mean z-scored ΔF/F differs between WT and Oxtr$^{1-/-}$ males. The orange asterisks on the mean PETH lines indicate time points at which that mean z-scored ΔF/F

is significantly different from 0 ΔF/F (WT n=23 traces/3 voles, Oxtr$^{1-/-}$ n=13 traces/2 voles). **i**, Peak z-scored ΔF/F values. **j**, AUC values of z-scored ΔF/F traces. **k**, Mean z-scored ΔF/F traces at the onset of mounts that do not transition to intromission (WT n=157 traces/7 voles, Oxtr$^{1-/-}$ n=42 traces/6 voles). **l**, Peak z-scored ΔF/F values. **m**, AUC values of z-scored ΔF/F traces. Detailed statistics are presented in Extended Data File 1. *p<0.05, **p<0.01, ****p<0.0001. WT, wild-type; AUC, area under the curve; AG, anogenital. Vole images were created in BioRender. Manoli, D. (2026) https://BioRender.com/00l1i4c. Source data are provided as a Source Data file.

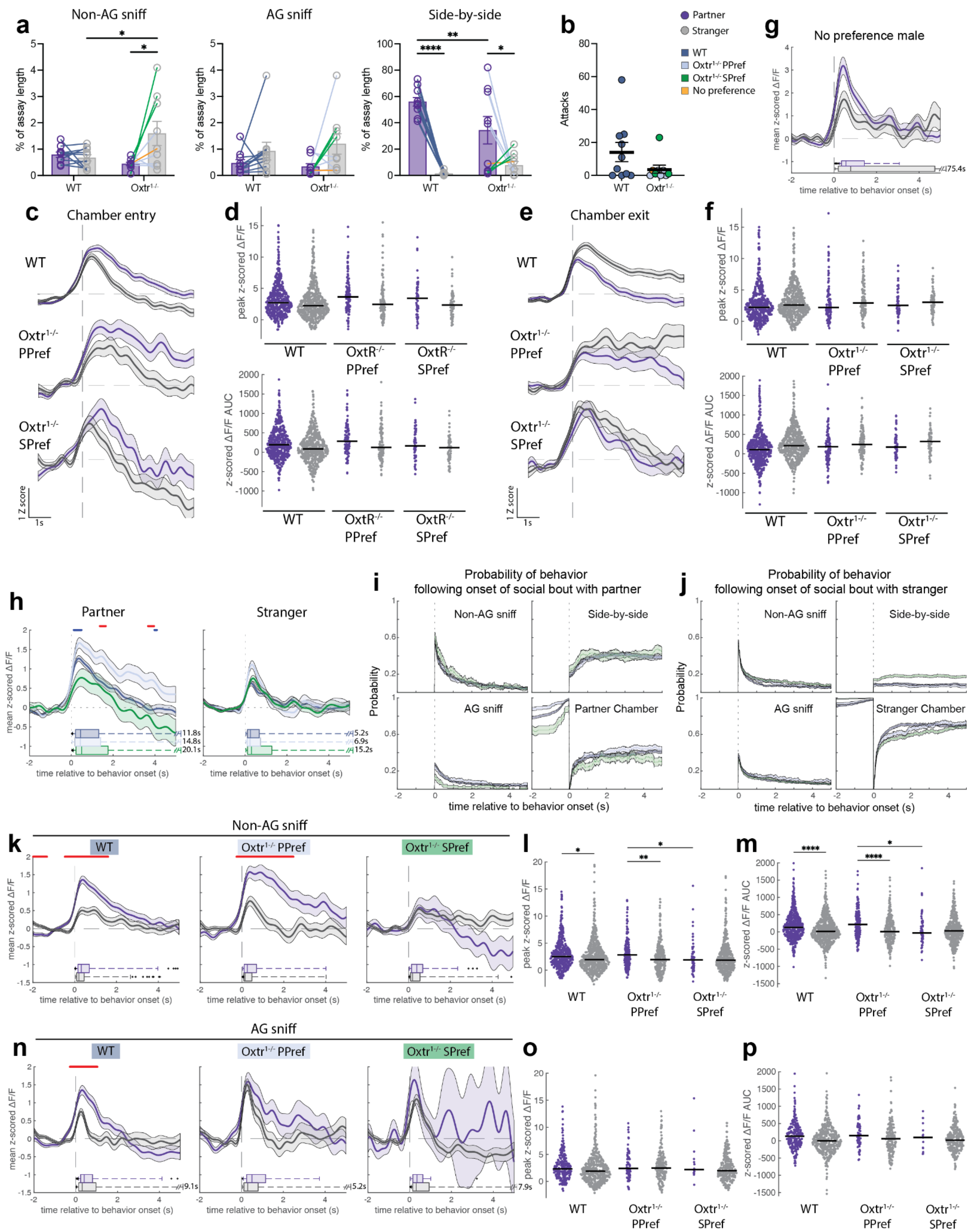

Supplementary Figure 9: Additional data from male PPTs. a, Percent of PPT assay time spent engaged in non-anogenital (AG) sniffing, AG sniffing (significant main effect of stimulus animal),

or side-by-side contact. Note the difference in y-axis for side-by-side contact (WT n=10, $Oxtr^{1-/-}$ n=9 voles). **b**, Male attacks against the stranger female. **c**, Mean ΔF/F traces surrounding entries to either the partner chamber or stranger chamber (for this and subsequent plots: WT n=10 voles, $Oxtr^{1-/-}$ PPref n=5 voles, $Oxtr^{1-/-}$ SPref n=3 voles; $WT_{Partner}$ n=485, $WT_{Stranger}$ n=542; $Oxtr^{1-/-}$ $PPref_{Partner}$ n=107, $Oxtr^{1-/-}$ $PPref_{Stranger}$ n=108; $Oxtr^{1-/-}$ $SPref_{Partner}$ n=56, $Oxtr^{1-/-}$ $SPref_{Stranger}$ n=62 traces). **d**, Top, peak z-scored ΔF/F values with median value overlaid (significant main effect of stimulus animal chamber). Bottom, AUC values from z-scored ΔF/F traces (significant main effect of stimulus animal chamber). **e**, Mean ΔF/F traces surrounding exits from either the partner chamber or stranger chamber ($WT_{Partner}$ n=490, $WT_{Stranger}$ n=563; $Oxtr^{1-/-}$ $PPref_{Partner}$ n=105, $Oxtr^{1-/-}$ $PPref_{Stranger}$ n=116; $Oxtr^{1-/-}$ $SPref_{Partner}$ n=51, $Oxtr^{1-/-}$ $SPref_{Stranger}$ n=64 traces). **f**, Top, peak z-scored ΔF/F values (significant main effect of stimulus animal chamber). Bottom, AUC values from z-scored ΔF/F traces (significant main effect of stimulus animal chamber). **g**, Mean ΔF/F traces from the male exhibiting no partner or stranger preference surrounding social bouts with the partner (purple) or stranger (gray). At the base of the plot is an adjusted boxplot of durations of the initiating behaviors (partner n=41, stranger n=56 traces from 1 male). **h**, Mean z-scored ΔF/F traces at the onset of social bouts with a partner (left) or stranger (right). Data are the same as those plotted in Fig. 3k. Red bars above the plot indicate time points at which mean z-scored ΔF/F significantly differs between WT and partner-preferring $Oxtr^{1-/-}$ males. Blue bars indicate time points at which partner-preferring and stranger-preferring $Oxtr^{1-/-}$ males differ ($WT_{Partner}$ n=932 traces, $Oxtr^{1-/-}$ $PPref_{Partner}$ n=296 traces, $Oxtr^{1-/-}$ $SPref_{Partner}$ n=111 traces; $WT_{Stranger}$ n=1193 traces, $Oxtr^{1-/-}$ $PPref_{Stranger}$ n=500 traces, $Oxtr^{1-/-}$ $SPref_{Stranger}$ n=821 traces). **i**, Probability of social touch behaviors following the onset of a social bout with a partner. Data are related to those shown in h (Partner). **j**, Probability of social touch behaviors following the onset of a social bout with a stranger. Data are related to those shown in h (Stranger). **k**, Mean z-scored ΔF/F traces at the

onset of non-AG sniffs of the partner or stranger. The red line indicates time points at which mean z-scored ΔF/F differs between partner and stranger-related activity ($WT_{Partner}$ n=508 traces, $WT_{Stranger}$ n=647 traces; $Oxtr^{1-/-}$ $PPref_{Partner}$ n=166 traces, $Oxtr^{1-/-}$ $PPref_{Stranger}$ n=277 traces; $Oxtr^{1-/-}$ $SPref_{Partner}$ n=68 traces, $Oxtr^{1-/-}$ $SPref_{Stranger}$ n=434 traces). **l**, Peak z-scored ΔF/F values. **m**, AUC values of z-scored ΔF/F traces. **n**, Mean z-scored ΔF/F traces at the onset of social bouts initiated by AG sniffing ($WT_{Partner}$ n=252 traces, $WT_{Stranger}$ n=434 traces; $Oxtr^{1-/-}$ $PPref_{Partner}$ n=71 traces, $Oxtr^{1-/-}$ $PPref_{Stranger}$ n=185 traces; $Oxtr^{1-/-}$ $SPref_{Partner}$ n=15 traces, $Oxtr^{1-/-}$ $SPref_{Stranger}$ n=268 traces). **o**, Peak z-scored ΔF/F values. **p**, AUC values of z-scored ΔF/F traces (significant main effect of stimulus animal). Detailed statistics are presented in Extended Data File 1. *$p<0.05$, **$p<0.01$, ***$p<0.001$, ****$p<0.0001$. AG, anogenital; WT, wild-type; PPref, $Oxtr^{1-/-}$ partner-preferring; SPref, $Oxtr^{1-/-}$ stranger-preferring; AUC, area under the curve. Source data are provided as a Source Data file.

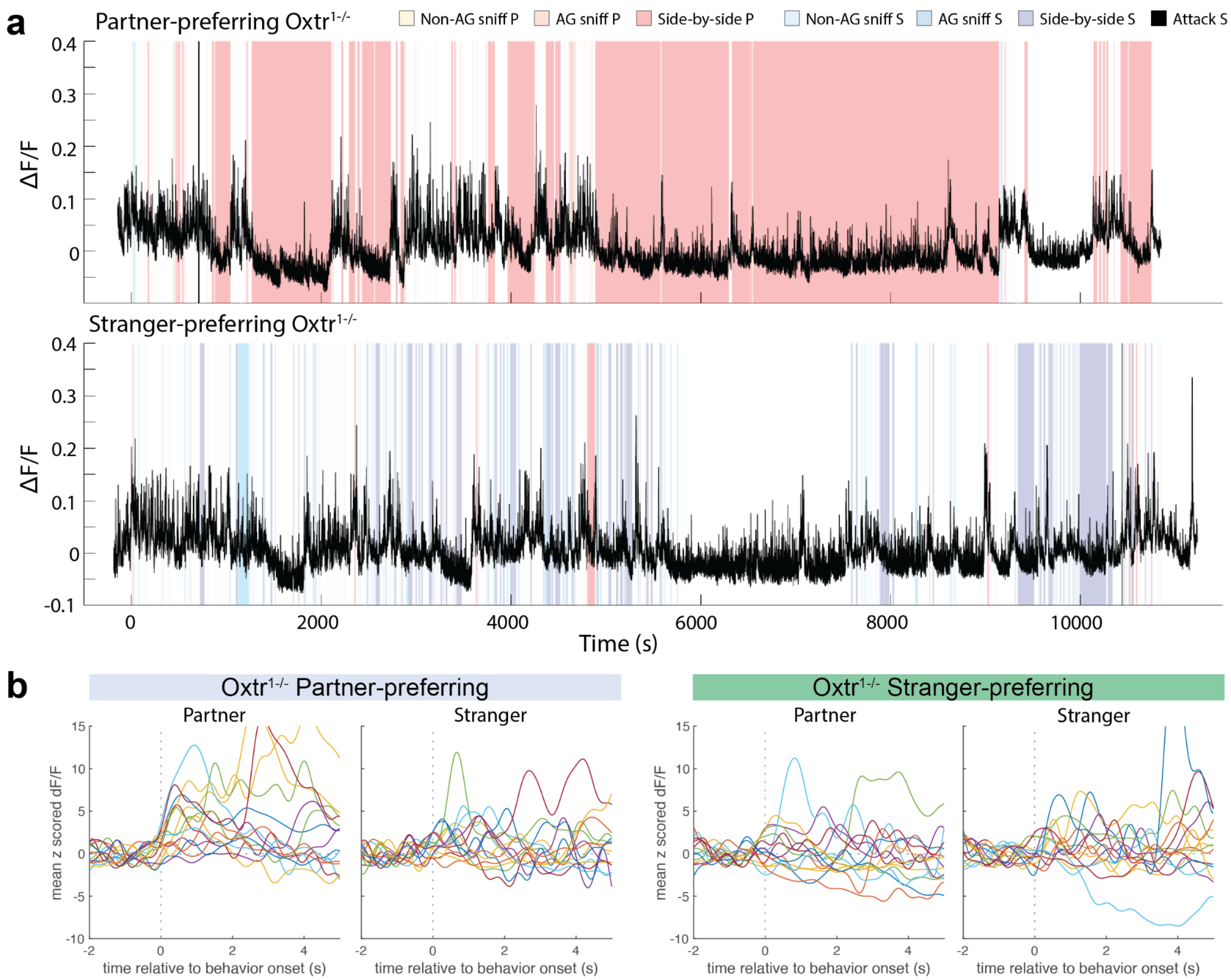

Supplementary Figure 10: Example raw data from partner- and stranger-preferring Oxtr$^{1-/-}$ male partner preference tests. a, Examples of whole partner preference test NAc GCaMP6m traces with social behavior overlaid. Top: Partner-preferring Oxtr$^{1-/-}$ male. Bottom: Stranger-preferring Oxtr$^{1-/-}$ male. **b**, Fifteen randomly sampled examples of z scored ΔF/F at the onset of social bouts of behavior with either the partner or stranger in Oxtr$^{1-/-}$ partner-preferring males (left) and Oxtr$^{1-/-}$ stranger-preferring males (right).

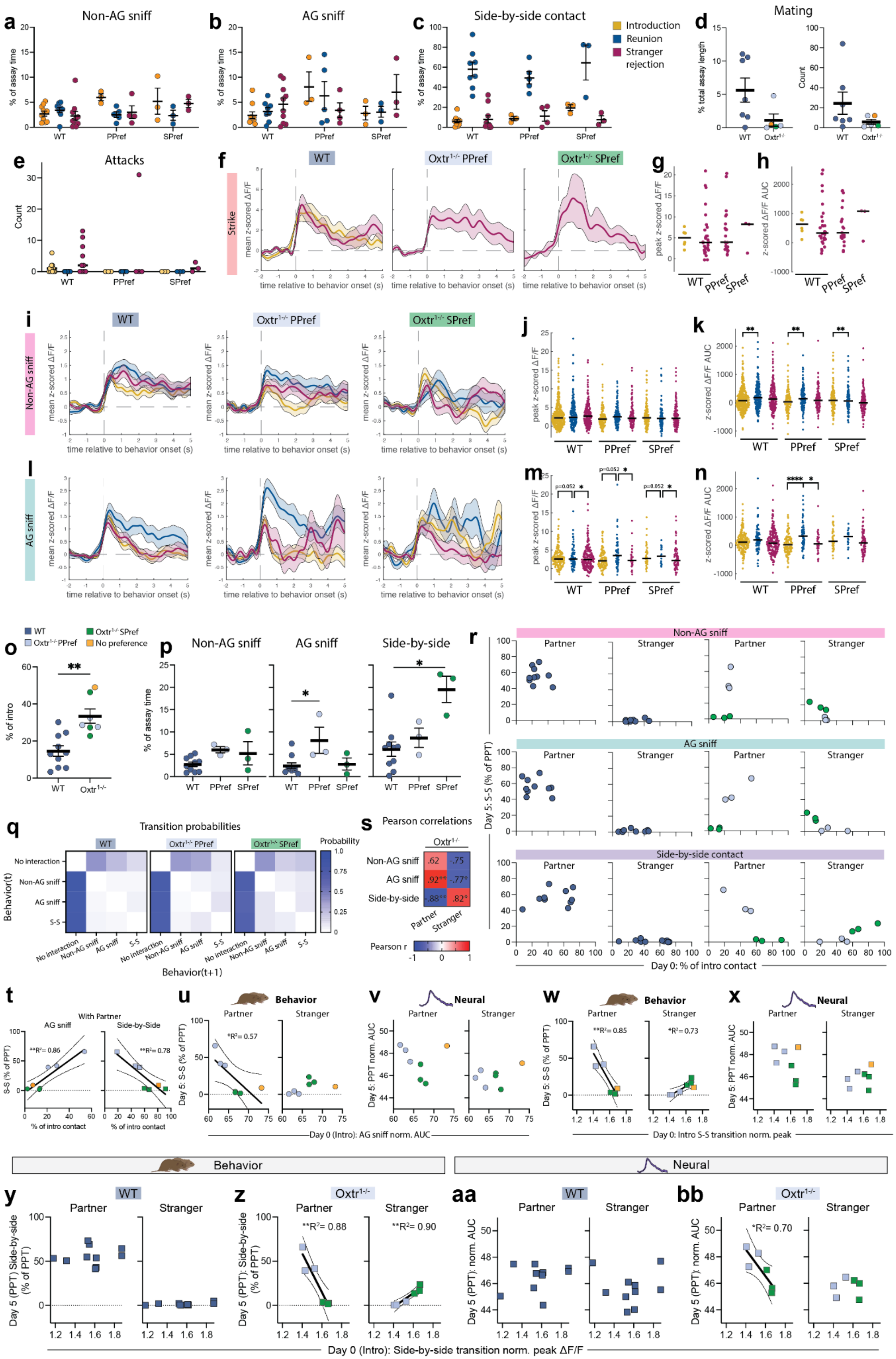

a
Non-AG sniff
b
AG sniff
c
Side-by-side contact
Introduction
Reunion
Stranger rejection
d
Mating
% of assay time
WT
PPref
SPref
% total assay length
Count
e
Attacks
f
Oxtr-/- PPref
Oxtr-/- SPref
Strike
mean z-scored ΔF/F
time relative to behavior onset (s)
g
peak z-scored ΔF/F
h
z-scored ΔF/F AUC
i
Non-AG sniff
j
k
l
AG sniff
m
n
o
% of intro
WT
Oxtr-/- PPref
Oxtr-/- SPref
No preference
p
Side-by-side
q
Transition probabilities
Probability
No interaction
Non-AG sniff
AG sniff
S-S
Behavior(t)
Behavior(t+1)
r
Partner
Stranger
Day 5: S-S (% of PPT)
Side-by-side contact
Day 0: % of intro contact
s
Pearson correlations
Oxtr-/-
Pearson r
t
With Partner
S-S (% of PPT)
% of intro contact
u
Behavior
*R²= 0.57
Day 5: S-S (% of PPT)
v
Neural
Day 5: PPT norm. AUC
Day 0 (Intro): AG sniff norm. AUC
w
**R²= 0.85
*R²= 0.73
x
Day 0: Intro S-S transition norm. peak
Behavior
Neural
y
z
**R²= 0.88
**R²= 0.90
Day 5 (PPT) Side-by-side (% of PPT)
aa
bb
*R²= 0.70
Day 5 (PPT): norm. AUC
Day 0 (Intro): Side-by-side transition norm. peak ΔF/F

Supplementary Figure 11: Additional data related to Figure 4. a, Percent of assay time spent non-AG sniffing the stimulus animal across assays (for subsequent plots unless otherwise noted, WT_{Intro} n=10, $WT_{Reunion}$ n=8, $WT_{Stranger}$ n=10, $Oxtr^{1-/-}$ $PPref_{Intro}$ n=3, $Oxtr^{1-/-}$ $PPref_{Reunion}$ n=5, $Oxtr^{1-/-}$ $PPref_{Stranger}$ n=4, $Oxtr^{1-/-}$ $SPref_{Intro}$ n=3, $Oxtr^{1-/-}$ $SPref_{Reunion}$ n=3, $Oxtr^{1-/-}$ $SPref_{Stranger}$ n=3). **b**, Percent of time spent AG sniffing the stimulus animal across assays. **c**, Percent of time spent in side-by-side contact with the stimulus animal across assays (significant main effect of assay). **d**, Left, percent of assay time spent engaged in mating behavior, plotting only the males that exhibited any mating. Right, number of mating bouts, plotting only males that exhibited any mating (WT n=7, $Oxtr^{1-/-}$ n=5 voles). **e**, Number of strikes in the pairing, reunion, and stranger rejection assays. **f**, Mean ΔF/F traces surrounding strikes (WT Intro n=6 traces from 5 animals; WT Stranger rejection n=30 traces from 6 animals; $Oxtr^{1-/-}$ PPref Stranger rejection n=22 traces from 1 animal; $Oxtr^{1-/-}$ SPref Stranger rejection n=3 traces from 2 animals). **g**, Peak ΔF/F values. **h**, AUC values from z-scored ΔF/F traces. **i**, Mean z-scored ΔF/F traces surrounding non-AG sniffs during the introduction, reunion, and stranger rejection assays. Yellow, intro (WT n=338 traces; $Oxtr^{1-/-}$ PPref n=137 traces; $Oxtr^{1-/-}$ SPref n=123 traces); blue, reunion (WT n=193 traces; $Oxtr^{1-/-}$ PPref n=124 traces; $Oxtr^{1-/-}$ SPref n=87 traces); purple, stranger rejection (WT n=220 traces; $Oxtr^{1-/-}$ PPref n=115 traces; $Oxtr^{1-/-}$ SPref n=130 traces). **j**, Peak z-scored ΔF/F values with median value overlaid (significant main effect of group). **k**, AUC values from z-scored ΔF/F traces (significant main effects of group and assay). **l**, Mean ΔF/F traces surrounding AG sniffs during the intro, reunion, and stranger rejection assays. (Intro: WT n=187 traces; $Oxtr^{1-/-}$ PPref n=131 traces; $Oxtr^{1-/-}$ SPref n=42 traces; Reunion WT n=77 traces from 7 animals; $Oxtr^{1-/-}$ PPref n=65 traces from 4 animals; $Oxtr^{1-/-}$ SPref n=20 traces from 3 animals; Stranger rejection: WT n=215 traces; $Oxtr^{1-/-}$ PPref n=34 traces; $Oxtr^{1-/-}$ SPref n=81 traces). **m**, Peak ΔF/F values (significant main effect of assay). **n**, AUC values from z-scored ΔF/F traces. **o**, Percent of time engaged in social interaction

with a newly partnered female during the introduction, plotting only animals from which we successfully collected both intro and PPT data (including the single $Oxtr^{1-/-}$ male with no preference). Individual animals with median overlaid and colored according to PPT behavior profile (WT n=10, $Oxtr^{1-/-}$ n=7). **p**, Percent of introduction assay time spent engaged in non-anogenital (AG) sniffing, AG sniffing, or side-by-side contact. **q**, Heat maps of transition probabilities from Markov modeling of behavior. **r**, Correlation plots between behavior exhibited during the intro and side-by-side contact with either the partner or stranger during the PPT (X-axis, percent of social contact; Y-axis, percent of PPT time). These plots are the raw data of the heat maps shown in Fig. 4d. **s**, Heat map of correlations between behavior exhibited during the intro and side-by-side contact with either the partner or stranger during the PPT. Data are related to Fig. 4d, but include the single animal with no huddling preference. **t-x**, Plots related to Fig. 4e,m,o,v,x but including the single $Oxtr^{1-/-}$ male with no huddle preference. **t**, Linear regression of intro behavior to PPT behavior. X-axis: Percent of social touch during the introduction spent AG sniffing (left, teal shading) or in side-by-side contact (right, purple shading). Y-axis: Percent of time spent in side-by-side contact (S-S) with the partner during the PPT. **u-v**, Linear regressions of mean normalized AUC at the onset of AG sniff bouts, averaged by animal, during the introduction and PPT side-by-side contact (g) or PPT mean normalized AUC surrounding social bouts by animal (h). **w-x**, Linear regressions of normalized peak ΔF/F surrounding sniff to side-by-side contact transitions during the introduction and PPT side-by-side contact (h) or normalized AUC surrounding social bouts (i). **y-bb**, Linear regressions comparing side-by-side-related neural activity and PPT behavior or neural data. X-axis: normalized peak ΔF/F at the onset of sniff to side-by-side contact transitions during the introduction, averaged by animal. Y-axis: PPT side-by-side contact (y-z) or PPT normalized AUC surrounding social bouts, averaged by animal (aa-bb). Detailed statistics are presented in Extended Data File 1. *p<0.05, **p<0.01, ***p<0.001,

****p<0.0001. WT, wild-type; AG, anogenital; PPref, partner-preferring $Oxtr^{1-/-}$ male; SPref, stranger-preferring $Oxtr^{1-/-}$ male; PPT, partner preference test; AUC, area under the curve. Source data are provided as a Source Data file.

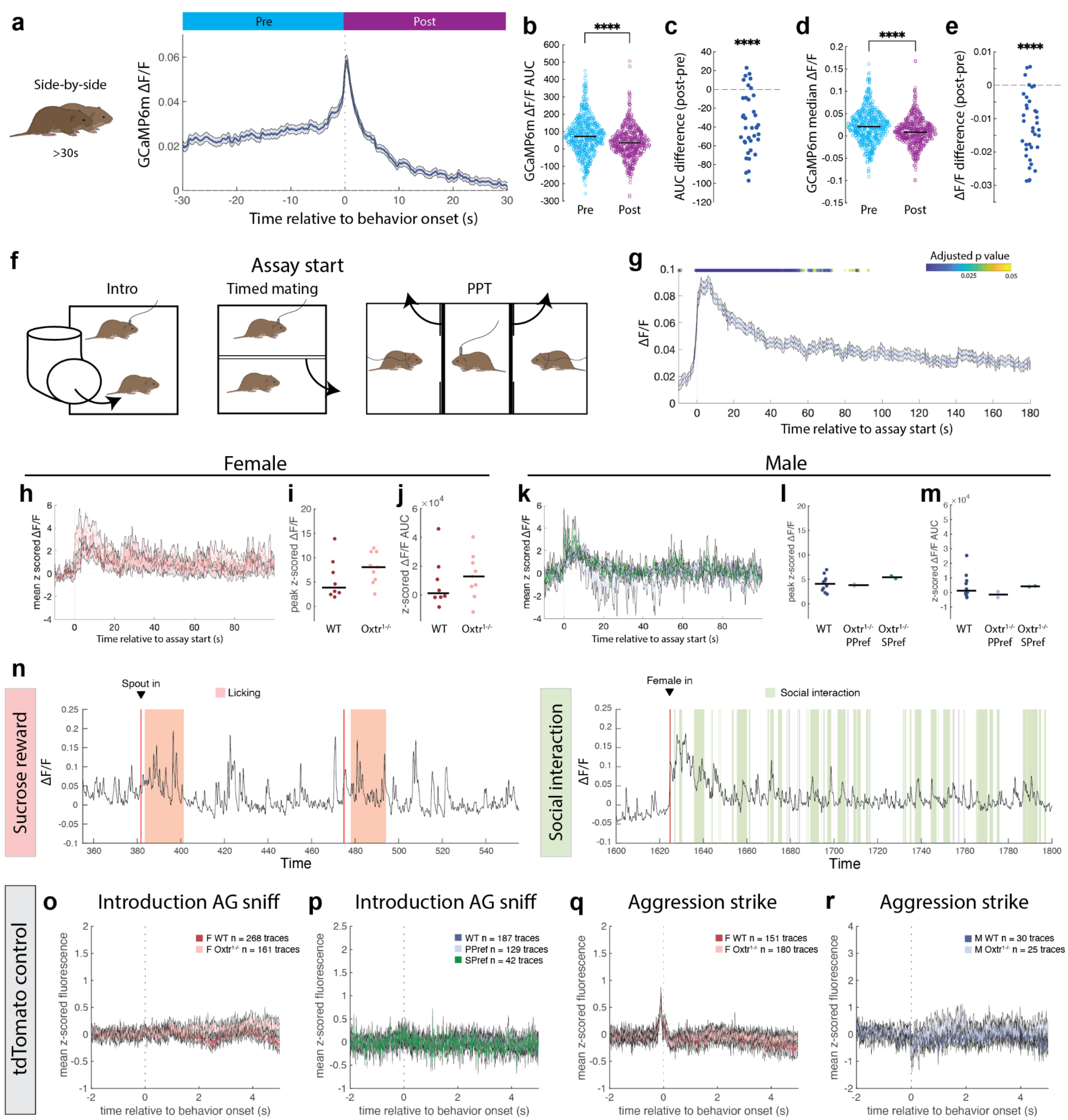

Supplementary Figure 12: Dynamics of NAc calcium activity during periods of rest and at assay start. a, GCaMP6m ΔF/F traces were extracted surrounding bouts of side-by-side contact lasting 30 seconds or more (prolonged huddling), during which time most animals rested or slept. Plotted is the mean of GCaMP6m ΔF/F traces (n=560 traces from 37 animals). We compared area under the curve (AUC) and median ΔF/F values from the pre-huddle period (blue, -30 to 0 seconds) to the post-huddle period (purple, 0 to 30 seconds). **b**, GCaMP6m ΔF/F AUC from the pre- versus post-huddle period. **c**, Individual animal difference between median pre-huddle AUC and post-huddle AUC. **d**, Median GCaMP6m ΔF/F from the pre- versus post-huddle period. **e**, Individual animal difference between median pre-huddle ΔF/F and post-huddle ΔF/F. **f**, For assays conducted in the home cage, the stimulus animal was transferred to the cage. “Assay start” was defined as the moment the stimulus was placed into the cage. Note: For timed matings and PPTs, assay start was defined as the moment the barriers between animals were removed. **g**, Mean of GCaMP6m ΔF/F traces from all animals and all assays at the time of assay start (n=192). Asterisks above the plot are time points where mean ΔF/F is significantly different from the pre-assay (-10s to 0s) mean ΔF/F value of 0.0237 (Benjamini-Yekutieli correction applied to the resulting p values). **h-m**, Assay start mean ΔF/F (h,k) and metrics from the introduction assay (h: WT n=8, $Oxtr^{1-/-}$ n=9; k: WT n=10, $Oxtr^{1-/-}$ PPref n=2; $Oxtr^{1-/-}$ SPref n=2). Traces were z scored to the -10 to 0 second period, and peak ΔF/F and AUC were calculated from the period between 0 to 100 seconds. (i) and (l): Peak z-scored ΔF/F values. (j) and (m): AUC of ΔF/F traces. **n**, GCaMP6m ΔF/F trace from a male prairie vole during consumption of a 10% sucrose reward (left, first 2 trials) and social interaction with an opposite-sex conspecific (right). **o-p**, Control tdTomato signal (plotted as mean +/- s.e.m.) at the onset of anogenital (AG) sniff bouts during the introduction in females (o: WT n=268 traces/8 voles, $Oxtr^{1-/-}$ n=161 traces/9 voles) and males (p: WT n=187 traces/11 voles, $Oxtr^{1-/-}$ PPref n=129 traces/5 voles; $Oxtr^{1-/-}$ SPref n=42 traces/3 voles).

q-r, Control tdTomato signal at the onset of strikes during the stranger rejection assay in females (q: WT n=151 traces/8 voles, $Oxtr^{1-/-}$ n=180 traces/11 voles) and males (r: WT n=30 traces/6 voles, $Oxtr^{1-/-}$ n=25 traces/3 voles). Notably, there was a consistent increase in tdTomato signal immediately prior to strikes in females but no fluctuations following strike onset. Detailed statistics are presented in Extended Data File 1. ****p<0.0001. AUC, area under the curve; AG, anogenital; WT, wild-type. Vole images were created in BioRender. Manoli, D. (2026) https://BioRender.com/00l1i4c. Source data are provided as a Source Data file.
