# Supplementary material for: Loss of oxytocin receptor function disrupts neural signatures of pair bonding and fidelity in the nucleus accumbens
Source: Nat Commun. 2026 Sep 12;17:10755. doi: 10.1038/s41467-026-76876-8 (PMC13661945; doi:10.1038/s41467-026-76876-8)
Supplement: Supplementary file 2 [file 41467_2026_76876_MOESM2_ESM.pdf]

nature portfolio

# Peer Review File

# Loss of oxytocin receptor function disrupts neural signatures of pair bonding and fidelity in the nucleus accumbens

Corresponding Author: Dr Devanand Manoli

**This file contains all reviewer reports in order by version, followed by all author rebuttals in order by version.**

Version 0:

Reviewer comments:

Reviewer #1

(Remarks to the Author)
In this manuscript, the author show that a developmental loss oxytocin receptor (Oxtr) signaling alters behavioral and neuronal processes that precede the formation of pair bonds in monogamous prairie voles. These are critically important findings that follow up on the initial publications of Oxtr -/- prairie vole papers, which showed that knockouts could form pair bonds. This has created an impression by some in the field that Oxtr is not important for pair bond formation despite decades of evidence that point to the contrary. The authors take a big step towards resolving this paradox by showing that Oxtr -/- differ from wild-type voles in the early stages of pair bond formation. Developmental loss of Oxtr alters the reactivity of the nucleus accumbens, a key node modulating pair bond formation. In my opinion, this manuscript will be viewed as more important than the previous knockout vole papers because it provides a potential explanation for resolving older data with the newer knockout results. That said, the presentation of the results is confusing in places and could be revised to better highlight the importance of the results.

Major comments

-In the abstract and discussion I think the authors need to highlight the fact that they find effects of genotypes on processes that affect the formation of pair bonds and that this evidence possibly provides an explanation for apparently divergent results between KO and pharmacology. In a more naturalistic setting, where individuals might only interact for a few minutes (versus the forced pairing that is normally done in neuroscience studies) these effects could be fundamental to altering behavior.

-It appears that analyses in males and females were conducted separately and this may be why they are not directly compared. I am fine with that, but it is confusing when the authors say that different responses in the same behavioral or neural response and these data are in different figures. I think the manuscript would flow better if the authors discussed each part of the pair bond formation (introduction, mating etc) in order and present male and female data in each section. Even if the male and female data were kept in separate figures it would be much easier to keep track of the different findings as opposed to having to remember what was found 3 figures previously.
- Extended data 3 was confusing. It seems that behavior conducted on different days was being compared. Also that statement that WT and Oxtr-/- did not differ in calcium responses doesn't match the results in panel 1 and j. Why does line 145 refer back to Extended Fig 3a again?

-Why not conduct analysis on calcium transient before mounting given the NAc role in motivation (2f suggests possible difference before mating?). Similarly, is it known if females can pace mating bouts? Work from Jill Becker shows this is key for modulation of dopamine release.
Again in Fig 2 authors state that NAc responses were similar btw WT and -/- yet there are significant differences between partner and stranger in the -/- voles but not wildtype. Perhaps this is because the weaker responses to strangers in the -/- voles does not fit with other results should reduced "pro-social" behavior in the -/-. However, the GCaMP is expressed of a hSyn promoter and different populations of neurons could be responding in different contexts. Luo et al 2024 (Neuropsychopharm) found increased GCaMP responses in NAc during bouts of avoidance.
-
In figure 4k, do stranger preferring voles differ from wt? If not, is that a surprise?

Minor comments

- In lines 18-19, the authors state that the way social attachments and specific social behaviors in ethological contexts form "remains unknown," though they go on to cite several papers that describe mechanisms underlying these behaviors throughout their paper. This statement isn't accurate and should probably be replaced with something more accurate, such as "aspects of these mechanisms remain understudied."
- The exclusion rate is relatively high, with about 40% of subjects being excluded. It would be helpful to know exactly how many animals were excluded due to the different reasons listed (poor signal, death, ataxia, etc.).
- The exclusion of animals with less robust signal (< 5%) could contribute to an overrepresentation of animals with strong signal in the data.
- Some of the statistical analysis relied on transformed data (Box-Cox, log transforms), but lack normality tests and other tests for other assumptions (heterogeneity, etc.).
- Side-by-side contact is described as at least 50% of body contact between voles, though no time threshold is described. Does the contact need to last a specific amount of time or were bouts of this contact recorded instead?
- The Markov chains analyses are a great tool for considering behavioral processes involved in pair bond formation. Was this done for the males also?
- In line 26, authors state that "prosocial behavior in females in a state-dependent manner…" Prior to this statement, the exact "states" are not described. Changing this to "bonding state" or something similar would add clarity to this sentence.
- On line 155-156 the authors should state how long the observation period was for mating to be observed.

Reviewer #2

(Remarks to the Author)
In "Oxytocin receptor function regulates neural signatures of pair bonding and fidelity in the nucleus accumbens" Long et al correlate neural activity with social behavior during pair bonding interactions. The vole has been an excellent model of prosocial behavior and the creation of the Oxtr-/- line has made an impactful advance in altering understanding of the function of neural oxytocin. This study aims to further characterize and provide a neural basis for how pair bonds are formed. The findings are nuanced and complicated. They analyze either male or female NAc activity during various social interactions across a 10 day period that include partner introduction, several partner preference tests, mating, stranger rejection, and partner separation/reunification. This is an excellent assay design that allows the role of neural activity to be comprehensively analyzed in almost every social permutation. Remarkably, no differences were noted in any social situation except in the duration of initial anogenital investigation of strangers. During novel social activity, oxytocin and the corresponding neural response in the NAc has opposite effect on the sexes promoting investigation in females and inhibiting in males. Moreover, they find a subset of male mutants do not form long term partner preference which can be identified by reduced neural activity in the NAc. There are no neural manipulations to test and provide mechanistic insight, nor is there an advance in understanding the potential sexual dimorphism. The clearest phenotype was in a sub-population of male mutants that continue to show interest in strangers, but the understanding is limited to a correlative readout which is really no more mechanistically informative than the behavioral readout. While I think it is very interesting and important to report the lack of importance of Oxyr in most of the behaviors observed here, I don't think the minor differences reported are critically investigated at this stage to warrant the title and abstract's conclusion. Either, adjust the conclusions to align with the data or experimentally validate to ensure the data supports the new assumptions.

Concerns:
1) While the authors show cannula placement in the individuals, (and it looks like there is a dorsal bias of sp males) there is no characterization of GCaMP expression. It is expected that expression levels will differ which may account for all of the neural activity differences observed. Cannula placement (in females, pp and sp males) shown with the extent and location of viral expression in each individual will help to better interpret the results. As it stands, most of the neural activity differences are peak-differences (latency to respond and slope of response are largely similar) suggesting that where differences are observed it is due to total amount of fluorescence to be captured rather than differences in neural dynamics. This is consistent with both cannula placement and viral expression variability.
2) Please expand and explain the role of the observed behavioral differences on the social structure. It appears that any changes in anogenital investigation do not have subsequent effects on pair-bonding or any other metrics of social behavior. If it is not important to the process, then it is an irrelevant part of overall social structure.
3) Please include more raw data of the neural activity during behavior (as supplemental). Where it is shown (Fig. 1d and S9) it does not seem to correlate strongly with the observed behavior. Does a single animal's neural response differ during anogenital investigation (can you see it in the raw data)? Can you see the difference between pp and sp males in the raw data?
4) Please expand and explain the role of the NAc activity in driving pair-bonding in the social circuit based on your new findings. A toy model would be helpful to better understand how the new findings impact understanding circuits that drive pro-social behavior.
5) Almost all of my concerns (above) could be mitigated by including standard chemogenetic experiments to manipulate neural activity (enhance or impair) and confirm that it alters behavior as predicted by the correlations observed. With the success of the fiber photometry here, it does not seem technically challenging to include such important validations.

Reviewer #3

(Remarks to the Author)
This study investigated how oxytocin receptor (Oxtr) function affects pair bonding behaviors and neural activity in prairie voles. The researchers used fiber photometry to measure real-time neural activity in the nucleus accumbens while male and female prairie voles, both with and without Oxtr, interacted with potential mates. They found that Oxtr has sex-specific effects. In females, Oxtr regulates initial social investigation of novel males. In males lacking Oxtr, it could be predicted whether an individual would eventually prefer their partner or a stranger based on their neural activity patterns during their very first interaction. This research is important and advances our understanding of Oxtr mechanisms in prairie vole social behavior. However, there are several considerations worth discussing:

•The findings in females describe both behavioral changes (e.g., reduced investigative and affiliative interactions in Oxtr1-/-) and neural activity changes (e.g., decreased NAc activity during anogenital investigations). However, the connection between these two sets of findings is not fully explored. Does reduced NAc activity directly cause the behavioral changes, or are they parallel effects of Oxtr loss?

•Since oxytocin and vasopressin can bind to each other's receptors, does the absence of oxytocin receptors potentially disrupt vasopressin signaling pathways in other brain regions?

•More detail on how the authors define affiliative behavior should be in the beginning of results or in their table.

•The authors report sexual behavior and aggression inconsistently across experiments. If the behavior was not observed in certain experiments, it should be explicitly stated.

•Sex difference comparisons are confusing since the male and female data are presented separately. Why were the sexes not compared statistically? This makes it difficult to interpret any kind of sex difference in the data sets.

•In females, was sexual behavior affected during the 6 hours of cohabitation, or did the authors notice any differences in latency to receive mounting or intromissions in early pairings?

•While the study shows that Oxtr loss in females reduces both nucleus accumbens activity and social investigation of novel males during early bonding, the discussion should better explain the potential mechanisms underlying these disruptions.

•The observed behavioral changes in Oxtr1-/- prairie voles could result from factors like altered sensory processing, reduced motivation, or compensatory mechanisms, rather than direct effects of Oxtr loss. In females, the lack of differences in NAc activity during non-anogenital sniffs or side-by-side contact suggests that Oxtr's role may be limited to specific social behaviors rather than broadly influencing prosociality.

•In the male data, the sample sizes are small (subgroups of 5 and 3 voles), making it difficult to draw robust conclusions about distinct behavioral subpopulations. Statistical limitations should be discussed. The authors say that the loss of Oxtr in only 3 non-partner-preferring males revealed a population that showed no difference in NAc activity during interactions with partners versus strangers. Is it typical for all WT animals to form partner preferences? The observed behavior in these 3 males could simply reflect normal variation rather than a meaningful subpopulation.

•It may be difficult to correlate the partner-stranger AUC differences if the WT males are already at a ceiling (strong preference for partners) or floor (minimal interaction with strangers), leaving little room for variation to detect meaningful relationships.

Version 1:

Reviewer comments:

Reviewer #1

(Remarks to the Author)
This is a very strong revision. All of my comments have been addressed well by the authors.

Reviewer #2

(Remarks to the Author)
While there have been some changes that make the paper slightly easier to read and understand, my overall concerns remain. The title and abstract still claim a novel and mechanistic relationship between NaC activity, behavior, and Oxtr. The paper does not support these claims. The authors are still unable to make that relationship clear (either by a model or a clear textual conclusion) in a way that is useful as a base for the neuroscience community to follow up upon or for a general reader to determine the extent to which oxytocin can be thought of as a 'love drug'. I am not suggesting that nuanced results are not interesting, but here we are served a buffet of observations without much synthesis or understanding of how these data advances our knowledge of how this neuromodulator works (either in the brain or behaviorally). Moreover, the most potentially interesting part of this study is the stranger preference displayed by the mutant males. But the robustness of these conclusions are questionable because the number of males falling into this category is so small (N=3), leaving also a very small partner preference male group (N=4). I am disappointed that the authors did not supplement this finding with another

cohort of data. Finally, it was helpful for me as a reader to see additional ethograms of the raw data of neural activity and behavior (figure 1d and extended figure 3) however, even in these best case examples the relationship between the neural activity and the actual behavior is not correlated in a way that suggests a relationship except the correlation occurring during the initial sniff bout – which is not really the focus of this study. I believe that the conclusions need to be softened to better align with the data, and the author's understanding of the role of this data to impact our current model of the function of oxtr be spelled out. As it stands it is an incremental advance.

Reviewer #3

(Remarks to the Author)
The revised manuscript represents an improvement that addresses a lot of my concerns. However, given the acknowledged limitations of fiber photometry and the inability to establish whether NAc activity changes drive behavioral outcomes or are simply parallel consequences of Oxtr deletion, I recommend the authors adjust their language to better reflect the correlational nature of their findings (e.g., Oxtr "regulates" neural processing imply causal mechanisms that cannot be established).

Version 2:

Reviewer comments:

Reviewer #2

(Remarks to the Author)
The interpretation and synthesis of the data are slightly more flushed out in the current manuscript. The new figure 5, though complicated to follow without reading the text, is a helpful summary. While the interpretations are largely based on correlative results, the data itself is rigorously presented so others can evaluate the conclusion and impact. I expect publication of the study in Nature Communications will now provide a platform for the authors and others to continue flushing out mechanistic understanding of oxytocin's role in prosocial behavior.

**Open Access** This Peer Review File is licensed under a Creative Commons Attribution 4.0 International License, which permits use, sharing, adaptation, distribution and reproduction in any medium or format, as long as you give appropriate credit to the original author(s) and the source, provide a link to the Creative Commons license, and indicate if changes were made.
In cases where reviewers are anonymous, credit should be given to 'Anonymous Referee' and the source.
The images or other third party material in this Peer Review File are included in the article's Creative Commons license, unless indicated otherwise in a credit line to the material. If material is not included in the article's Creative Commons license and your intended use is not permitted by statutory regulation or exceeds the permitted use, you will need to obtain permission directly from the copyright holder.
To view a copy of this license, visit https://creativecommons.org/licenses/by/4.0/

## RESPONSES TO REVIEWER COMMENTS

We would like to thank the reviewers for their thoughtful and constructive feedback. We have made extensive revisions to improve the clarity, rigor, and interpretability of our manuscript. We reorganized the presentation of results to improve clarity and show statistical comparisons between sexes during the initial introduction of a vole to its partner. We revised the abstract and discussion to highlight how our findings may reconcile previously conflicting results from Oxtr knockout and pharmacology studies. We clarified multiple figure panels, especially Extended Data Figure 5, to better delineate axes of comparison and the timeline of behavioral assays. We addressed concerns about the interpretation of the neural data by adding more raw fiber photometry traces and new Extended Data Figures showing viral expression and cannula placement for each individual animal. We clarified statistical approaches, exclusions, and behavioral definitions, and added details regarding statistical methods in the methods section and supplemental data. To address questions about the potential mechanisms driving the changes in behavior and neural activity that we observed in Oxtr mutant animals, we significantly revised our discussion to enumerate potential pathways by which Oxtr influences NAc activity and behavior, emphasizing the current limitations of interpreting population-level activity without cell-type-specific tools. Finally, we acknowledge the correlational nature of our findings and the lack of causal manipulations besides that of knockout of the Oxtr gene, and we have revised our conclusions to better reflect the scope of our results. We include a discussion of necessary future studies to dissect Oxtr function and its neural substrates more precisely. We hope that the reviewers find the manuscript to be improved, with our conclusions better aligning with our data and highlighting exciting future routes of inquiry in this model.

***Reviewer #1 (Remarks to the Author):***

*In this manuscript, the author show that a developmental loss oxytocin receptor (Oxtr) signaling alters behavioral and neuronal processes that precede the formation of pair bonds in monogamous prairie voles. These are critically important findings that follow up on the initial publications of Oxtr -/- prairie vole papers, which showed that knockouts could form pair bonds.*

*This has created an impression by some in the field that Oxtr is not important for pair bond formation despite decades of evidence that point to the contrary. The authors take a big step towards resolving this paradox by showing that Oxtr -/- differ from wild-type voles in the early stages of pair bond formation. Developmental loss of Oxtr alters the reactivity of the nucleus accumbens, a key node modulating pair bond formation. In my opinion, this manuscript will be viewed as more important than the previous knockout vole papers because it provides a potential explanation for resolving older data with the newer knockout results. That said, the presentation of the results is confusing in places and could be revised to better highlight the importance of the results.*

We thank the reviewer for their positive view on the impact of this work. We have significantly reorganized the presentation of the results to improve the clarity of the manuscript.

*Major comments*

*-In the abstract and discussion I think the authors need to highlight the fact that they find effects of genotypes on processes that affect the formation of pair bonds and that this evidence possibly provides an explanation for apparently divergent results between KO and pharmacology. In a more naturalistic setting, where individuals might only interact for a few minutes (versus the forced pairing that is normally done in neuroscience studies) these effects could be fundamental to altering behavior.*

We agree that the relationship between this study and previous pharmacology work is important to highlight. Briefly, our work here suggests a role for oxytocin receptor signaling in enhancing the formation of partner preference at the earliest stages of pair bond formation. This model is consistent with pharmacological models that investigated the role of acute Oxtr signaling in the development of partner preference. We have now added text to the abstract (line 34) and discussion (lines 837-846 of the track changes manuscript file) to emphasize these points.

*-It appears that analyses in males and females were conducted separately and this may be why they are not directly compared. I am fine with that, but it is confusing when the authors say that different responses in the same behavioral or neural response and these data are in different figures. I think the manuscript would flow better if the authors discussed each part of the pair bond formation (introduction, mating etc) in order and present male and female data in each section. Even if the male and female data were kept in separate figures it would be much easier to keep track of the different findings as opposed to having to remember what was found 3 figures previously.*

We agree that laying out the manuscript in chronological order by assay would be a logical way to present the data and make sex comparisons easier. However, males and females undergo different experimental timelines that are sensitized to each sex (Fig. 1a). Female prairie voles develop pair bonds faster than males (DeVries and Carter, 1999); thus, we conduct the partner preference test (PPT) 6 hours after pairing in females and 5 days after pairing in males. As a result, the contexts and experiences of our male and female prairie voles are only equivalent for

the first assay – the introduction. After debating this for a significant period of time, we are concerned that presenting the male and female data side by side for assays other than the introduction might lead a reader to assume direct comparisons should be made. In addition, our analysis reveals that males and females demonstrate different periods and trajectories of sensitivity to loss of Oxtr function with regard to both behavior and associated neural activity. We therefore chose to emphasize the major findings for each sex, but must account for the fact that they are not symmetrical across the time course of pair bonding. With all this in mind, we originally chose to present the stories for each of the sexes separately. However, because we are able to statistically compare the sexes during the introduction, we have made a new Figure 1 and Extended Data Figure 4 to display and expand the available sex comparisons. We hope this makes the sex comparisons easier to see and interpret. The subsequent Extended Data Figures have been renumbered throughout the text.

*- Extended data 3 was confusing. It seems that behavior conducted on different days was being compared. Also that statement that WT and Oxtr-/- did not differ in calcium responses doesn't match the results in panel 1 and j. Why does line 145 refer back to Extended Fig 3a again?*

Given the many axes of comparison this analysis entails, we appreciate the need for explicit clarity and have revised the text and figure accordingly. Extended Data Figure 5 (previously Extended Data Figure 3) indeed compares behavior and neural responses in the NAc across days in order to examine how female responses to male conspecifics change over time and pair bonding context. Rather than make separate comparisons among the time points (i.e., a Day 0 vs. Day 4 analysis and a separate Day 0 vs. Day 6 analysis), we included genotype, individual vole, and all time points in a single model. This allows us to draw conclusions regarding the differences we observe within and across each comparison. However, in the text, we found it to be confusing to attempt to explain every axis of comparison simultaneously. Instead, we chose to present the results for specific comparisons in sequence: the Day 0 vs. Day 4 comparison (starting at line 235) and the Day 0 vs. Day 6 comparison (starting at line 261). We now indicate which axis is being discussed (lines 237 and 263). Furthermore, we have added text to the x axes in Extended Data Figure 5 to indicate which dataset corresponds to which assay to better relate the data to the timeline presented in panel a.

Regarding the statement that WT and $Oxtr^{1-/-}$ females do not differ in calcium responses, we have clarified the text to indicate that this statement refers specifically to the time points after Day 0. Panels i and j show that WT and $Oxtr^{1-/-}$ females differ in their neural responses during the introduction (Day 0), which are the same findings portrayed in Figure 1. However, there are no differences between WT and $Oxtr^{1-/-}$ female neural responses on Day 4 (blue data) or Day 6 (magenta data).

*-Why not conduct analysis on calcium transient before mounting given the NAc role in motivation (2f suggests possible difference before mating?). Similarly, is it known if females can pace mating bouts? Work from Jill Becker shows this is key for modulation of dopamine release.*

There does appear to be a small difference in signal in the time immediately prior to mounting. However, we compared calcium traces between WT and Oxtr$^{1-/-}$ females and found no significant differences: We compared z-scored ΔF/F values at each time point along the PETH and corrected for multiple comparisons between groups as described in the methods. We found no differences by genotype at any time point pre- or post-mounting. As far as we are aware, there is only one study that has examined paced mating in prairie voles (McCracken et al., 2015). Pacing was accomplished by tethering the male vole. Paced mating did not impact the number of successful matings or later partner preference compared to when females were tethered or neither animal was tethered, although only males were evaluated for partner preference. We agree that this would be an interesting parameter to test for neural responses, although it seems that further characterization of paced mating in female prairie voles is necessary.

*Again in Fig 2 authors state that NAc responses were similar btw WT and -/- yet there are significant differences between partner and stranger in the -/- voles but not wildtype. Perhaps this is because the weaker responses to strangers in the -/- voles does not fit with other results should reduced "pro-social" behavior in the -/-. However, the GCaMP is expressed of a hSyn promoter and different populations of neurons could be responding in different contexts. Luo et al 2024 (Neuropsychopharm) found increased GCaMP responses in NAc during bouts of avoidance.*

WT and Oxtr$^{1-/-}$ females are not significantly different only when considering *partner*-related responses in the NAc. As the reviewer points out, there is a significant difference between partner- and stranger-related activity in Oxtr$^{1-/-}$ females, but not WT females. Furthermore, stranger-related activity in the NAc is significantly decreased in Oxtr$^{1-/-}$ females compared to WT females. We have adjusted the text in that section to make our discussion of these different axes of comparison more explicit. We also agree with the reviewer that the differential activity we are seeing could be driven by different populations of neurons in the NAc, which will be an important direction for future work in this model. This has been added to our discussion on lines 793-794.

*-In figure 4k, do stranger preferring voles differ from wt? If not, is that a surprise?*

In Fig. 4k, stranger-preferring voles do not statistically differ from WT voles. Partner-preferring voles also do not differ from WT voles. Whether or not this is surprising is an interesting question. Broadly, our work reveals that individual naïve WT males vary significantly with regard to their behavior and associated neural activity during initial interactions with a naïve female (Fig. 4b,p,q). Despite this, all WT males eventually displayed robust partner preference. The variability in the behavior of the WT population is consistent with considerable variability in the neural responses to social interaction (Fig. 4j,k,l. Note from the x axis in Fig. 4l that mean normalized AUC values for the WT males span the same range as the Oxtr$^{1-/-}$ values.). It will be interesting to examine partner preference of WT males at an even earlier time point, for example to determine whether WT males with a "stranger-preferring-like" neural profile during the introduction are "slower" to develop a partner preference.

*Minor comments*

*- In lines 18-19, the authors state that the way social attachments and specific social behaviors in ethological contexts form "remains unknown," though they go on to cite several papers that describe mechanisms underlying these behaviors throughout their paper. This statement isn't accurate and should probably be replaced with something more accurate, such as "aspects of these mechanisms remain understudied."*

This has been adjusted.

*- The exclusion rate is relatively high, with about 40% of subjects being excluded. It would be helpful to know exactly how many animals were excluded due to the different reasons listed (poor signal, death, ataxia, etc.).*

We have now added these counts.

*- The exclusion of animals with less robust signal (< 5%) could contribute to an overrepresentation of animals with strong signal in the data.*

Signal < 5% represents very poor signal to noise; calcium-related transients are nearly absent. Histology from some of these animals suggests that the poor signal is often due to lack of virus expression or mistargeting of the fiber relative to virus expression.

*- Some of the statistical analysis relied on transformed data (Box-Cox, log transforms), but lack normality tests and other tests for other assumptions (heterogeneity, etc.).*

For behavior data, we assessed normality of residuals by the D'Agostino-Pearson omnibus (K2) test. Homogeneity of variance was evaluated via the F test. Log transformations were conducted only when the data failed the assumption of normality. If log transformation did not result in normally distributed data, a rank transformation or non-parametric test was used. These details are noted in the methods on lines 1211-1218. In addition, we have now added to the supplemental statistics file in the "Notes" column the reason why any transformation was conducted. For photometry data, we evaluated normality of the data via the Jarque-Bera test (reported on line 1275), and we tested for homoscedasticity via Levene's test (now added on line 1279).

*- Side-by-side contact is described as at least 50% of body contact between voles, though no time threshold is described. Does the contact need to last a specific amount of time or were bouts of this contact recorded instead?*

We did not stipulate a time threshold for side-by-side contact except when noted, such as in Extended Data Figure 12. This has been added to the methods.

*- The Markov chains analyses are a great tool for considering behavioral processes involved in pair bond formation. Was this done for the males also?*

We appreciate the reviewer's suggestion. With our redesign of Figure 1, we have now added Markov models for the males. Markov models for the males according to partner- or stranger-preferring profiles can be found in Extended Data Figure 11q.

*- In line 26, authors state that "prosocial behavior in females in a state-dependent manner…" Prior to this statement, the exact "states" are not described. Changing this to "bonding state" or something similar would add clarity to this sentence.*

We have implemented this change.

*- On line 155-156 the authors should state how long the observation period was for mating to be observed.*

This has been added. Behavior during the timed mating assay was recorded for 30 minutes.

***Reviewer #2 (Remarks to the Author):***

*In "Oxytocin receptor function regulates neural signatures of pair bonding and fidelity in the nucleus accumbens" Long et al correlate neural activity with social behavior during pair bonding interactions. The vole has been an excellent model of prosocial behavior and the creation of the Oxtr-/- line has made an impactful advance in altering understanding of the function of neural oxytocin. This study aims to further characterize and provide a neural basis for how pair bonds are formed. The findings are nuanced and complicated. They analyze either male or female NAc activity during various social interactions across a 10 day period that include partner introduction, several partner preference tests, mating, stranger rejection, and partner separation/reunification. This is an excellent assay design that allows the role of neural activity to be comprehensively analyzed in almost every social permutation. Remarkably, no differences were noted in any social situation except in the duration of initial anogenital investigation of strangers. During novel social activity, oxytocin and the corresponding neural response in the NAc has opposite effect on the sexes promoting investigation in females and inhibiting in males. Moreover, they find a subset of male mutants do not form long term partner preference which can be identified by reduced neural activity in the NAc. There are no neural manipulations to test and provide mechanistic insight, nor is there an advance in understanding the potential sexual dimorphism. The clearest phenotype was in a sub-population of male mutants that continue to show interest in strangers, but the understanding is limited to a correlative readout which is really no more mechanistically informative than the behavioral readout. While I think it is very interesting and important to report the lack of importance of Oxyr in most of the behaviors observed here, I don't think the minor differences reported are critically investigated at this stage to warrant the title and abstract's conclusion. Either, adjust the conclusions to align with the data or experimentally validate to ensure the data supports the new assumptions.*

We thank the reviewer for their positive and constructive feedback. We have implemented the suggested changes to improve the quality of the manuscript and help our conclusions better align with the experiments.

*Concerns:*

*1) While the authors show cannula placement in the individuals, (and it looks like there is a dorsal bias of sp males) there is no characterization of GCaMP expression. It is expected that expression levels will differ which may account for all of the neural activity differences observed. Cannula placement (in females, pp and sp males) shown with the extent and location of viral expression in each individual will help to better interpret the results. As it stands, most of the neural activity differences are peak-differences (latency to respond and slope of response are largely similar) suggesting that where differences are observed it is due to total amount of fluorescence to be captured rather than differences in neural dynamics. This is consistent with both cannula placement and viral expression variability.*

We have now added Extended Data Figure 2 to show cannula placement and viral expression for each animal. There were no consistent differences in viral expression between groups. While several of our findings highlight differences in peak ΔF/F with latency to respond and the slope of the response not different between groups, we also highlight differences in area under the curve that appear to be driven by differences in the offset of activity and/or slope of the decay more so than peak (Fig. 1m, Fig. 4i). Critically, during our analysis of partner preference, we observed *within-animal* differences in partner-related versus stranger-related NAc activity in addition to between-animal and group-level differences (e.g., Fig. 3i). Such a finding is independent of virus expression, as virus expression is the same in a single animal whether it is interacting with the partner or the stranger.

*2) Please expand and explain the role of the observed behavioral differences on the social structure. It appears that any changes in anogenital investigation do not have subsequent effects on pair-bonding or any other metrics of social behavior. If it is not important to the process, then it is an irrelevant part of overall social structure.*

We thank the reviewer for the opportunity to directly address the important issues and implications of our work that they raise. $Oxtr^{1-/-}$ females displayed decreased anogenital investigation as well as decreased non-anogenital investigation and side-by-side contact compared to WT females during the introduction (Extended Data Fig. 4). As the reviewer points out, $Oxtr^{1-/-}$ females display partner preference after only 6 hours, despite the overall decrease in prosocial behavior observed during the introduction. We note that females' avoidance of, or aversion to, novel males represents a distinct behavioral module from partner preference that may impact females' fitness by influencing the initial acquisition of a mate. Furthermore, our findings in males suggest that anogenital sniffing may play a significant role in the early development of partner preference. As others have observed, subtle changes in patterns of social behavior can have significant ramifications for fitness outside of constrained paradigms. We have significantly revised our

Discussion to more explicitly explore the implications of these changes in behavior to the social structure of the vole (lines 743-792).

*3) Please include more raw data of the neural activity during behavior (as supplemental). Where it is shown (Fig. 1d and S9) it does not seem to correlate strongly with the observed behavior. Does a single animal's neural response differ during anogenital investigation (can you see it in the raw data)? Can you see the difference between pp and sp males in the raw data?*

We have included examples of raw data to address this question (Extended Data Fig. 3, Extended Data Fig. 4p, and Extended Data Fig. 10). Specifically, we have added comparisons across genotype and behavioral subpopulations.

*4) Please expand and explain the role of the NAc activity in driving pair-bonding in the social circuit based on your new findings. A toy model would be helpful to better understand how the new findings impact understanding circuits that drive pro-social behavior.*

We appreciate the reviewer's observation that the role of the NAc in driving prosocial behavior remains unresolved with this work. Indeed, the role of neuronal activity (as opposed to, e.g., dopaminergic or oxytocinergic activity) within the NAc in driving social behavior is poorly understood within the field at large. New studies demonstrate that specific neuronal populations in the NAc drive opposing patterns of prosocial and antagonistic behavior (for example, see Golden et al., 2016, *Journal of Neuroscience*; Le Merrer, 2024, *Biological Psychiatry*; de Jong et al., 2019, *Neuron*; Collins et al., 2020, *Biological Psychiatry*; among others). With these considerations in mind, we might speculate that loss of oxytocin signaling impacts one or multiple of these populations. However, given that our work analyzes population-level activity, we hesitate to create specific models so as to avoid creating hypotheses that are not founded upon data from this study. In addition, as Reviewer 3 points out, effects of loss of Oxtr on behavior and neural activity may be parallel effects or may be driven by indirect effects on upstream regions, sensory processing, development, etc. We have revised our discussion to detail these various possibilities and highlight the need for additional experiments.

*5) Almost all of my concerns (above) could be mitigated by including standard chemogenetic experiments to manipulate neural activity (enhance or impair) and confirm that it alters behavior as predicted by the correlations observed. With the success of the fiber photometry here, it does not seem technically challenging to include such important validations.*

We thank the reviewer for this suggestion. We agree that these would be fascinating experiments. However, we do not yet have the genetic access in this model system to render them interpretable, and thus feel they are beyond the scope of this work. Because we are considering only population activity—without regard to specific subpopulations—it will be challenging to establish a robust relationship between NAc activity and specific behavioral displays. Previous and ongoing work in other model organisms shows that distinct neural populations within the NAc can drive opposing behavioral displays (see citations noted in point 4). Relatively few studies

have focused on the activity of the primary output neurons (medium spiny neurons) or interneuron subtypes within the NAc, so the role of these different populations in social behavior presents a particular challenge to interpreting the results of manipulations that we can only conduct without cell-type specific targeting. Our focus on the function of Oxtr signaling also complicates interpretation of such an experiment. We do not know if changes in NAc activity are due to Oxtr function within the NAc or are the result of changes in activity in upstream regions (see Amadei et al., 2017, *Nature*). Thus, even if we were to see a change in behavior, it is difficult to interpret any causal mechanism with regard to Oxtr function, given the nonspecific manipulation of NAc activity. Another possibility, as noted by Reviewer 3, is that behavioral changes and neural activity changes are parallel effects of loss of Oxtr that could be driven by changes in upstream regions affecting sensory processing and social motivation. Overall, the reviewer's concern is important, and we have revised our Discussion Section to incorporate these points and to discuss the need for future mechanistic studies to understand how and where Oxtr signaling impacts neural activity in the NAc and how such changes impact attachment behavior (lines 801-836).

***Reviewer #3 (Remarks to the Author):***

*This study investigated how oxytocin receptor (Oxtr) function affects pair bonding behaviors and neural activity in prairie voles. The researchers used fiber photometry to measure real-time neural activity in the nucleus accumbens while male and female prairie voles, both with and without Oxtr, interacted with potential mates. They found that Oxtr has sex-specific effects. In females, Oxtr regulates initial social investigation of novel males. In males lacking Oxtr, it could be predicted whether an individual would eventually prefer their partner or a stranger based on their neural activity patterns during their very first interaction. This research is important and advances our understanding of Oxtr mechanisms in prairie vole social behavior. However, there are several considerations worth discussing:*

We thank the reviewer for their positive and constructive feedback. We have incorporated their feedback throughout the manuscript.

•*The findings in females describe both behavioral changes (e.g., reduced investigative and affiliative interactions in Oxtr1-/-) and neural activity changes (e.g., decreased NAc activity during anogenital investigations). However, the connection between these two sets of findings is not fully explored. Does reduced NAc activity directly cause the behavioral changes, or are they parallel effects of Oxtr loss?*

This is an excellent question that was also raised by Reviewer 2 (see our response to Reviewer 2 point 4 above). Broadly, the role of neural activity in the NAc for social behavior remains underexplored, as relatively few studies have focused on the activity of medium spiny neurons or interneuron subtypes within the NAc. Here, establishing a relationship between NAc activity and specific behavioral displays remains difficult given that we are looking at population activity. Distinct neural populations within the NAc can drive opposing behavioral displays (Golden et al.,

2016, *Journal of Neuroscience*; Le Merrer, 2024, *Biological Psychiatry*; de Jong et al., 2019, *Neuron*; Collins et al., 2020, *Biological Psychiatry*; among others). Furthermore, the relationship between Oxtr signaling and levels of neural activity remains to be determined. For example, we do not know if changes in NAc activity are due to Oxtr function within the NAc or are the result of changes in activity in upstream regions (see Amadei et al., 2017, *Nature*). Therefore, directly manipulating NAc activity broadly does not necessarily inform our understanding of Oxtr function even if this were to result in a change in behavior.

In addition, as the reviewer notes here and as we address above, "another possibility is that behavioral changes and neural activity changes are parallel effects of loss of Oxtr that could be driven by changes in upstream regions affecting sensory processing and social motivation. We have revised our Discussion Section to incorporate these points and to discuss the need for future mechanistic studies to understand how and where Oxtr signaling impacts neural activity in the NAc and how such changes impact attachment behavior (lines 801-836)."

•*Since oxytocin and vasopressin can bind to each other's receptors, does the absence of oxytocin receptors potentially disrupt vasopressin signaling pathways in other brain regions?*

This is certainly a possibility. As this reviewer notes later, constitutive loss of Oxtr throughout the body and throughout development may have both direct and indirect effects on neural activity and behavior. An example of an indirect effect may be changes in oxytocin's interactions with vasopressin receptors. The $Oxtr^{1}$ mutation leads to a loss of oxytocin binding to the oxytocin receptor in our prairie voles; hence, it is possible that an excess of unbound oxytocin may bind to vasopressin receptors. We have shown that there are no detectable differences in vasopressin 1a receptor binding in $Oxtr^{1-/-}$ prairie voles; however, we did determine that loss of Oxtr alters expression of genes associated with the CRH pathway in the NAc and that Oxt and Avp levels are reduced in the PVN in $Oxtr^{1-/-}$ mutants (Sharma et al., 2025). We have added such considerations to our Discussion Section.

•*More detail on how the authors define affiliative behavior should be in the beginning of results or in their table.*

We thank the reviewer for noting this oversight. In this study, we consider side-by-side contact to be an affiliative form of behavior. This has been added to the table, and instances of this term in the text have been clarified.

•*The authors report sexual behavior and aggression inconsistently across experiments. If the behavior was not observed in certain experiments, it should be explicitly stated.*

Sexual and aggressive behaviors were scored for all assays; however, they were rarely observed or not observed in specific assays. We have clarified this question about the presentation of results in the Methods.

•*Sex difference comparisons are confusing since the male and female data are presented separately. Why were the sexes not compared statistically? This makes it difficult to interpret any kind of sex difference in the data sets.*

This is an important point that was also raised by Reviewer 1. Briefly, female prairie voles develop pair bonds faster than males (DeVries and Carter, 1999); thus, we conduct social behavior tests along timelines that are sensitized to each sex. As a result, the contexts and experiences of our male and female prairie voles are only equivalent for the first assay – the introduction. Because of this, we originally chose to present the stories for each of the sexes separately. However, we have made a new Figure 1 and modified our Extended Data Figures to display and expand the available sex comparisons from the introduction.

•*In females, was sexual behavior affected during the 6 hours of cohabitation, or did the authors notice any differences in latency to receive mounting or intromissions in early pairings?*

In this study, we used a timed mating paradigm. We induced behavioral estrus following the pairing of naïve animals and assessment of partner preference in females (Sharma et al., 2025). Thus, females are not sexually receptive upon pairing. Concordantly, across all of our recorded animals, we observed only 4 instances of mounting during introductions. No mounting instances progressed to intromission. We did not record behavior during the 6 hour cohabitation; thus, we cannot state whether mating during this time period differed between wild-type and $Oxtr^{1-/-}$ females.

•*While the study shows that Oxtr loss in females reduces both nucleus accumbens activity and social investigation of novel males during early bonding, the discussion should better explain the potential mechanisms underlying these disruptions.*

We have several hypotheses on this topic, and we have added such potential mechanisms to the discussion section. As we note in response to Reviewer 2, we hesitate to speculate about specific mechanisms that are not founded upon our data and focus instead upon broader possibilities. These include the possibility that loss of Oxtr influences the activity of specific subpopulations of neurons within the NAc (lines 793-794) and/or the intrinsic activity of these neurons (lines 803-815). In addition, as noted in the reviewer's point below, Oxtr may influence the activity of afferent projections to the NAc (lines 815-818) or processing in regions upstream of the NAc (lines 820-822). Loss of Oxtr may influence compensatory neuromodulatory signaling (lines 822-826), and constitutive loss of Oxtr may impact the development of social behavior circuits and/or adult neuromodulatory signaling (lines 826-830). We feel these possibilities open many exciting routes for future work with this model.

•*The observed behavioral changes in Oxtr1-/- prairie voles could result from factors like altered sensory processing, reduced motivation, or compensatory mechanisms, rather than direct effects of Oxtr loss. In females, the lack of differences in NAc activity during non-anogenital sniffs or side-*

*by-side contact suggests that Oxtr's role may be limited to specific social behaviors rather than broadly influencing prosociality.*

Indeed, we agree that Oxtr may influence specific social behaviors that contribute to pair bonding. Our finding that $Oxtr^{1-/-}$ females exhibit reduced NAc activity specifically related to chemoinvestigation suggests that Oxtr plays a role, at least in relation to the NAc, in the processing of these specific cues either within or upstream of the NAc. We note, however, that all forms of investigative and affiliative behavior were reduced in $Oxtr^{1-/-}$ females during the introduction. Whether or not the effects we observed with chemosensory-related neural activity contribute to broad changes in prosociality remains to be determined in future work. For example, experiments could test how altering activity specifically during chemosensation affects global patterns of prosocial behavior and associated activity in other regions and, in parallel, how direct modulation of Oxtr signaling affects patterns of activity in the NAc and potentially behavior. We have taken the opportunity to expand our discussion of these ideas and the potential indirect effects of loss of Oxtr (lines 801-836).

•*In the male data, the sample sizes are small (subgroups of 5 and 3 voles), making it difficult to draw robust conclusions about distinct behavioral subpopulations. Statistical limitations should be discussed. The authors say that the loss of Oxtr in only 3 non-partner-preferring males revealed a population that showed no difference in NAc activity during interactions with partners versus strangers. Is it typical for all WT animals to form partner preferences? The observed behavior in these 3 males could simply reflect normal variation rather than a meaningful subpopulation.*

This is an important consideration. Given our observations, we anticipate that larger cohorts may reveal even more nuanced variations in the behaviors and patterns of activity in Oxtr mutant males (see new lines 763-765). While we typically observe some variability in partner preference in WT animals, it is not unusual for most, if not all, of a cohort to demonstrate robust preference for partners. Our recent work, in separate cohorts, shows that even with behavioral variability among WT males, there is a clear subset of $Oxtr^{1-/-}$ males that display a stranger preference (Sharma et al., 2025). With these considerations in mind, we posit that the stranger-preferring $Oxtr^{1-/-}$ males are indeed a behaviorally distinct subpopulation; however, we are enthusiastic to continue to explore these populations with larger cohorts. In addition, it would be interesting to identify naturally occurring stranger-preferring WT males and to compare their behavior and neural activity to $Oxtr^{1-/-}$ stranger-preferring males.

•*It may be difficult to correlate the partner-stranger AUC differences if the WT males are already at a ceiling (strong preference for partners) or floor (minimal interaction with strangers), leaving little room for variation to detect meaningful relationships.*

We agree with the reviewer that it may have been difficult to detect meaningful relationships given that all WT males exhibited robust partner preference; however, as there is still variability among WT males in the amount of time they spend interacting with the partner (Fig. 3b), we felt that it is important to determine if there is a correlation between variability in behavior and neural activity. Intriguingly, this analysis suggests a relationship is only observed in $Oxtr^{1-/-}$ males, as opposed

to—for example—different correlations for WTs versus mutants. Furthermore, despite the variability in partner- versus stranger-related neural activity in WT males, all WT males displayed robust partner preference (Fig. 3i). As we discuss, this suggests that the difference in activity in the NAc is not what is driving behavior and that Oxtr function facilitates prosocial behavior directed toward the partner independent of NAc activity. In fact, our results suggest Oxtr function may decorrelate NAc activity from partner-directed prosocial behavior. Lastly, related to this reviewer's previous comment, it would be interesting to increase the number of WT males in future studies in order to identify stranger-preferring WT males and to compare their relationships between neural activity and partner- versus stranger-directed behavior.

## RESPONSES TO REVIEWER COMMENTS

We appreciate the reviewers' positive reception of our revision and the opportunity to further address their concerns. We have refined our language throughout the manuscript to avoid implying a causal mechanism between functional Oxtr and neural processing. We instead focus on how the *loss* of Oxtr affects behavior and neural activity. We have also reorganized the discussion to articulate more clearly how our findings inform current models of Oxtr function. To summarize our interpretations, we have added a new figure. We further note that future work will be needed to parse the causal consequences of Oxtr on behavior and neural activity. Finally, we have more explicitly addressed the robustness and limitations of our correlational analyses.

**Reviewer #1 (Remarks to the Author):**

*This is a very strong revision. All of my comments have been addressed well by the authors.*

We are grateful for the reviewer's thoughtful feedback and glad that our revisions have addressed their concerns.

**Reviewer #2 (Remarks to the Author):**

*While there have been some changes that make the paper slightly easier to read and understand, my overall concerns remain. The title and abstract still claim a novel and mechanistic relationship between NaC activity, behavior, and Oxtr. The paper does not support these claims. The authors are still unable to make that relationship clear (either by a model or a clear textual conclusion) in a way that is useful as a base for the neuroscience community to follow up upon or for a general reader to determine the extent to which oxytocin can be thought of as a 'love drug'. I am not suggesting that nuanced results are not interesting, but here we are served a buffet of observations without much synthesis or understanding of how these data advances our knowledge of how this neuromodulator works (either in the brain or behaviorally). Moreover, the most potentially interesting part of this study is the stranger preference displayed by the mutant males. But the robustness of these conclusions are questionable because the number of males falling into this category is so small (N=3), leaving also a very small partner preference male group (N=4). I am disappointed that the authors did not supplement this finding with another cohort of data. Finally, it was helpful for me as a reader to see additional ethograms of the raw data of neural activity and behavior (figure 1d and extended figure 3) however, even in these best case examples the relationship between the neural activity and the actual behavior is not correlated in a way that suggests a relationship except the correlation occurring during the initial sniff bout –*

*which is not really the focus of this study. I believe that the conclusions need to be softened to better align with the data, and the author's understanding of the role of this data to impact our current model of the function of oxtr be spelled out. As it stands it is an incremental advance.*

We appreciate the reviewer's points. Regarding claims of causality, we agree that our language could be adjusted. We have revised the title, abstract, results, and discussion to focus on how the loss of Oxtr affects behavior and neural activity, rather than inferring how functional Oxtr influences these processes based on knockout data. These revisions clarify the distinction between our conclusions and the hypotheses we propose regarding Oxtr function. We believe these changes strengthen the alignment between our conclusions and the evidence presented.

We have also revised our discussion section to better highlight the ways in which our data inform current models of Oxtr function. First, monogamous mammals are known to show fewer physical and behavioral sex differences (Kleiman, 1977). Our work indicates that the loss of Oxtr yields opposing effects on prosocial behavior directed towards novel potential partners in male and female prairie voles, suggesting that functional Oxtr contributes to reducing sexual dimorphisms in behavior. Second, our work reveals that loss of Oxtr particularly impacts neural activity in the NAc during chemosensory investigation (i.e., anogenital sniffing) of novel partners. This points to a potential role for functional Oxtr in shaping neural responses to olfactory cues from conspecifics and suggests new avenues for investigating how such cues drive plasticity that supports pair bond formation. Third, our behavioral and neural findings indicate that loss of Oxtr influences *early,* but not late, social dynamics between mates. Functional Oxtr may reduce behavioral variability and promote a more efficient trajectory of pair bond formation.

We have now added Figure 5 to summarize these interpretations. Furthermore, we acknowledge the limitations of constitutive knockout models, and we note the wide range of physiological effects that may result from the loss of Oxtr (starting at line 444). While future experiments will address these effects in greater detail, our work here provides novel insight into the role of Oxtr in facilitating pair bond formation.

Regarding the robustness of our correlational findings, we readily acknowledge the limited sample size and now explicitly note this in line 413. With respect to the correlation between NAc signal and behavior, our raw data panels and Extended Data Figure 12f-n show that NAc calcium activity often increases when a social stimulus is introduced to the cage, which is not necessarily correlated with behavior. This transient increase in activity is consistent with prior reports demonstrating that VTA-NAc projection activity and dopamine release in the NAc increase upon introduction of a stimulus to the test arena and subsequently decay over repeated social bouts (Gunaydin et al., 2014, Figure 1G and S1B,C; Robinson et al., 2002, Figure 3; Dai et al., 2022, Figures 1 and 2). The specific sniff-related activity we observe is distinct from the non-specific increase in NAc activity that occurs with the presentation of a social stimulus. The accumbens integrates input from limbic regions with neuromodulatory input from the VTA, dorsal raphe, and hypothalamus, indicating complex convergence of social information. Much remains to be understood about the specific stimuli that engage NAc neuronal populations and how activity modulates the likelihood, efficiency, or vigor of social behavior (Floresco, 2015).

**Reviewer #3 (Remarks to the Author):**

*The revised manuscript represents an improvement that addresses a lot of my concerns. However, given the acknowledged limitations of fiber photometry and the inability to establish*

*whether NAc activity changes drive behavioral outcomes or are simply parallel consequences of Oxtr deletion, I recommend the authors adjust their language to better reflect the correlational nature of their findings (e.g., Oxtr "regulates" neural processing imply causal mechanisms that cannot be established).*

We thank the reviewer for their positive reception of the revision. As noted in our response to Reviewer 2, we agree that our language can be adjusted, and we have made such revisions to the title, abstract, results, and discussion.

## RESPONSES TO REVIEWER COMMENTS

**Reviewer 2**

*The interpretation and synthesis of the data are slightly more flushed out in the current manuscript. The new figure 5, though complicated to follow without reading the text, is a helpful summary. While the interpretations are largely based on correlative results, the data itself is rigorously presented so others can evaluate the conclusion and impact. I expect publication of the study in Nature Communications will now provide a platform for the authors and others to continue flushing out mechanistic understanding of oxytocin's role in prosocial behavior.*

We thank the reviewer for their positive reception of the revision.
