# Supplementary material for: Loss of oxytocin receptor function disrupts neural signatures of pair bonding and fidelity in the nucleus accumbens
Source: Nat Commun. 2026 Sep 12;17:10755. doi: 10.1038/s41467-026-76876-8 (PMC13661945; doi:10.1038/s41467-026-76876-8)
Supplement: Supplementary file 3 [file 41467_2026_76876_MOESM3_ESM.pdf]

Description of Additional Supplementary Files

File Name: Supplementary Data 1
Description: Statistics
